# tidyexposomics: integrated exposure-omics analysis powered by tidy principles

**DOI:** 10.1093/bioadv/vbag220

**Published:** 2026-08-08

**Authors:** Jason Laird, Thomas Hartung, Fenna C M Sillé, Alexandra Maertens

**Affiliations:** Center for Alternatives to Animal Testing (CAAT), Bloomberg School of Public Health and Whiting School of Engineering, Johns Hopkins University, Baltimore, MD 21205, United States; Center for Alternatives to Animal Testing (CAAT), Bloomberg School of Public Health and Whiting School of Engineering, Johns Hopkins University, Baltimore, MD 21205, United States; CAAT-Europe, University of Konstanz, Konstanz, 78457, Germany; Center for Alternatives to Animal Testing (CAAT), Bloomberg School of Public Health and Whiting School of Engineering, Johns Hopkins University, Baltimore, MD 21205, United States; Center for Alternatives to Animal Testing (CAAT), Bloomberg School of Public Health and Whiting School of Engineering, Johns Hopkins University, Baltimore, MD 21205, United States

## Abstract

**Motivation:**

Environmental exposures shape health across the life course, influencing molecular pathways, disease susceptibility, and therapeutic response. However, integrating exposome and multi-omics data poses several challenges, ranging from extensive analytical steps to ensuring trends are consistent across studies.

**Results:**

To address this challenge, we developed tidyexposomics, an open-source R package that delivers an end-to-end, tidyverse-native workflow including ontology-based exposure annotation, quality control, association testing, stability assessment, multi-omics integration, network analysis, and functional enrichment. The package offers intuitive Application Programming Interface (API), modular functions, and extensive visualization tools to facilitate flexible analyses. Comprehensive analytical step tracking and result export functions enable reproducibility and consistent reporting of downstream results. By combining systematic preprocessing, statistical modeling and biologically informed interpretation in one package, tidyexposomics streamlines exposure-omics analysis.

**Availability and implementation:**

The GitHub repository for tidyexposomics is available at: https://github.com/BioNomad/tidyexposomics

**Pre-processed example data are available at:**

https://doi.org/10.5281/zenodo.17049350

## 1 Introduction

The course of disease and mortality is governed by complex interactions between both environmental factors and genetics. Exposomics seeks to characterize all physical, chemical, biological, and (psycho)social influences that impact biology from pre-conception to death (Wild 2005, Miller et al. 2025). Several tools currently exist to analyze exposomics data including: rexposome, omicRexposome, and ExposomeX (Hernandez-Ferrer et al. 2019, Wang et al. 2025). These tools provide functions to pre-process data, associate exposures with outcomes and omics features, principal component analysis (PCA), feature and sample clustering, and multi-omics integration (Hernandez-Ferrer et al. 2019, Wang et al. 2025). These packages currently rely on S4/R6 class structures and Bioconductor-specific syntax that can be unfamiliar to researchers trained in tidyverse workflows, where tabular data is manipulated through intuitive, natural language-like commands (Hernandez-Ferrer et al. 2019, Wang et al. 2025). Tidy principles have been progressively extended across Bioconductor, starting with plyranges, which introduced a grammar of genomic data transformation, and tidybulk, which established a tidy framework for modular bulk transcriptomic analysis (Lee et al. 2019, Mangiola et al. 2021). Recent packages, like tidyomics, have unified these efforts by allowing manipulation of complex multi-omics data structures (Hutchison et al. 2024). However, the extension of these tools has yet to be implemented in exposomics software. Object management can similarly be challenging, given exposome and multi-omics data are stored across separate objects requiring manual coordination to ensure sample alignment and consistent preprocessing. Existing tools lack built-in mechanisms to track analytical decisions, which can make it difficult to reproduce or audit complex pipelines (Hernandez-Ferrer et al. 2019, Wang et al. 2025). Most exposomics packages support association testing but assessing the robustness of findings across different preprocessing choices or through resampling requires custom code (Hernandez-Ferrer et al. 2019, Wang et al. 2025). Pathway enrichment is used to connect statistical results to biological interpretation, but remains fragmented, requiring multiple tools. Consolidation of exposure associations across cohorts requires mapping exposure names, but no exposomics tools currently support this. This can result in increased ambiguity in reporting, making it harder to harmonize exposure association findings (Zhang et al. 2021). Here we present tidyexposomics, an R/Bioconductor package that addresses these limitations through a tidyverse-compatible framework built on the MultiAssayExperiment container, a unified object for storing experimental data. The package integrates exposome and multi-omics data within a single object, tracks analytical decisions, provides sensitivity analysis support, and enables ontology-driven reporting, all through an intuitive piped API (Table 1).

**Table 1:** Exposome software comparison table.

Feature	rexposome	omicRexposome	ExposomeX	tidyexposomics
Tidyverse syntax and data accessors	X	X	X	✓
Unified data container	ExposomeSet	MultiDataSet	Split across packages	MultiAssayExperiment
Ontology annotation	X	X	X	✓
Automatic step tracking	X	X	X	✓
Association analysis	✓	✓	✓	✓
Omics integration	X	✓	✓	✓
Sensitivity analysis	X	X	X	✓
Network analysis	X	X	X	✓
Enrichment analysis	X	X	X	✓
Result export to Excel	X	X	X	✓

## 2 Features of tidyexposomics

**Figure 1:**
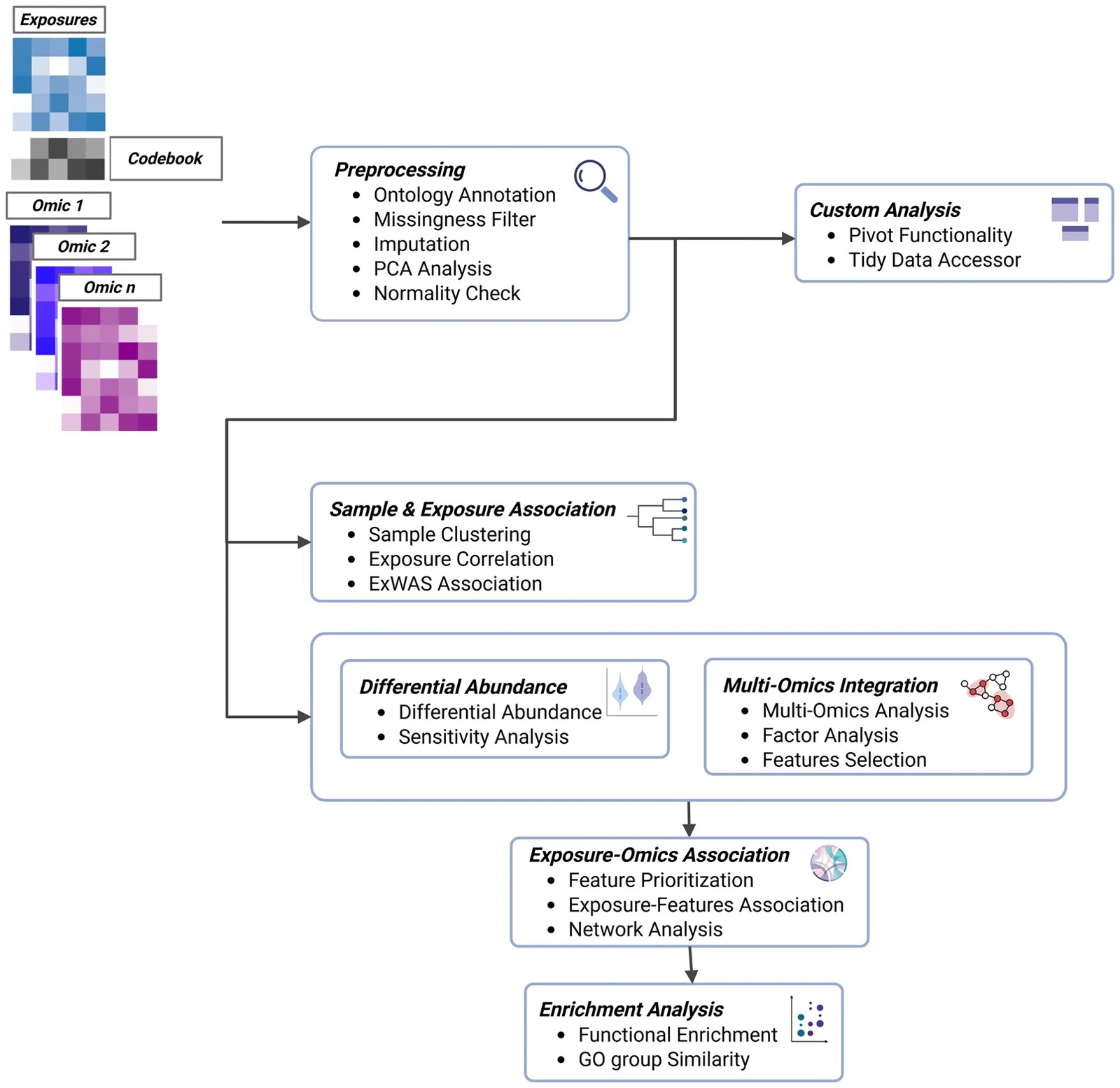
Overview of the functionality provided by the tidyexposomics package. Omics, exposure, and a codebook of exposure metadata are integrated in a MultiAssayExperiment object. Quality control includes functionality to filter out variables with high missingness, impute missing values, perform dimensionality reduction and confirm normality. Samples clustering analysis identifies trends based on exposure profiles and association testing assesses relationships between exposures and exposures and outcomes. Differential omics feature analysis and multi-omics integration support allows users to assess relationships between omics and an outcome variable. Network analysis is also supported, highlighting highly connected exposures. Biological interpretation is offered through functional enrichment analysis, and custom analyses are provided through several pivot functions and tidy data accessors. Created in BioRender. Laird, J. (2026) https://BioRender.com/denq254.

Supplemental File 1 describes all features and statistical methods applied in tidyexposomics, while Fig. 1 summarizes the available features. To start using tidyexposomics, users need three inputs: (i) exposure matrix (samples × exposure), (ii) one or more omics data matrices (samples × features); and (iii) a codebook containing exposure variable metadata such as the units, exposure period and limits of detection (LOD). An ontology mapping R shiny app is provided to map exposure variables to ontology terms in Human Phenotype Ontology (HPO), Chemical Entities of Biological Interest (ChEBI), and Environmental Conditions, Treatments, and Exposures Ontology (ECTO). Individual ontology terms are then aggregated into broader categories. The updated codebook, exposure, and omics data are stored in a MultiAssayExperiment object; an object designed to efficiently store metadata and assay data from multiple experiments in the Bioconductor ecosystem (Ramos et al. 2017). Core preprocessing steps include missingness filtering, imputation, variance/expression filtering of omics features, exposure normality assessments, exposure variable transformations, and PCA. Users explore sample-exposure relationships either through clustering, correlation networks and user-defined exposome scores. Correlation and linear modeling approaches are provided to assess exposome-wide association study (ExWAS) results for both exposures and omics features. To interrogate omics trends, users have the option to perform differential abundance analysis with stability assessment or multi-omics integration to identify latent factors of interest. This package further facilitates exposure-omics network analysis using correlation networks to identify both exposures that are central in the exposure-omics network and exposures that are associated with omics features that are central within omics-feature-only networks. Biological interpretability is supported through functional enrichment analysis on associated features. At each step, multiple visualization functions are provided to enable interrogation of results (Fig. 1). Several pivot functions are provided to extract assay, sample or feature-level data for custom analyses. To enable auditing and reproducibility, analytical steps are recorded in the metadata of the MultiAssayExperiment object and users have the option to print these for record-keeping purposes. Finally, functionality to export stored results to a spreadsheet is provided. Together, tidyexposomics builds on several foundational Bioconductor packages, limma powers the differential abundance and exposure-omics testing (Ritchie et al. 2015), mixOmics helps support integration of several omics layers (Rohart et al. 2017), and tidybulk informs the tidy interface design to handle these omics layers (Mangiola et al. 2021).

To demonstrate how users interface with tidyexposomics, we compare representative workflows, imputation, normality assessment, transformation, and ExWAS, as implemented in rexposome and tidyexposomics:

# rexposome

exp       <- imputation(exp)

nm        <- normalityTest(exp)

exp_std <- standardize(exp, method = “normal”)

exp_pca <- pca(exp_std)

fl_ew   <- exwas(exp,

formula = asthma ∼ sex + age,

family = “binomial”)

# tidyexposomics

expom <- expom |>

run_impute_missing(

exposure_impute_method = “missforest,”

exposure_cols        = exp_vars

)  |>

run_normality_check() |>

transform_exposure(

transform_method = “boxcox_best,”

exposure_cols   = exp_vars

)  |>

run_pca() |>

run_association(

source    = “exposures,”

outcome     = “hs_asthma,”

feature_set = exp_vars,

family    = “binomial”

)

In rexposome, each step returns a new S4 object of a distinct class, requiring the user to manage these separately and apply class-specific accessor functions to retrieve results. By contrast, tidyexposomics returns a single MultiAssayExperiment object throughout. This enables the entire workflow to be expressed as a single pipe, which reduces variable assignment while keeping the command count equivalent. The unified container design also means analytical steps are automatically tracked within the object metadata. To understand the performance capabilities of tidyexposomics we benchmarked runtime and memory usage for exposure-omics association testing against rexposome/omicRexposome across varying numbers of samples (10, 25, 50, 75, 100) and omics features (100, 500, 2500, 5000). Each condition was run once and performance was measured using the bench::mark(), which reports median wall-clock time and total memory allocated during execution (Vaughan 2025). We found that tidyexposomics executed the analysis faster across all tested sample sizes and feature counts. Memory use on small datasets was slightly higher than in rexpsome/omicRexposome, but this difference diminished with increasing sample size and number of features. Benchmark results are described in full in Supplemental File 3.

## 3 Application example

**Figure 2:**
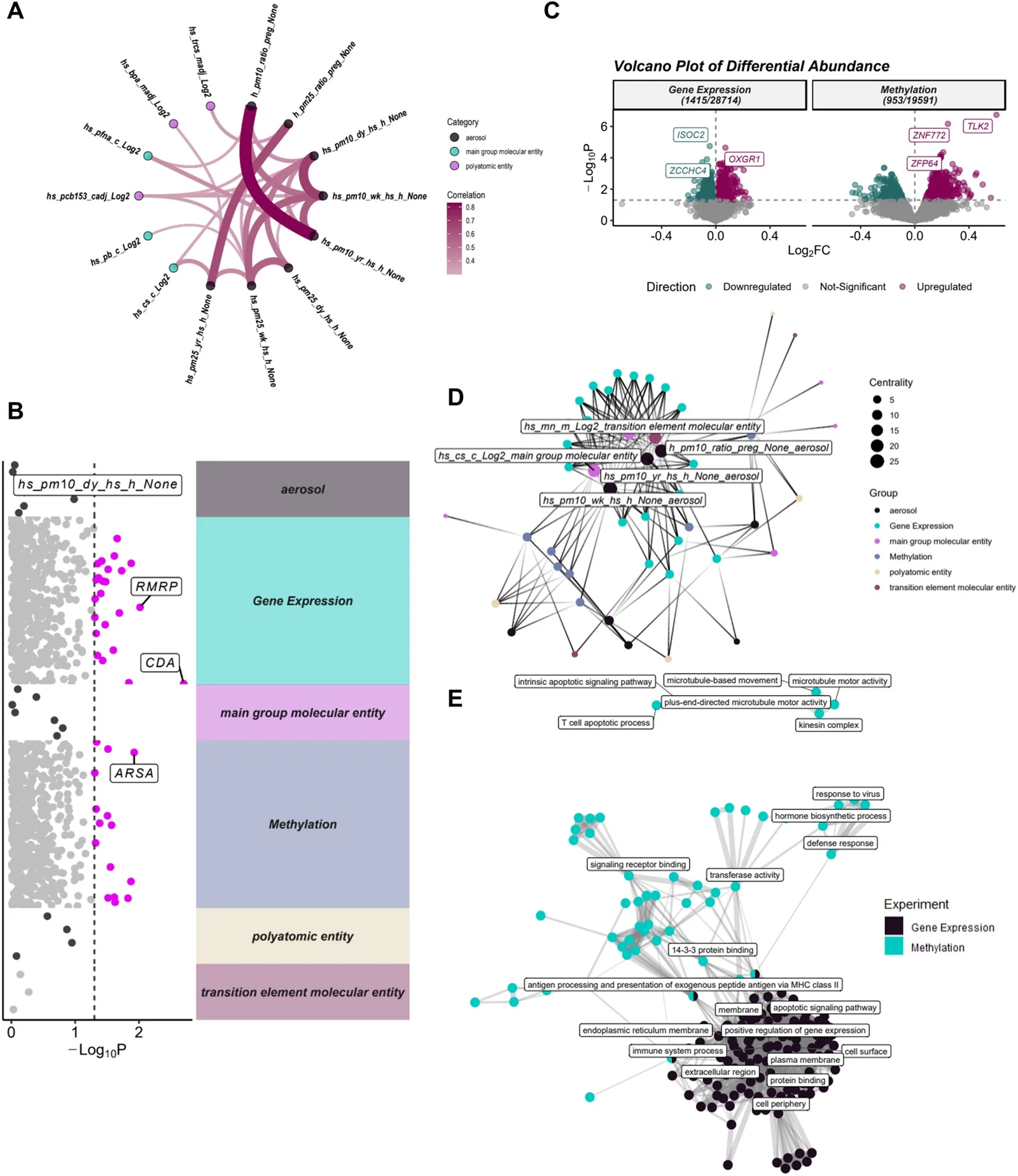
Example visualizations offered in the tidyexposomics package. (A) Exposure correlation circos plot, where the edges are colored by the correlation and the nodes are colored by the exposure category. (B) ExWAS manhattan plot displaying the -Log_10_  *P*-values for the association between a set of exposures and asthma status. (C) Volcano plot of the differentially abundant features faceted by omics assay and colored by the change direction of the association, with the top differentially abundant features labeled. (D) Exposure-feature correlation bipartite graph colored by exposure category and omics assay, nodes sized by centrality degree, and with the top 5 most central exposures labeled. (E) Functional enrichment term network plot displaying terms as nodes connected by edges which represent shared molecular features. Nodes are colored by the omics assay driving the enrichment term, with the top terms labelled.

We applied tidyexposomics to an ISGlobal Exposome Data Challenge 2021 subset of 114 low socioeconomic status children to identify exposures and omics features associated with asthma status (n = 14 cases, n = 100 controls) (Supplemental File 2) (Maitre et al. 2022). Ontology annotation enabled exposures to be grouped into broad categories, such as aerosols, transition metals, and polyatomic exposures. After quality control, one exposure variable with greater than five percent missingness was removed and the remaining variables were imputed. Low expression/variance omics features were filtered out, resulting in 19 591 methylation, 28 714 gene expression features. Box-Cox transformation improved normality and PCA was used to identify six sample outliers, which were removed from further analysis (Supplementary File 2, Fig. 8). Spearman correlation analysis revealed that particulate matter and perfluorohexane sulfonic acid (PFHxS) exposure were associated with major principal components (Supplementary File 2, Fig. 9). Among exposures, particulate matter exposures were highly correlated (Fig. 2A).

Dynamic tree cut sample clustering revealed three groups largely driven by aerosol exposures (Supplementary File 2, Fig. 11). ExWAS did not identify any exposures significantly associated with asthma status (*P* < 0.05), the lowest ranking *P*-values were associations with particulate matter (OR = 0.62, *P* = 0.10) and Polychlorinated biphenyl-153 (PCB-153) (OR = 0.60, *P* = 0.11) (Fig. 2B, Supplementary File 2, Fig. 13). Child metal exposures were combined into several types of exposome scores, and only the variance-based score (variance of the exposures in the exposure set) was associated with asthma status (*P* < 0.05) (Supplementary File 2, Fig. 15). Differential abundance analysis revealed nominally significant features (*P* < 0.05) across omics layers: 1415 gene expression, 953 methylation features were found to be differentially abundant (Fig. 2C, Supplementary File 2, Fig. 16). Sensitivity analysis demonstrated that 34 gene expression and 12 methylation features had a stability score below 0.8 (0 indicating not stable, and 1 indicating extremely stable), suggesting very few of these associations are stable (stability score incorporates the number of times an association is significant and the consistency of the effect and is described in greater detail in Supplemental File 1, Section 5.2).

To identify coordinated biological signals across omics layers, multi-omics integration analysis, using multiple co-inertia analysis, identified five latent factors where one component was significantly associated with asthma status (*P* < 0.05) (Mattessich et al. 2024) (Supplementary File 2, Fig. 19). This latent factor was filtered to retain features with high contributions to these factors (> 95 percentile), identifying 2416 features. Exposure-omics correlation analysis of these features found 2607 significant associations (|ρ| ≥ 0.2, *P* < 0.05). Network analysis showed that prenatal particulate matter exposures, prenatal manganese and childhood cesium exposure had the most edges within the exposure-omics bipartite graph, while childhood lead and prenatal BPA exposure were additionally prioritized using the feature-only graph (Fig. 2D, Supplementary File 2, Figs. 21 and 24). Functional enrichment analysis of exposure-correlated omics features found methylation features were associated with immune signaling and gene expression with immune signaling and hypoxia (Fig. 2E, Supplementary File 2, Fig. 27). Together, these results illustrate how tidyexposomics enables insight into how exposure may affect biology.

## 4 Discussion

Here we present tidyexposomics, an open-source R/Bioconductor package that builds on earlier frameworks and extends functionality to be tidyverse-native and to support ontology annotation, network analysis, sensitivity analyses, functional enrichment The tidyverse-native design ensures interoperability with the broader R ecosystem, and the modular architecture supports additional analysis modules to be added on top of existing functionality. This package complements ongoing exposome and systems toxicology initiatives by providing functionality to increase interoperability and comparability of exposome and multi-omics analysis results. By leveraging ontology-based annotations, we hope that semantic alignment across studies can be used to facilitate the construction of exposome atlases, which hold promise to unravel the contributions of exposure to disease (Young et al. 2025). The current implementation is limited to cross-sectional analyses with single time point exposures. Future development will prioritize support for temporal modeling of longitudinal data, mixtures analysis to assess combined exposure effects and regularized modeling to improve stability in high-dimensional data analysis. To this end, tidyexposomics aims to lower technical barriers between environmental epidemiology, molecular biology, and bioinformatics communities.

## Supplementary Material

vbag220_Supplementary_Data
