# Supplementary material for: tidyexposomics: integrated exposure-omics analysis powered by tidy principles
Source: Bioinform Adv. 2026 Aug 8;6(1):vbag220. doi: 10.1093/bioadv/vbag220 (PMC13532756; doi:10.1093/bioadv/vbag220)
Supplement: Supplementary file 1 [file vbag220_supplementary_data.zip › tidyexposomics_supplemental_file_1.docx]

**tidyexposomics: integrated exposure-omics analysis powered by tidy principles**

**Supplemental File 1: Methods used in tidyexposomics**

Jason Laird ^1,2^ , Thomas Hartung ^1,2,3,4^, Fenna C. M. Sillé ^1,2^*^§^, Alexandra Maertens ^1,2^*^§^

^1^ Department of Environmental Health and Engineering, Bloomberg School of Public Health, Johns Hopkins University, MD, USA

^2^ Center for Alternatives to Animal Testing (CAAT), Bloomberg School of Public Health and Whiting School of Engineering, Johns Hopkins University, MD, USA

^3^ CAAT-Europe, University of Konstanz, Konstanz, Germany

^4^ Doerenkamp-Zbinden Chair for Evidence-based Toxicology, Department of Environmental Health and Engineering, Johns Hopkins University, Baltimore MD

* These authors contributed equally to this study.

^§^ Corresponding Authors: Fenna C. M. Sillé, PhD, MS and Alexandra Maertens, PhD

**1. Overview of tidyexposomics	2**

**2. Data Input and Syntax	3**

2.1. Syntax	3

2.2. Data Input	3

2.2.1. Ontology Mapping	5

**3. Quality Control and Preprocessing	5**

3.1. Missing Value and Imputation	5

3.2. Omics Feature Filtering	8

3.3. Principal Component Analysis	8

3.4 Exposure Variable Summary	9

**4. Sample Clustering and Exposure Association Analysis	9**

4.1 Sample Clustering	9

4.2 Correlation	11

4.3 ExWAS	11

4.4 Exposome Scores	12

**5. Differential Abundance and Sensitivity Analysis	13**

5.1. Differential Abundance	13

5.2 Sensitivity Analysis	14

**6. Multiomics Integration	17**

6.1 Multiomics Integration	17

**7. Network Analysis	19**

7.1 Network Creation	19

7.2 Exposure Impact Analysis	19

**8. Functional Enrichment Analysis	20**

8.1. Functional Enrichment Analysis	20

**9. Custom Analysis and Summary Functions	22**

9.1. Custom Analysis	22

9.2. Summary Functions and Exporting Results	23

**10. Availability	24**

**11. References	24**

# **1. Overview of tidyexposomics**

The tidyexposomics Bioconductor R package implements a modular and reproducible workflow for integrating exposome and multi-omics data to investigate relationships between environmental exposures, molecular features, and health outcomes. The development version of our package can be found at the following GitHub repository: <https://github.com/BioNomad/tidyexposomics>. Pre-processed example data, used in the package vignette, from the ISGlobal Exposome Data Challenge 2021 are available at <https://doi.org/10.5281/zenodo.17049350>. We design our package around a tidyverse-compatible interface, with structured syntax and intuitive commands. Data is stored within a MultiAssayExperiment object to accommodate multiple data types. By encouraging ontology annotation of exposure variables we foster interoperable findings that can better be compared across analyses. Data preprocessing is supported for both exposure and omics data. Statistical analyses encompass correlation, Exposome-wide association studies (ExWAS), differential abundance, multiomics integration, network analysis, and functional enrichment.

# **2. Data Input and Syntax**

## **2.1. Syntax**

Functions in tidyexposomics are designed to serve three primary purposes:

1. **Running pipelines**: These functions begin with the prefix, run_*, and are used to run more complex operations including correlation, ExWAS, and omics statistical analyses.
2. **Visualizing results**: These functions begin with the prefix, plot_*, and are typically used to visualize results from the outputs of functions that begin with run_*. For example, the results of run_association() are visualized with plot_association().
3. **Data processing**: These functions begin with tidyverse style verbs such as filter_*, transform_*, extract_*, and pivot_*. Data processing functions are used on more base-level data manipulation operations such as filtering out samples.

## **2.2. Data Input**

To analyze exposure and omics data using tidyexposomics users need the following:

1. **Codebook**: The codebook captures information about meta data and exposure variables. This should at minimum include a column variable, which contains the column names of the meta data and the column category, which groups variables into broad categories. See Section 2.2.1. for information on how to map variables using ontologies. The codebook should be input as an R data.frame() object.
2. **Exposure/meta data**: This is tabular data exposure and meta data measurements for samples included in the analysis. The exposure/meta data should be input as an R data.frame() object.
3. **Omics data**: One or more omics assay matrices are needed to. Assay matrices should have molecular features as row names and samples as column names. Omics assays should be input as a named list of matrices. For example, if a user were to input methylation and gene expression data the user might create the following list: list(gene_expression = gene_exp_matrix, methylation = methyl_matrix).
4. **Molecular feature metadata:** These are annotation tables for the omics matrices provided, again provided as a named list. Feature meta data tables should be named using the same names as their corresponding omics matrices. Using the omics data example above, the matched feature data would be: list(gene_expression = gene_exp_features, methylation = methyl_features.

The inclusion of these data are needed to perform exposure-omics analyses using tidyexposomics. These data are input into the create_exposomicset() function to initiate the MultiAssayExperiment. A full example is provided in Supplementary File 2.

## **2.2.1. Ontology Mapping**

To encourage reproducible analyses across studies we provide users the ability to map their variables to three ontologies: Environment Exposure Ontology (ECTO), Human Phenotype Ontology (HPO), and Chemical Entities of Biological Interest (ChEBI). ECTO can be used to annotate exposure variables, HPO can annotate outcome and phenotype variables, and ChEBI can annotate chemical exposures. Ontology mapping has several benefits including making findings more interoperable and harmonized between studies. We leverage OWL files available through BioPortal, a National Center for Biomedical Ontology resource to map variables to structured ontology terms [(Whetzel *et al.* 2011)](https://paperpile.com/c/QH7NKo/oCYF). Ontology mapping is supported through an R shiny application that can be deployed using the build_ont_annot_app() function. Users upload their codebook, select rows, and search ontology terms using the sidebar. Descriptions of chosen ontology terms are dynamically presented to the user to inform assignment choice. Since ontology terms are structured within hierarchies, related terms can be grouped at varying levels of resolution. To group terms, users first select a set of variables and provide a depth level. Ancestor terms are then gathered for each variable and the first ancestor whose depth equals the depth level is the assigned root term or category. Annotated codebooks can then be downloaded to the user’s machine.

# **3. Quality Control and Preprocessing**

## **3.1. Missing Value and Imputation**

Our package offers several tools for assessing data quality, filtering, and transformation. Missingness can be first assessed using the plot_missing() function either at the summary-level to get the number of variables above a certain missingness threshold or at different data layers (i.e. exposures, gene expression, etc.). Variables with greater than a user-specified threshold can be filtered out using the filter_missing() function. If variables with NA values are retained, values can be imputed using several methods: median (naniar), mean (naniar), k-nearest neighbors (impute), multivariate imputation by chained equations (mice), missForest (missForest), and the limit of detection divided by the square root of 2 [(van Buuren and Groothuis-Oudshoorn 2011; Stekhoven and Bühlmann 2012; Tierney and Cook 2023; Hastie *et al.* 2025)](https://paperpile.com/c/QH7NKo/V8I6+Dp0f+yPhV+DvBC). The last option mentioned is especially useful for imputing chemical exposures with a lower limit of detection [(CDC 2025)](https://paperpile.com/c/QH7NKo/dOMQ). After imputation, exposure variables are associated with each other and with omics features later on in the tidyexposomics pipeline. Association analysis can depend on data normality, which can be assessed with the run_normality_check() function, that applies the Shapiro-Wilk test. Normality can be improved by transforming exposure variables (with the transform_exposure() function) using the following methods, where $x$ is the variable to be transformed:

- $log_{2}(x)$
- $x^{1/3}{}$
- $\sqrt{x}$
- $Box-Cox(x)$

Box-cox transformations are determined by calculating a lambda value using the MASS package and a wrapper function described by Hernandez-Ferrer et al. 2019 [(Carles Hernandez-Ferrer, Gregory A Wellenius, Ibon Tamayo, Xavier Basagaña, Jordi Sunyer, Martine Vrijheid, Juan R Gonzalez 2019; Support Functions and Datasets for Venables and Ripley’s MASS [R package MASS version 7.3-65] 2025)](https://paperpile.com/c/QH7NKo/Z6hN+zqkE). Variables are then raised to the power of that lambda value described in Table 1:

### **Table 1. Data transformations applied for different lambda values.**

| **Lambda** | **Transformation** |
| --- | --- |
| -2 | $1/x^{2}$ |
| -1 | $1/x$ |
| -0.5 | $1/\sqrt{x}$ |
| 0 | $ln(x)$ |
| 0.5 | $\sqrt{x}$ |
| 1 | $x$ |
| 2 | $x^{2}$ |

The normality of the variables can be assessed before and after transformations are applied using the plot_normality_summary() function.

## **3.2. Omics Feature Filtering**

High-throughput omics features may include features with low variance or expression depending on the omic type. In tidyexposomics, we offer omic feature filtering based on:

- **Variance**: Where a feature must have a user-defined variance threshold to pass filtering. We recommend using this for omics data that are continuously distributed, such as log-transformed values, M-values, or mass-spectrometry derived intensities.
- **Expression:** This filter is primarily used for gene expression data where an omics feature must pass a count threshold for a certain proportion of the observations. These values are again set by the user.

## **3.3. Principal Component Analysis**

To interrogate the sample and feature space, we offer principal component analysis (PCA) through the run_pca() function. Sample and feature data are scaled and centered before performing dimensionality reduction. Sample outlier detection is supported by calculating the Mahalanobis distance, using the first two principal components, squaring the distance and comparing this to a chi-square threshold with 2 degrees of freedom at a p-value below 0.01 [(Coombes)](https://paperpile.com/c/QH7NKo/8LN7). Sample outliers can be removed if desired using the filter_sample_outliers() function.

## **3.4 Exposure Variable Summary**

For higher level investigation of exposure variables, summary statistics can be calculated using the run_summarize_exposures() function to calculate: number of values, number of NA values, minimum, maximum, range, median, mean, standard error of the mean, lower and upper bound of the 95% confidence interval, variance, standard deviation, and coefficient of variation. Exposure distributions can be visualized with the plot_exposures()function.

# **4. Sample Clustering and Exposure Association Analysis**

## **4.1 Sample Clustering**

Hierarchical clustering is offered in tidyexposomics to cluster samples based on exposure variables in the MultiAssayExperiment object. The euclidean distance can be used with number data and the gower distance can be used for mixed data types (cluster) [(“Finding Groups in Data”: Cluster Analysis Extended Rousseeuw et al. [R package cluster version 2.1.8.1] 2025)](https://paperpile.com/c/QH7NKo/FdOf). To determine the number of clusters, users can either specify their own number of desired clusters or the number of clusters is estimated from the distance matrix using one of the following:

- **DIvisive ANAlysis Clustering (DIANA):** works by splitting the group with the most spread, where splits are made by starting new clusters with the observation with the greatest dissimilarity and reassigning others to the new cluster based on how similar observations are to it (cluster) [(Divisive Analysis (Program DIANA) 2008, “Finding Groups in Data”: Cluster Analysis Extended Rousseeuw et al. [R package cluster version 2.1.8.1] 2025)](https://paperpile.com/c/QH7NKo/lxzT+FdOf).
- **Gap statistic:** determines the optimal number of clusters by calculating the gap statistic (cluster) [(“Finding Groups in Data”: Cluster Analysis Extended Rousseeuw et al. [R package cluster version 2.1.8.1] 2025)](https://paperpile.com/c/QH7NKo/FdOf).
- **Elbow method**: which finds the inflection point in the within-cluster sum of squares curve (factoextra) [(“Finding Groups in Data”: Cluster Analysis Extended Rousseeuw et al. [R package cluster version 2.1.8.1] 2025)](https://paperpile.com/c/QH7NKo/FdOf).
- **Dynamic tree cut**: works by adaptively cutting branches based on the shape of the branch rather than branch height (dynamicTreeCut) [(Langfelder, Zhang and Horvath 2008)](https://paperpile.com/c/QH7NKo/oV87).
- **Density-based**: identifies center points or points with high local density and assigns other observations based on their proximity to these center points (densityClust) [(Clustering by Fast Search and Find of Density Peaks [R package densityClust version 0.3.3] 2024)](https://paperpile.com/c/QH7NKo/s9Kq).

Cluster assignments will be stored in both the metadata of the MultiAssayExperiment object and as a column in the colData. Adding sample group information to the colData makes assignments accessible for other analysis (association, differential abundance, etc.). Sample groups can be visualized as a heatmap using the plot_sample_clusters() function.

## **4.2 Correlation**

Correlations between exposures and omics are supported through the run_correlation() function. Correlation can be determined between:

- Omics features
- Exposures
- Exposures and principal components
- Exposures and omics features
- Exposures and latent factors (latent factors are described in Section 6.1.)

Either Pearson or Spearman correlation can be calculated using the [Hmisc package](https://cran.r-project.org/web/packages/Hmisc/index.html) [(Harrell 2025)](https://paperpile.com/c/QH7NKo/tg51). Correlations can be filtered to include correlations above a certain absolute correlation value or those with a p-value or False-Discovery-Rate (FDR) adjusted p-value below a user specified threshold. Variable relationships can be visualized with the plot_circos_correlation() function.

## **4.3 ExWAS**

We model associations using generalized linear models (GLMs) with a user-specified outcome and features drawn from either exposures, omics features, or latent factors. Before inclusion in the model, numeric exposure data and omics features are scaled. The option is provided to log_2_ transform omics features before scaling. Models with continuous and binary outcomes are supported, with and without the inclusion of covariates. We compute R^2^ and adjusted R^2^ values described in Table 2 [(FAQ: What are pseudo R-squareds?)](https://paperpile.com/c/QH7NKo/qOr7):

### **Table 2. Formulas for** $R^{2}$ **and** ${Adjusted R}^{2}$ **of GLM models.**

| $Outcome$ | $R^{2}$ | ${Adjusted R}^{2}$ |
| --- | --- | --- |
| $Gaussian$ | $1 -deviance/null deviance$ | $1 -(n - 1)/(n - p) * (1 - R^{2})$ |
| $Binomial$ | $1 -l_{full}/l_{null}$ | $1 -(l_{full} - p)/{(l}_{null})$ |

Where $p$ is the number of parameters in the model including the intercept, $n$ is the number of samples, $l_{full}$ and $l_{null}$ are the log-likelihoods of the fitted and null models. P-values are adjusted for multiple-hypothesis-testing using the FDR method. Association results can be plotted using the plot_association() function.

## **4.4 Exposome Scores**

Exposures often occur as mixtures and aggregating exposure values can help better predict risk [(Liu *et al.* 2025)](https://paperpile.com/c/QH7NKo/hT4d). In our package we offer the following methods to aggregate exposure values using the run_exposome_score() function:

- **Median**: Where the final score value is the median of the selected exposure variables.
- **Mean**: Where the final score value is the mean of the selected exposure variables.
- **Sum**: Where the final score value is the sum of the selected exposure variables.
- **PCA**: Where the score represents the first principal component of the selected exposure variables.
- **Quantile**: Where each exposure was converted to decile ranks (1 - 10), and summed across exposures to yield a cumulative quantile-based score.
- **Item response theory**: Where exposures again are converted to deciles, and a graded response model was fit with the mirt package, where an estimate is calculated to best represent the overall exposure profile [(Chalmers 2025)](https://paperpile.com/c/QH7NKo/Z0TW).
- **Variance:** Where the final score value is the variance of the selected exposure variables.

Users may select sets of exposure variables and can aggregate them using the methods described above. The resulting score is stored in the MultiAssayExperiment object and as a column in the colData of the MultiAssayExperiment object.

# **5. Differential Abundance and Sensitivity Analysis**

## **5.1. Differential Abundance**

Differential abundance testing across all assays is supported using the run_differential_abundance() function. Users can specify a formula, contrasts to define specific comparisons, and the differential abundance method. Differential abundance testing is largely supported through the tidybulk package where the following methods are supported: edgeR_lrt, edgeR_quasi_lrt, DESeq2, and limma_voom [(Mangiola *et al.* 2021)](https://paperpile.com/c/QH7NKo/ldz2). However, given that these methods are primarily meant for RNA-seq data we create a wrapper for limma_trend to allow for differential abundance testing of continuous measurements, such as log_2_ transformed data and mass-spectrometry derived intensities [(Ritchie *et al.* 2015)](https://paperpile.com/c/QH7NKo/LptO).

## **5.2 Sensitivity Analysis**

To assess the robustness of differentially abundant features, we implement a grid-based approach. Starting from a base model ($\sim condition + covariates$), covariates are removed, and these model variates are crossed with different differential abundance and scaling methods. The number of differential abundance and scaling methods is set by the user. Differential abundance methods include the options described in Section 5.1, and scaling options include options available in tidybulk: none, trimmed mean of M values (TMM), and quantile [(Mangiola *et al.* 2021)](https://paperpile.com/c/QH7NKo/ldz2). Additionally, we offer the user the ability to bootstrap resample their samples with replacement, where the grid approach described above would be run for every bootstrap run. Feature stability can be assessed with the following methods described in Table 3.

### **Table 3. Methods used to assess the stability of differentially abundant features.**

|  | **Description** | **Formula** |
| --- | --- | --- |
| Presence rate | How often the feature was labeled a differentially abundant feature. | $1/runs * \sum_{r \in runs} p_{r} < \alpha$ |
| Effect Consistency | Penalizes high variability relative to the average effect size. | $cv_{logFC} =sd(logFC)/(mean(\vert logFC\vert)$  $1/ (1 + cv_{logFC})$ |
| Stability Score | Hybrid score which is the product of the presence rate and effect consistency. | $presence rate * effect consistency$ |
| Mean Log(P-value) | Mean of the p-values across runs. Where $\epsilon$ is meant to avoid taking the $log(0).$ | $\epsilon= 1e-10$  $1/runs *\sum_{r \in runs} p_{r} +\epsilon$ |
| Log(P-value) Weighted Score | Mean of the p-values across runs multiplied by the effect consistency. | $mean log(p-value) * effect consistency$ |
| Standard Deviation of Log Fold Change | Standard deviation of the log fold change to measure the variability of log fold change estimates. | $sd(logFC)$ |
| Interquartile Range (IQR) of Log Fold Change | The interquartile range of the log fold changes values to measure the spread of values. | $IQR(logFC)$ |
| Coefficient of Variation (CV) of Log Fold Change | Coefficient of variation of the log fold change which summarizes the variance in log fold change estimates. | $sd(logFC)/(mean(\vert logFC\vert)$ |
| Sign Flip Frequency | How often the sign of the log fold change flips between positive and negative. | $1/runs*\sum_{r \in runs} \{sign(logFC_{r})\neq sign(mean(logFC))\}$ |
| Where $runs$ is the number of different conditions tested (method x scaling x model variant x bootstrap run), $p_{r}$ is the p-value for run within $runs$, $\alpha$ is the significance level, $cv_{logFC}$ is the coefficient of variation of the log fold change, $\epsilon$ is a pseudo-count added to avoid taking the log of 0. | | |

The user dictates what p-value and log fold change thresholds determine if a feature is differentially abundant. A threshold for the chosen feature stability metric (default is stability score using the upper quantile (default is 0.9)) or set by the user. The results of the sensitivity analysis can be plotted using the plot_sensitivity_summary() function.

# **6. Multiomics Integration**

## **6.1 Multiomics Integration**

Multiomics integration methods are supported in tidyexposomics using the run_multiomics_integration() function. Here users specify the number of latent factors to generate, an outcome variable (if performing supervised integration), and the multiomics integration method. The following methods are supported:

- **Multi-Omics Factor Analysis (MOFA):** An unsupervised factor model implemented in the MOFA2 packages where a set of latent factors are estimated that explain variation across multiple omics assays [(Argelaguet *et al.* 2018)](https://paperpile.com/c/QH7NKo/Z8ne).
- **Multiple Co-Inertia Analysis (MCIA)**: MCIA extends co-inertia analysis to multiple assays where each block is projected into common factor space by maximizing the covariance between datasets. MICA is implemented through the nipalsMCIA package [(Mattessich *et al.* 2024)](https://paperpile.com/c/QH7NKo/nBZZ).
- **Regularized Generalized Canonical Correlation Analysis (RGCCA)**: RGCCA generalizes canonical correlation analysis to more than two blocks by using regularization parameters to balance variance explained and correlation maximization. RGCCA is implemented through the RGCCA package [(Girka *et al.*)](https://paperpile.com/c/QH7NKo/A9i3).
- **Data Integration Analysis for Biomarker discovery using Latent cOmponents (DIABLO)**: DIABLO is a supervised integration method implemented in the mixOmics package where a block sparse Partial Least Squares Discriminant Analysis (PLS-DA) model is fit that maximizes correlation between omics components while predicting some categorical outcome [(Rohart *et al.* 2017)](https://paperpile.com/c/QH7NKo/1I9j).

Omics features can have varying contributions to latent factors. In order to discover features that are associated with an outcome in multiple experiments, the extract_top_factor_features() function identifies features whose absolute loading value is above a user-set percentile. Top factor features can be plotted for specific factors using the plot_top_factor_features() function. Since similar features may contribute to multiple latent factors, the run_factor_overlap() function identifies which features appear more than once in the top factor features determined by extract_top_factor_features().

# **7. Network Analysis**

## **7.1 Network Creation**

We construct undirected graphs using the run_create_network() function which leverages the igraph R package, using the correlation results described in Section 4.2 [(Csárdi and Nepusz 2006)](https://paperpile.com/c/QH7NKo/04Vd). Nodes represent the variables correlated in the correlation table and the edges represent the correlation between them. Correlation and p-value cutoffs can be applied to filter the correlation table before network creation. Networks can be plotted using the plot_network() function, which sizes nodes by their centrality in the network. The following network statistics may also be included in the plot: number of nodes, number of edges, the number of disconnected subgraphs, the longest shortest path between any two nodes, the average shortest path between any two nodes, and the Louvian modularity of the graph indicating subgraph presence.

## **7.2 Exposure Impact Analysis**

Many -omics features can be correlated with exposures. In order to get a higher level view of the exposure impact on the molecular features using a network perspective we provide an exposure-impact analysis (run_exposure_impact()). This analysis relies on two networks, a bipartite network between exposures and omics features and an omics-feature-only network. This is done by first building a separate graph containing only the features. The network drawn *de novo* from the -omics data identifies critical molecular features via network topology, which are summarized by exposure. For each feature in this network, we compute four measures of how "central" or well-connected it is using the tidygraph package [(Pedersen 2024)](https://paperpile.com/c/QH7NKo/rCsJ):

- **Degree:** Number of direct connections.
- **Eigenvector:** Connections to other well-connected features.
- **Closeness:** How easily a feature can reach others.
- **Betweenness:** How often a feature lies on paths between others.

For each exposure, we average the centrality scores of all its associated features. This "impact score" reflects how central an exposure's features are within the network.

The plot_exposure_impact() function creates a heatmap showing the scaled average centrality scores, allowing easy comparison of exposure importance.

# **8. Functional Enrichment Analysis**

## **8.1. Functional Enrichment Analysis**

Functional enrichment analysis can be performed using the run_enrichment() function, which uses the fenr package [(Gierlinski 2025)](https://paperpile.com/c/QH7NKo/nDHD). Here we support Gene Ontology (GO), Kyoto Encyclopedia of Genes and Genomes (KEGG), and Reactome enrichment analysis. Enrichment is performed on a per assay basis, where the universe of possible molecular features is set by the rowData() of each assay. Differentially abundant features, factor features, features in correlation results, and user-specified features may all be used as inputs. If features cannot be mapped, an empty result is returned for this assay (e.g. if using a method that requires a gene identifier for metabolomics data). Results can be filtered for significance, gene set size, and will optionally cluster terms by Jaccard overlap of their terms:

$J(A,B) = |A \cap B| / |A\cup B|$

Where A and B are the two sets of features. Pairwise overlap code was based on a function written by Blischak 2020 [(Blischak 2020)](https://paperpile.com/c/QH7NKo/vkmu). We then compute a Jaccard matrix among enriched term, covert to a dissimilarity matrix ($1 - J$), and cluster using the algorithms described in Section 4.1. (default is diana clustering).

Enrichment results can be visualized using the plot_enrichment() function using the following plots:

- **Dotplot**: term groups are either ranked by a group score ($mean \{-log_{10}(p_{adj}) x n_{with selected}\}$). Where $p_{adj}$ is the FDR-adjusted p-value of the term and $n_{with selected}$ is the number of features that overlap with the larger set. Users have the option to bypass this and choose which term groups to plot. Individual points are sized by the number of features driving that term group and colored by the $-log_{10}(p_{adj})$.
- **Heatmap**: The rows of the heatmap are the enriched terms and the columns are the molecular features driving the enrichment. Tiles can be optionally colored by the log2 fold change from the differential abundance testing.
- **Term-term overlap network**: Here an undirected graph is constructed where nodes are terms and edges are the Jaccard overlap between those terms that are above a user-specified threshold. Nodes are drawn as a pie indicating which omics assays contributed to the term. The top degree nodes within Louvain communities can also be plotted.
- **Gene-term bipartite graph**: A bipartite graph is created between term nodes and the features associated with them. Term nodes are again rendered as pies by omics assay and gene nodes can be colored by log_2_ fold change.
- **Summary:** A summary plot may also be generated which displays a barplot of enriched terms per omics assay, per exposure category, a ridgeplot of the $-log_{10}(p_{adj})$ distributions across term groups, and a venn diagram of the term overlaps across omics assays.

# **9. Custom Analysis and Summary Functions**

## **9.1. Custom Analysis**

To facilitate other tidy-based workflows, we provide the following three functions inspired by the tidybulk package [(Mangiola *et al.* 2021)](https://paperpile.com/c/QH7NKo/ldz2):

- pivot_sample() : Where the colData() from the MultiAssayExperiment object are extracted as a tibble with the samples under the .sample column.
- pivot_feature() : This function iterates over all assays within the MultiAssayExperiment object and extracts feature-level metadata data, concatenates into a single tibble, adds the .exp_name to indicate the assay of origin, and .feature which contains the molecular feature.
- pivot_exp() : Extracts a specified assay from a MultiAssayExperiment object and can filter the object for specific features. The resulting SummarizedExperiment is converted to a tibble including all the colData(), rowData(), and assay data. The resulting tibble contains the columns: .sample to indicate samples, .exp_name to indicate the assay of origin, and .feature which contains the molecular feature in a particular assay.

## **9.2. Summary Functions and Exporting Results**

The pivot_* functions provided above are useful for extracting data from the assay, colData, and rowData of the MultiAssayExperiment object. However, to extract the results generated in the tidyexposomics pipeline, we provide the extract_results(). The codebook, quality control, correlation, association, differential analysis, multiomics integration, network and enrichment results can all be extracted in this fashion. This method extracts the results as R list objects for further interrogation within the R environment. Reportable tabular data can be directly exported to an excel spreadsheet using the extract_results_excel() function. However, the extract_results_excel() function does not access the codebook and quality control results. However this function does generate an exposure summary table, described in Section 3.4., and a table of all of the pipeline steps taken to foster transparent reporting. Users may optionally print either a diagram or these pipeline steps directly to their R console using the run_pipeline_summary() function.

# **10. Availability**

tidyexposomics is made publicly available:

- **GitHub repository:** <https://github.com/BioNomad/tidyexposomics>
- **Pre-processed example data**: <https://doi.org/10.5281/zenodo.17049350>

# **11. References**

[Argelaguet R, Velten B, Arnol D *et al.* Multi-Omics Factor Analysis-a framework for unsupervised integration of multi-omics data sets. *Mol Syst Biol* 2018;**14**, DOI:](http://paperpile.com/b/QH7NKo/Z8ne) [10.15252/msb.20178124](http://dx.doi.org/10.15252/msb.20178124)[.](http://paperpile.com/b/QH7NKo/Z8ne)

[Blischak J. How to efficiently calculate pairwise overlaps of many sets. *John Blischak’s blog* 2020.](http://paperpile.com/b/QH7NKo/vkmu)

[van Buuren S, Groothuis-Oudshoorn K. mice: Multivariate Imputation by Chained Equations in*R*. *J Stat Softw* 2011;**45**, DOI:](http://paperpile.com/b/QH7NKo/yPhV) [10.18637/jss.v045.i03](http://dx.doi.org/10.18637/jss.v045.i03)[.](http://paperpile.com/b/QH7NKo/yPhV)

[Carles Hernandez-Ferrer, Gregory A Wellenius, Ibon Tamayo, Xavier Basagaña, Jordi Sunyer, Martine Vrijheid, Juan R Gonzalez. Comprehensive study of the exposome and omic data using rexposome Bioconductor Packages. *Bioinformatics* 2019;**35**:5344–5.](http://paperpile.com/b/QH7NKo/zqkE)

[CDC. Limit of Detection. *National Report on Human Exposure to Environmental Chemicals* 2025.](http://paperpile.com/b/QH7NKo/dOMQ)

[Chalmers P. Multidimensional Item Response Theory [R package mirt version 1.44.0]. *Comprehensive R Archive Network (CRAN)* 2025.](http://paperpile.com/b/QH7NKo/Z0TW)

[Clustering by Fast Search and Find of Density Peaks [R package densityClust version 0.3.3]. *Comprehensive R Archive Network (CRAN)* 2024.](http://paperpile.com/b/QH7NKo/s9Kq)

[Coombes K. PCA, Mahalanobis Distance, and Outliers.](http://paperpile.com/b/QH7NKo/8LN7)

[Csárdi G, Nepusz T. The igraph software package for complex network research. *InterJournal, Complex Systems* 2006.](http://paperpile.com/b/QH7NKo/04Vd)

[Divisive Analysis (Program DIANA). *Finding Groups in Data*. Hoboken, NJ, USA: John Wiley & Sons, Inc., 2008, 253–79.](http://paperpile.com/b/QH7NKo/lxzT)

[FAQ: What are pseudo R-squareds?](http://paperpile.com/b/QH7NKo/qOr7)

[“Finding Groups in Data”: Cluster Analysis Extended Rousseeuw et al. [R package cluster version 2.1.8.1]. *Comprehensive R Archive Network (CRAN)* 2025.](http://paperpile.com/b/QH7NKo/FdOf)

[Gierlinski M. fenr: Fast functional enrichment for interactive applications. 2025, DOI:](http://paperpile.com/b/QH7NKo/nDHD) [10.18129/B9.bioc.fenr](http://dx.doi.org/10.18129/B9.bioc.fenr)[.](http://paperpile.com/b/QH7NKo/nDHD)

[Girka F, Camenen E, Peltier C *et al.* *RGCCA: Regularized and Sparse Generalized Canonical Correlation Analysis for Multiblock Data*.](http://paperpile.com/b/QH7NKo/A9i3)

[Harrell FE Jr. Harrell Miscellaneous [R package Hmisc version 5.2-3]. *Comprehensive R Archive Network (CRAN)* 2025.](http://paperpile.com/b/QH7NKo/tg51)

[Hastie T, Tibshirani R, Narasimhan B *et al.* impute: impute: Imputation for microarray data. 2025, DOI:](http://paperpile.com/b/QH7NKo/V8I6) [10.18129/B9.b](http://dx.doi.org/10.18129/B9.b)[.](http://paperpile.com/b/QH7NKo/V8I6)

[Langfelder P, Zhang B, Horvath S. Defining clusters from a hierarchical cluster tree: the Dynamic Tree Cut package for R. *Bioinformatics* 2008;**24**, DOI:](http://paperpile.com/b/QH7NKo/oV87) [10.1093/bioinformatics/btm563](http://dx.doi.org/10.1093/bioinformatics/btm563)[.](http://paperpile.com/b/QH7NKo/oV87)

[Liu SH, Manz KE, Buckley JP *et al.* Exposome burden scores to summarize environmental chemical mixtures: Creating a fair and common scale for cross-study harmonization, report-back and precision environmental health. *Curr Environ Health Rep* 2025;**12**, DOI:](http://paperpile.com/b/QH7NKo/hT4d) [10.1007/s40572-024-00467-2](http://dx.doi.org/10.1007/s40572-024-00467-2)[.](http://paperpile.com/b/QH7NKo/hT4d)

[Mangiola S, Molania R, Dong R *et al.* tidybulk: an R tidy framework for modular transcriptomic data analysis. *Genome Biol* 2021;**22**, DOI:](http://paperpile.com/b/QH7NKo/ldz2) [10.1186/s13059-020-02233-7](http://dx.doi.org/10.1186/s13059-020-02233-7)[.](http://paperpile.com/b/QH7NKo/ldz2)

[Mattessich M, Reyna J, Aron E *et al.* nipalsMCIA: flexible multi-block dimensionality reduction in R via nonlinear iterative partial least squares. *Bioinformatics* 2024;**41**, DOI:](http://paperpile.com/b/QH7NKo/nBZZ) [10.1093/bioinformatics/btaf015](http://dx.doi.org/10.1093/bioinformatics/btaf015)[.](http://paperpile.com/b/QH7NKo/nBZZ)

[Pedersen TL. A Tidy API for Graph Manipulation [R package tidygraph version 1.3.1]. *Comprehensive R Archive Network (CRAN)* 2024.](http://paperpile.com/b/QH7NKo/rCsJ)

[Ritchie ME, Phipson B, Wu D *et al.* limma powers differential expression analyses for RNA-sequencing and microarray studies. *Nucleic Acids Res* 2015;**43**, DOI:](http://paperpile.com/b/QH7NKo/LptO) [10.1093/nar/gkv007](http://dx.doi.org/10.1093/nar/gkv007)[.](http://paperpile.com/b/QH7NKo/LptO)

[Rohart F, Gautier B, Singh A *et al.* mixOmics: An R package for ’omics feature selection and multiple data integration. *PLoS Comput Biol* 2017;**13**, DOI:](http://paperpile.com/b/QH7NKo/1I9j) [10.1371/journal.pcbi.1005752](http://dx.doi.org/10.1371/journal.pcbi.1005752)[.](http://paperpile.com/b/QH7NKo/1I9j)

[Stekhoven DJ, Bühlmann P. MissForest--non-parametric missing value imputation for mixed-type data. *Bioinformatics* 2012;**28**, DOI:](http://paperpile.com/b/QH7NKo/Dp0f) [10.1093/bioinformatics/btr597](http://dx.doi.org/10.1093/bioinformatics/btr597)[.](http://paperpile.com/b/QH7NKo/Dp0f)

[Support Functions and Datasets for Venables and Ripley’s MASS [R package MASS version 7.3-65]. *Comprehensive R Archive Network (CRAN)* 2025.](http://paperpile.com/b/QH7NKo/Z6hN)

[Tierney N, Cook D. Expanding tidy data principles to facilitate missing data exploration, visualization and assessment of imputations. *J Stat Softw* 2023;**105**, DOI:](http://paperpile.com/b/QH7NKo/DvBC) [10.18637/jss.v105.i07](http://dx.doi.org/10.18637/jss.v105.i07)[.](http://paperpile.com/b/QH7NKo/DvBC)

[Whetzel PL, Noy NF, Shah NH *et al.* BioPortal: enhanced functionality via new Web services from the National Center for Biomedical Ontology to access and use ontologies in software applications. *Nucleic Acids Res* 2011;**39**, DOI:](http://paperpile.com/b/QH7NKo/oCYF) [10.1093/nar/gkr469](http://dx.doi.org/10.1093/nar/gkr469)[.](http://paperpile.com/b/QH7NKo/oCYF)
