# Supplementary material for: tidyexposomics: integrated exposure-omics analysis powered by tidy principles
Source: Bioinform Adv. 2026 Aug 8;6(1):vbag220. doi: 10.1093/bioadv/vbag220 (PMC13532756; doi:10.1093/bioadv/vbag220)
Supplement: Supplementary file 1 [file vbag220_supplementary_data.zip › benchmark_supplementary.pdf]

# tidyexposomics: Integrated exposure-omics analysis powered by tidy principles

*Supplemental File 3: tidyexposomics performance*

**Jason Laird[1,2], Thomas Hartung[1,2,3,4], Fenna C. M. Sillé[1,2*§], Alexandra Maertens[1,2*§]**

[1] Department of Environmental Health and Engineering, Bloomberg School of Public Health, Johns Hopkins University, MD, USA

[2] Center for Alternatives to Animal Testing (CAAT), Bloomberg School of Public Health and Whiting School of Engineering, Johns Hopkins University, MD, USA

[3] CAAT-Europe, University of Konstanz, Konstanz, Germany

[4] Doerenkamp-Zbinden Chair for Evidence-based Toxicology, Department of Environmental Health and Engineering, Johns Hopkins University, Baltimore, MD, USA

* These authors contributed equally to this study.

§ Corresponding Authors: Fenna C. M. Sillé, PhD, MS and Alexandra Maertens, PhD

**2026-07-03**

# Contents

1 Overview . . . 3
2 Setup . . . 3
2.1 Libraries . . . 3
2.2 Load Data . . . 3
2.3 Sample ID Alignment . . . 3
3 Data Preparation . . . 3
3.1 Define Exposure Variables . . . 3
3.2 Subset Metadata . . . 4
3.3 Find Common Samples . . . 4
3.4 Prepare Gene Expression Matrix . . . 4
4 Benchmark Design . . . 4
4.1 Scaling Parameters . . . 4
4.2 Data Preparation Helper . . . 5
4.3 Benchmark Functions . . . 6
5 Run Benchmark . . . 7
6 Results . . . 8

6.1 Memory Helper . . . . . . . . 8
6.2 Runtime Plots . . . . . . . . 9
6.3 Memory Plots . . . . . . . . 11
6.4 Combined Figure . . . . . . . . 12

7 Session Info . . . . . . . . 14

# 1 Overview

This document describes the methodology and results for the scalability benchmark comparing `tidyexposomics` and `rexposome`/`omicRexposome` for exposure-omics association testing.

# 2 Setup

## 2.1 Libraries

```
library(tidyverse)
library(scales)
library(tidyexposomics)
library(rexposome)
library(omicRexposome)
library(bench)
library(Biobase)
library(MultiDataSet)
library(patchwork)
```

## 2.2 Load Data

```
omics_list  <- download_dataset("omics_list")
fdata       <- download_dataset("fdata")
meta        <- download_dataset("meta")
annotated_cb <- download_dataset("annotated_cb")
load("~/jhu/projects/useful_functions/genexpr.Rdata")
```

## 2.3 Sample ID Alignment

```
meta_to_eset_id <- function(meta_id) gsub("^s", "", meta_id)
eset_to_meta_id <- function(eset_id) paste0("s", eset_id)
```

# 3 Data Preparation

## 3.1 Define Exposure Variables

```
exp_vars <- annotated_cb |>
  filter(
    var_type == "numeric",
    category %in% c("aerosol", "main group molecular entity", "polyatomic entity")
  ) |>
  pull(variable)
```

```
annotated_cb <- annotated_cb |>
  filter(variable %in% exp_vars)
```

## 3.2 Subset Metadata

```
meta_subset <- meta |>
  filter(FAS_cat_None == "Low") |>
  dplyr::select(
    hs_asthma,
    e3_sex_None,
    hs_child_age_None,
    all_of(exp_vars)
  )
```

## 3.3 Find Common Samples

```
meta_samples         <- rownames(meta_subset)
eset_samples         <- sampleNames(genexpr)
common_samples_eset  <- intersect(meta_to_eset_id(meta_samples), eset_samples)
common_samples_meta  <- eset_to_meta_id(common_samples_eset)

meta_full   <- meta_subset[common_samples_meta, ]
genexpr_full <- genexpr[, common_samples_eset]
```

## 3.4 Prepare Gene Expression Matrix

Features are ordered by variance so that the top most variable features are selected consistently across benchmark conditions.

```
gexp_matrix_full <- exprs(genexpr_full)
colnames(gexp_matrix_full) <- eset_to_meta_id(colnames(gexp_matrix_full))

gexp_var     <- apply(gexp_matrix_full, 1, var, na.rm = TRUE)
feature_order <- names(sort(gexp_var, decreasing = TRUE))
```

# 4 Benchmark Design

## 4.1 Scaling Parameters

```
n_samples_max  <- length(common_samples_meta)
n_features_max <- length(feature_order)

n_samples_vec <- c(10, 25, 50, 75, 100)
n_samples_vec <- n_samples_vec[n_samples_vec <= n_samples_max]
if (!n_samples_max %in% n_samples_vec) {
```

```
  n_samples_vec <- c(n_samples_vec, n_samples_max)
}

n_features_vec <- c(100, 500, 1000, 2500, 5000)
n_features_vec <- n_features_vec[n_features_vec <= n_features_max]
```

## 4.2 Data Preparation Helper

This function subsets data to a given number of samples and features, returning separate objects formatted for each package.

```
prepare_scaled_data <- function(n_samples, n_features) {

  sample_ids_meta <- common_samples_meta[1:n_samples]
  sample_ids_eset <- meta_to_eset_id(sample_ids_meta)
  top_features    <- feature_order[1:n_features]

  meta_sub <- meta_full[sample_ids_meta, ]
  gexp_sub <- gexp_matrix_full[top_features, sample_ids_meta]

  fdata_sub <- if (nrow(fData(genexpr_full)) > 0) {
    fData(genexpr_full)[top_features, , drop = FALSE]
  } else {
    data.frame(row.names = top_features, feature_id = top_features)
  }

  exposures_df <- meta_sub |>
    rownames_to_column("idnum") |>
    mutate(idnum = meta_to_eset_id(idnum)) |>
    dplyr::select(idnum, all_of(exp_vars)) |>
    column_to_rownames("idnum") |>
    as.data.frame()

  phenotypes_df <- meta_sub |>
    rownames_to_column("idnum") |>
    mutate(idnum = meta_to_eset_id(idnum)) |>
    dplyr::select(idnum, hs_asthma, e3_sex_None, hs_child_age_None) |>
    column_to_rownames("idnum") |>
    as.data.frame()

  eset_sub <- genexpr_full[top_features, sample_ids_eset]
  fData(eset_sub)$end        <- fData(eset_sub)$stop
  fData(eset_sub)$chromosome <- fData(eset_sub)$seqname

  list(
    n_samples    = n_samples,
    n_features   = n_features,
    meta_sub     = meta_sub,
    gexp_sub     = gexp_sub,
    fdata_sub    = fdata_sub,
```

```
      exposures_df = exposures_df,
      phenotypes_df = phenotypes_df,
      eset_sub     = eset_sub
    )
  }
```

## 4.3 Benchmark Functions

```
make_bench_tidy <- function(data) {
  function() {
    create_exposomicset(
      codebook = annotated_cb,
      exposure = data$meta_sub,
      omics    = list("Gene Expression" = data$gexp_sub),
      row_data = list("Gene Expression" = data$fdata_sub)
    ) |>
      run_impute_missing(
        exposure_impute_method = "median",
        exposure_cols          = exp_vars
      ) |>
      run_exposure_omics_association(
        exposures  = exp_vars,
        covariates = c("e3_sex_None", "hs_child_age_None")
      )
  }
}

make_bench_rexposome <- function(data) {
  function() {
    description_df <- annotated_cb |>
      dplyr::select(Exposure = variable, Family = category) |>
      column_to_rownames("Exposure") |>
      as.data.frame()

    exp_set <- loadExposome(
      exposures        = data$exposures_df,
      description      = description_df,
      phenotype        = data$phenotypes_df,
      description.famCol = "Family"
    )

    exp_imputed <- imputation(exp_set)

    mds <- createMultiDataSet()
    mds <- add_exp(mds, exp_imputed)
    mds <- add_genexp(mds, data$eset_sub)

    association(
      mds,
```

```
        formula  = ~ e3_sex_None + hs_child_age_None,
        expset   = "exposures",
        omicset  = "expression"
      )
    }
  }
```

# 5 Run Benchmark

```
scalability_results <- expand.grid(
  n_samples  = n_samples_vec,
  n_features = n_features_vec
) |>
  as_tibble() |>
  mutate(
    n_models    = n_features * length(exp_vars),
    tidy_time   = NA_real_,
    rex_time    = NA_real_,
    tidy_mem    = NA_character_,
    rex_mem     = NA_character_,
    tidy_status = NA_character_,
    rex_status  = NA_character_
  )

for (i in seq_len(nrow(scalability_results))) {

  n_samp <- scalability_results$n_samples[i]
  n_feat <- scalability_results$n_features[i]

  data <- prepare_scaled_data(n_samp, n_feat)

  tidy_result <- tryCatch({
    res <- bench::mark(
      make_bench_tidy(data)(),
      iterations = 1,
      check      = FALSE,
      memory     = TRUE
    )
    list(
      time   = as.numeric(res$median),
      mem    = as.character(res$mem_alloc),
      status = "success"
    )
  }, error = function(e) {
    list(time = NA, mem = NA, status = paste("error:", e$message))
  })

  rex_result <- tryCatch({
    res <- bench::mark(
```

```r
        make_bench_rexposome(data)(),
        iterations = 1,
        check      = FALSE,
        memory     = TRUE
      )
      list(
        time   = as.numeric(res$median),
        mem    = as.character(res$mem_alloc),
        status = "success"
      )
    }, error = function(e) {
      list(time = NA, mem = NA, status = paste("error:", e$message))
    })

    scalability_results$tidy_time[i]   <- tidy_result$time
    scalability_results$rex_time[i]    <- rex_result$time
    scalability_results$tidy_mem[i]    <- tidy_result$mem
    scalability_results$rex_mem[i]     <- rex_result$mem
    scalability_results$tidy_status[i] <- tidy_result$status
    scalability_results$rex_status[i]  <- rex_result$status
}
```

# 6 Results

## 6.1 Memory Helper

```r
parse_memory <- function(mem_str) {
  value <- as.numeric(gsub("[^0-9.]", "", mem_str))
  unit  <- gsub("[0-9.]", "", mem_str)
  case_when(
    unit == "KB" ~ value / 1024,
    unit == "MB" ~ value,
    unit == "GB" ~ value * 1024,
    TRUE         ~ value
  )
}

memory_results <- scalability_results |>
  filter(tidy_status == "success", rex_status == "success") |>
  mutate(
    tidy_mem_mb = parse_memory(tidy_mem),
    rex_mem_mb  = parse_memory(rex_mem),
    mem_ratio   = tidy_mem_mb / rex_mem_mb
  )
```

## 6.2 Runtime Plots

```
t_v_f <- scalability_results |>
  filter(tidy_status == "success", rex_status == "success") |>
  pivot_longer(
    cols      = c(tidy_time, rex_time),
    names_to  = "package",
    values_to = "time"
  ) |>
  mutate(
    package = recode(package,
      "tidy_time" = "tidyexposomics",
      "rex_time"  = "rexposome"
    ),
    facet_label = paste("n =", n_samples, "samples") |>
      factor(levels = paste("n =", n_samples_vec, "samples"))
  ) |>
  ggplot(aes(x = n_features, y = time, color = package)) +
  geom_line(linewidth = 1) +
  geom_point(size = 3) +
  facet_wrap(~ facet_label, nrow = 1) +
  scale_x_log10(labels = scales::comma) +
  scale_y_log10() +
  labs(
    title = "Scalability by Feature Count",
    x     = "Number of Omics Features",
    y     = expression("Log"[10]*"[Time (s)]"),
    color = "Package"
  ) +
  scale_color_manual(values = c(
    "rexposome"      = "#3A68AE",
    "tidyexposomics" = "#883268"
  )) +
  theme_minimal() +
  theme(legend.position = "bottom")

t_v_f
```

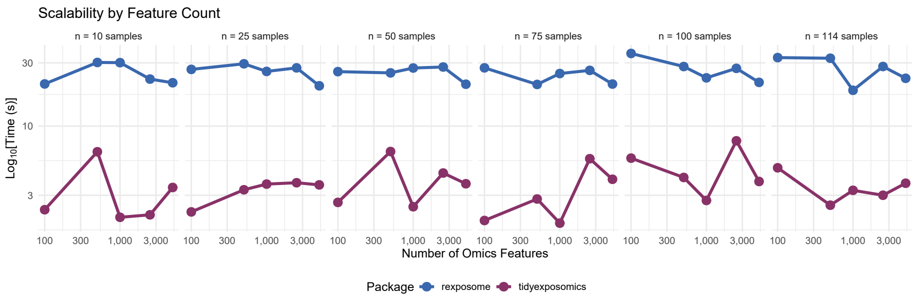


**Figure 1:** Figure 1a. Runtime by omics feature count, faceted by sample size.

```r
t_v_s <- scalability_results |>
  filter(tidy_status == "success", rex_status == "success") |>
  pivot_longer(
    cols      = c(tidy_time, rex_time),
    names_to  = "package",
    values_to = "time"
  ) |>
  mutate(
    package = recode(package,
      "tidy_time" = "tidyexposomics",
      "rex_time"  = "rexposome"
    ),
    facet_label = paste(scales::comma(n_features), "features") |>
      factor(levels = paste(scales::comma(n_features_vec), "features"))
  ) |>
  ggplot(aes(x = n_samples, y = time, color = package)) +
  geom_line(linewidth = 1) +
  geom_point(size = 3) +
  facet_wrap(~ facet_label, nrow = 1) +
  scale_y_log10() +
  labs(
    title = "Scalability by Sample Size",
    x     = "Number of Samples",
    y     = expression("Log"[10]*"[Time (s)]"),
    color = "Package"
  ) +
  scale_color_manual(values = c(
    "rexposome"      = "#3A68AE",
    "tidyexposomics" = "#883268"
  )) +
  theme_minimal() +
  theme(legend.position = "bottom")

t_v_s
```

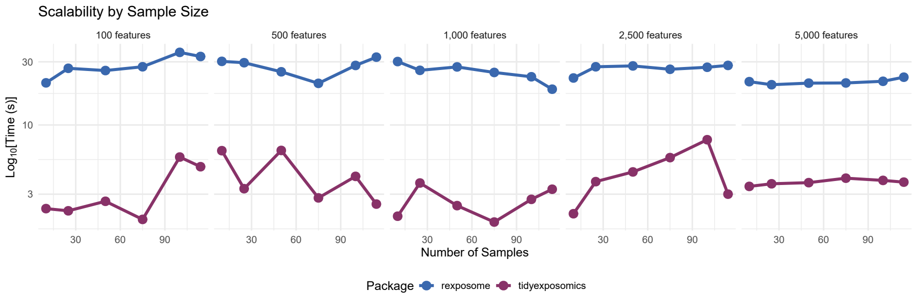


**Figure 2:** Figure 1b. Runtime by sample size, faceted by feature count.

## 6.3 Memory Plots

```
m_v_f <- memory_results |>
  pivot_longer(
    cols      = c(tidy_mem_mb, rex_mem_mb),
    names_to  = "package",
    values_to = "memory_mb"
  ) |>
  mutate(
    package = recode(package,
      "tidy_mem_mb" = "tidyexposomics",
      "rex_mem_mb"  = "rexposome"
    ),
    facet_label = paste("n =", n_samples, "samples") |>
      factor(levels = paste("n =", n_samples_vec, "samples"))
  ) |>
  ggplot(aes(x = n_features, y = memory_mb, color = package)) +
  geom_line(linewidth = 1) +
  geom_point(size = 3) +
  facet_wrap(~ facet_label, nrow = 1) +
  scale_x_log10(labels = scales::comma) +
  scale_y_log10() +
  labs(
    title = "Memory Usage by Feature Count",
    x     = "Number of Omics Features",
    y     = expression("Log"[10]*"[Memory (MB)]"),
    color = "Package"
  ) +
  scale_color_manual(values = c(
    "rexposome"      = "#3A68AE",
    "tidyexposomics" = "#883268"
  )) +
  theme_minimal() +
  theme(legend.position = "bottom")

m_v_f
```

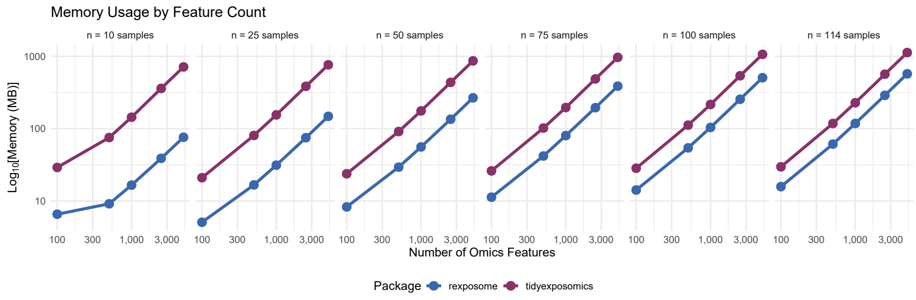


**Figure 3:** Figure 1c. Memory usage by omics feature count, faceted by sample size.

```
m_v_s <- memory_results |>
  pivot_longer(
```

```
    cols      = c(tidy_mem_mb, rex_mem_mb),
    names_to  = "package",
    values_to = "memory_mb"
  ) |>
  mutate(
    package = recode(package,
      "tidy_mem_mb" = "tidyexposomics",
      "rex_mem_mb"  = "rexposome"
    ),
    facet_label = paste(scales::comma(n_features), "features") |>
      factor(levels = paste(scales::comma(n_features_vec), "features"))
  ) |>
  ggplot(aes(x = n_samples, y = memory_mb, color = package)) +
  geom_line(linewidth = 1) +
  geom_point(size = 3) +
  facet_wrap(~ facet_label, nrow = 1) +
  scale_y_log10() +
  labs(
    title = "Memory Usage by Sample Size",
    x     = "Number of Samples",
    y     = expression("Log"[10]*"[Memory (MB)]"),
    color = "Package"
  ) +
  scale_color_manual(values = c(
    "rexposome"      = "#3A68AE",
    "tidyexposomics" = "#883268"
  )) +
  theme_minimal() +
  theme(legend.position = "bottom")

m_v_s
```

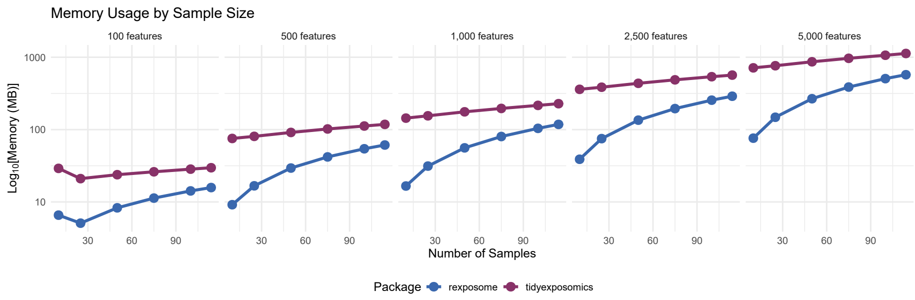


**Figure 4:** Figure 1d. Memory usage by sample size, faceted by feature count.

## 6.4 Combined Figure

```
(t_v_s / t_v_f / m_v_s / m_v_f) +
  plot_layout(guides = "collect") +
  plot_annotation(tag_levels = "a") &
  theme(
```

```
        legend.position = "bottom",
        plot.tag        = element_text(face = "bold")
      )
```

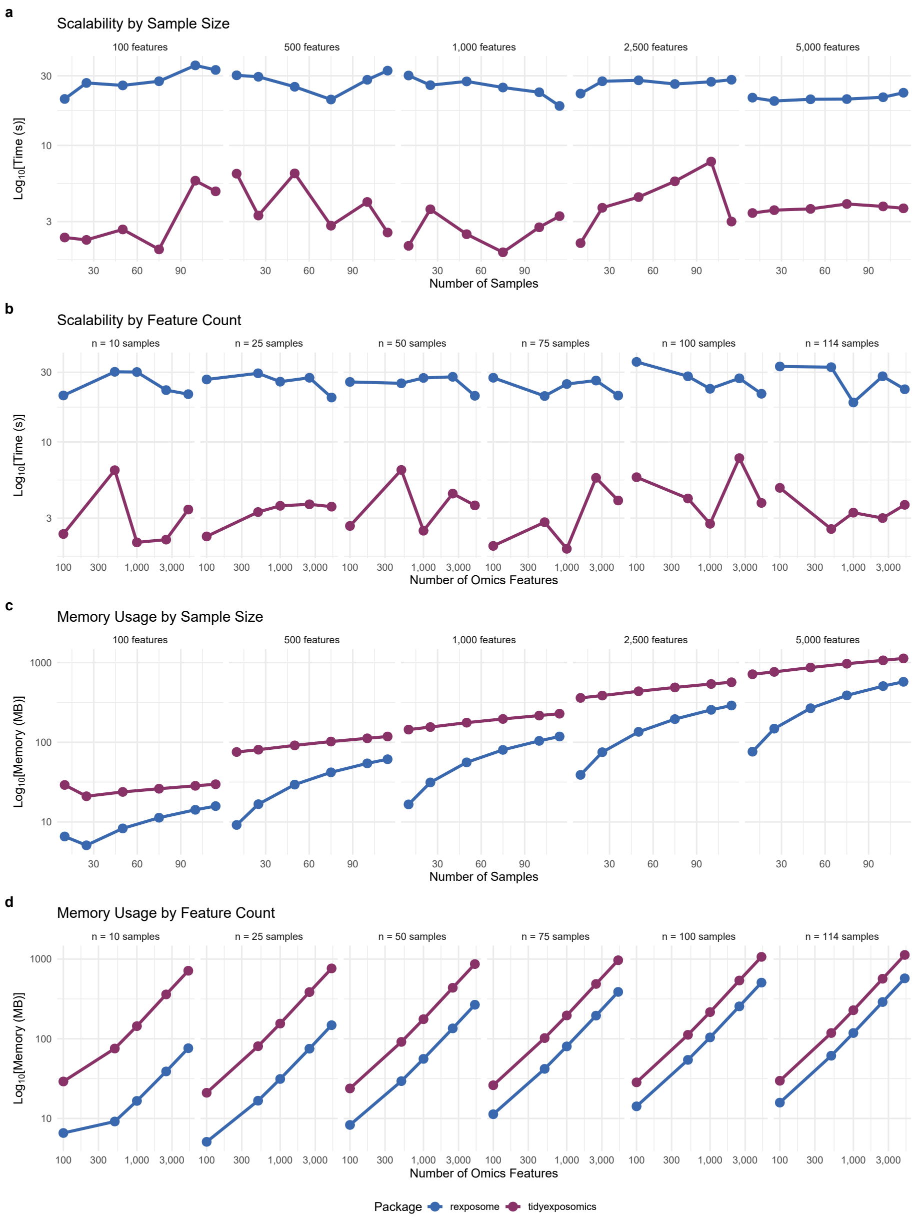


**Figure 5:** Figure 1. Comparison between rexposome/omicRexposome and tidyexposomics performance. a) The number of samples versus the Log10 of the runtime in seconds faceted by the number of omics features included in the exposure-omics association testing. b) The number of omics features versus the Log10 of the runtime in seconds faceted by the number of samples included in the exposure-omics association testing. c) The number of samples versus the Log10 of the memory used in megabytes (MB) faceted by the number of omics features included in the exposure-omics association testing. d) The number of omics features versus the Log10 of the memory used in megabytes (MB) faceted by the number of samples included in the exposure-omics association testing.

# 7 Session Info

```
sessionInfo()
## R version 4.5.1 (2025-06-13 ucrt)
## Platform: x86_64-w64-mingw32/x64
## Running under: Windows 11 x64 (build 26200)
##
## Matrix products: default
##   LAPACK version 3.12.1
##
## locale:
## [1] LC_COLLATE=English_United States.utf8
## [2] LC_CTYPE=English_United States.utf8
## [3] LC_MONETARY=English_United States.utf8
## [4] LC_NUMERIC=C
## [5] LC_TIME=English_United States.utf8
##
## time zone: America/New_York
## tzcode source: internal
##
## attached base packages:
## [1] stats4    stats     graphics  grDevices utils     datasets  methods
## [8] base
##
## other attached packages:
##  [1] patchwork_1.3.2             MultiDataSet_1.38.0
##  [3] bench_1.1.4                 omicRexposome_1.32.0
##  [5] rexposome_1.32.1            tidyexposomics_0.99.16
##  [7] MultiAssayExperiment_1.36.2 SummarizedExperiment_1.40.0
##  [9] Biobase_2.70.0              GenomicRanges_1.62.1
## [11] Seqinfo_1.0.0               IRanges_2.44.0
## [13] S4Vectors_0.48.0            BiocGenerics_0.56.0
## [15] generics_0.1.4              MatrixGenerics_1.22.0
## [17] matrixStats_1.5.0           scales_1.4.0
## [19] lubridate_1.9.5             forcats_1.0.1
## [21] stringr_1.6.0               dplyr_1.1.4
## [23] purrr_1.2.2                 readr_2.2.0
## [25] tidyr_1.3.2                 tibble_3.3.0
## [27] ggplot2_4.0.3               tidyverse_2.0.0
## [29] BiocStyle_2.38.0
##
## loaded via a namespace (and not attached):
##   [1] bitops_1.0-9         naniar_1.1.0         httr_1.4.8
##   [4] RColorBrewer_1.1-3   tools_4.5.1          backports_1.5.0
##   [7] R6_2.6.1             DT_0.34.0            mgcv_1.9-3
##  [10] withr_3.0.2          gridExtra_2.3        cli_3.6.5
##  [13] factoextra_2.0.0     Cairo_1.7-0          flashClust_1.1-4
##  [16] RGCCA_3.0.3          sandwich_3.1-1       labeling_0.4.3
##  [19] JADE_2.0-4           mvtnorm_1.4-1        S7_0.2.1
##  [22] genefilter_1.92.0    foreign_0.8-90       parallelly_1.47.0
```

```
##  [25] limma_3.66.0         rstudioapi_0.18.0    impute_1.84.0
##  [28] RSQLite_2.4.6        shape_1.4.6.1        gtools_3.9.5
##  [31] car_3.1-5            SmartSVA_0.1.3       leaps_3.2
##  [34] qqman_0.1.9          Matrix_1.7-4         PMA_1.2-4
##  [37] imputeLCMD_2.1       abind_1.4-8          lifecycle_1.0.5
##  [40] edgeR_4.8.2          scatterplot3d_0.3-45 yaml_2.3.12
##  [43] carData_3.0-6        qvalue_2.42.0        gplots_3.3.0
##  [46] recipes_1.3.3        SparseArray_1.10.8   BiocFileCache_3.0.0
##  [49] grid_4.5.1           blob_1.3.0           promises_1.5.0
##  [52] crayon_1.5.3         profmem_0.7.0        lattice_0.22-7
##  [55] annotate_1.88.0      KEGGREST_1.50.0      made4_1.84.0
##  [58] pillar_1.11.1        knitr_1.51           boot_1.3-32
##  [61] estimability_1.5.1   corpcor_1.6.10       future.apply_1.20.2
##  [64] mixOmics_6.34.0      codetools_0.2-20     glue_1.8.0
##  [67] pcaMethods_2.2.0     data.table_1.18.2.1  png_0.1-8
##  [70] vctrs_0.6.5          Rdpack_2.6.6         omicade4_1.50.0
##  [73] gtable_0.3.6         assertthat_0.2.1     cachem_1.1.0
##  [76] gower_1.0.2          xfun_0.57            rbibutils_2.4.1
##  [79] S4Arrays_1.10.1      mime_0.13            prodlim_2026.03.11
##  [82] coda_0.19-4.1        reformulas_0.4.4     survival_3.8-3
##  [85] timeDate_4052.112    iterators_1.0.14     gmm_1.9-1
##  [88] tinytex_0.59         hardhat_1.4.3        fastICA_1.2-7
##  [91] lava_1.9.1           statmod_1.5.1        ipred_0.9-15
##  [94] nlme_3.1-168         fenr_1.8.2           bit64_4.6.0-1
##  [97] filelock_1.0.3       Deriv_4.2.0          tmvtnorm_1.7
## [100] irlba_2.3.7          KernSmooth_2.23-26   otel_0.2.0
## [103] rpart_4.1.24         colorspace_2.1-2     DBI_1.3.0
## [106] Hmisc_5.2-5          ade4_1.7-24          nnet_7.3-20
## [109] tidyselect_1.2.1     emmeans_2.0.3        bit_4.6.0
## [112] compiler_4.5.1       curl_7.0.0           glmnet_5.0
## [115] lsr_0.5.2            httr2_1.2.2          htmlTable_2.5.0
## [118] DelayedArray_0.36.0  bookdown_0.46        checkmate_2.3.4
## [121] caTools_1.18.3       multcompView_0.1-11  rappdirs_0.3.4
## [124] digest_0.6.39        minqa_1.2.8          rmarkdown_2.31
## [127] XVector_0.50.0       htmltools_0.5.9      pkgconfig_2.0.3
## [130] base64enc_0.1-6      lme4_2.0-1           FactoMineR_2.15
## [133] dbplyr_2.5.2         fastmap_1.2.0        rlang_1.2.0
## [136] GlobalOptions_0.1.4  htmlwidgets_1.6.4    shiny_1.13.0
## [139] farver_2.1.2         zoo_1.8-15           jsonlite_2.0.0
## [142] BiocParallel_1.44.0  ModelMetrics_1.2.2.2 magrittr_2.0.4
## [145] Formula_1.2-5        Rcpp_1.1.1           visdat_0.6.0
## [148] stringi_1.8.7        pROC_1.19.0.1        MASS_7.3-65
## [151] plyr_1.8.9           parallel_4.5.1       listenv_0.10.1
## [154] ggrepel_0.9.8        Biostrings_2.78.0    splines_4.5.1
## [157] hms_1.1.4            circlize_0.4.18      locfit_1.5-9.12
## [160] igraph_2.3.2         ggpubr_0.6.3         ggsignif_0.6.4
## [163] reshape2_1.4.5       XML_3.99-0.22        tidybulk_2.0.1
## [166] evaluate_1.0.5       calibrate_1.7.7      BiocManager_1.30.27
## [169] isva_1.10            nloptr_2.2.1         tzdb_0.5.0
## [172] foreach_1.5.2        httpuv_1.6.17        clue_0.3-68
## [175] future_1.70.0        norm_1.0-11.1        BiocBaseUtils_1.12.0
```

```
## [178] broom_1.0.13        xtable_1.8-8        RSpectra_0.16-2
## [181] rstatix_0.7.3       later_1.4.8         class_7.3-23
## [184] rARPACK_0.11-0      AnnotationDbi_1.72.0 memoise_2.0.1
## [187] ellipse_0.5.0       cluster_2.1.8.2     corrplot_0.95
## [190] sva_3.58.0          timechange_0.4.0    globals_0.19.1
## [193] caret_7.0-1
```
