# Supplementary material for: tidyexposomics: integrated exposure-omics analysis powered by tidy principles
Source: Bioinform Adv. 2026 Aug 8;6(1):vbag220. doi: 10.1093/bioadv/vbag220 (PMC13532756; doi:10.1093/bioadv/vbag220)
Supplement: Supplementary file 1 [file vbag220_supplementary_data.zip › tidyexposomics_supplemental_file_2.pdf]

tidyexposomics: Integrated exposure-omics analysis powered by tidy principles

Supplemental File 2: tidyexposomics vignette

Jason Laird[1,2], Thomas Hartung[1,2,3,4], Fenna C. M. Sillé[1,2], Alexandra Maertens[1,2]*

[1] Department of Environmental Health and Engineering, Bloomberg School of Public Health, Johns Hopkins University, MD, USA

[2] Center for Alternatives to Animal Testing (CAAT), Bloomberg School of Public Health and Whiting School of Engineering, Johns Hopkins University, MD, USA

[3] CAAT-Europe, University of Konstanz, Konstanz, Germany

[4] Doerenkamp-Zbinden Chair for Evidence-based Toxicology, Department of Environmental Health and Engineering, Johns Hopkins University, Baltimore, MD, USA

** Corresponding Author: Alexandra Maertens, PhD*

3 November 2025

Contents

1 Installation . . . 3

2 Command Structure . . . 4

3 Exposure Metadata and Ontology Annotation . . . 4

- 3.1 Codebook Setup . . . 4
- 3.2 Ontology Choices . . . 4
- 3.3 Ontology Annotation App . . . 5

4 Loading Data . . . 6

5 Quality Control . . . 8

- 5.1 Missingness . . . 8
- 5.2 Imputation . . . 9
- 5.3 Filtering Omics Features . . . 10
- 5.4 Normality Check . . . 11
- 5.5 Principal Component Analysis . . . 11
- 5.6 Exposure Summary . . . 13
- 5.7 Exposure Visualization . . . 14

6 Sample-Exposure Association . . . 15

- 6.1 Sample Clustering . . . 15

6.2 Exposure Correlations . . . 16
6.3 Exposure-wide association (ExWAS) . . . 17
6.4 Exposome Scores . . . 19

7 Differential Abundance . . . 22
7.1 Differential Abundance . . . 22
7.2 Sensitivity Analysis . . . 23

8 Multi-Omics Integration . . . 25
8.1 Factor Analysis . . . 26

9 Exposure-Omics Association . . . 29
9.1 Exposure-Omics Association . . . 29
9.2 Network Analysis . . . 31
9.3 Exposure-Omics Impact Network Analysis . . . 33

10 Enrichment Analysis . . . 35
10.1 Enrichment Visualizations . . . 36

11 Custom Analysis . . . 41
11.1 Pivot Sample . . . 41
11.2 Pivot Feature . . . 42
11.3 Pivot Experiment . . . 43

12 Pipeline Summary . . . 45
12.1 Saving Results . . . 45
12.2 Session Info . . . 46
12.3 References . . . 48

Exposure to environmental factors is a major determinant of health and disease. The exposome is a term that represents the totality of environmental exposures (or in practical terms, the integrated compilation of all physical, chemical, biological, and (psycho)social influences that impact biology) from conception to death, that drive disease and overall health (Miller & Banbury Exposomics Consortium, 2025; Wild, 2005). Characterizing the relationship between a multiplicity of exposures and disease outcomes can be a daunting task. Epidemiological studies have moved towards exposure-wide association studies (ExWAS) where sets of exposures are associated with an outcome (Chung et al., 2024). The advent of high-throughput technologies has enabled profiling of several layers of biological information. These layers can also be integrated to better understand the relationship between exposures and disease outcomes.

The `tidyexposomics` package is designed to facilitate the integration of exposure and omics data to identify exposure-omics associations and their relevance to health outcomes.`tidyexposomics` extends the tidy-Bioconductor ecosystem (e.g., tidybulk, tidySummarizedExperiment) to exposome multi-omics integration using the MultiAssayExperiment container. It provides tidyverse-style accessors and functions for association testing, multi-omics integration, and ontology-driven enrichment, in an effort to complement existing tidy-Bioc tools.

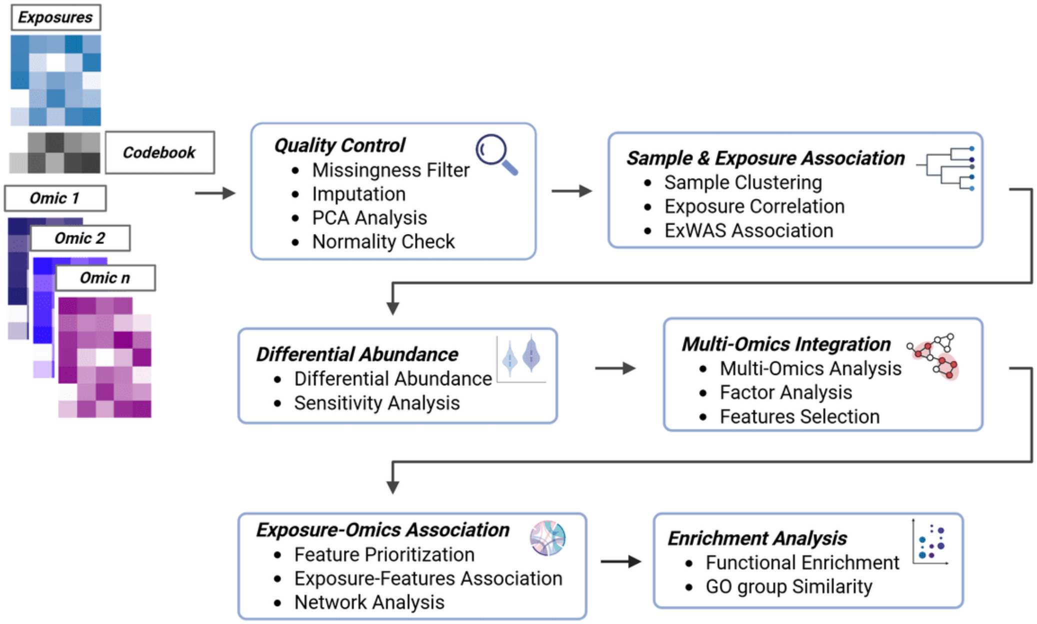

Figure 1: tidyexposomics pipeline overview. QC, association testing, integration, and enrichment steps on a MultiAssayExperiment.

1 Installation

```
# install the package
BiocManager::install("tidyexposomics")

# load the package
library(tidyexposomics)
```

2 Command Structure

To make the package more user-friendly, we have named our functions to be more intuitive. For example, we use the following naming conventions:

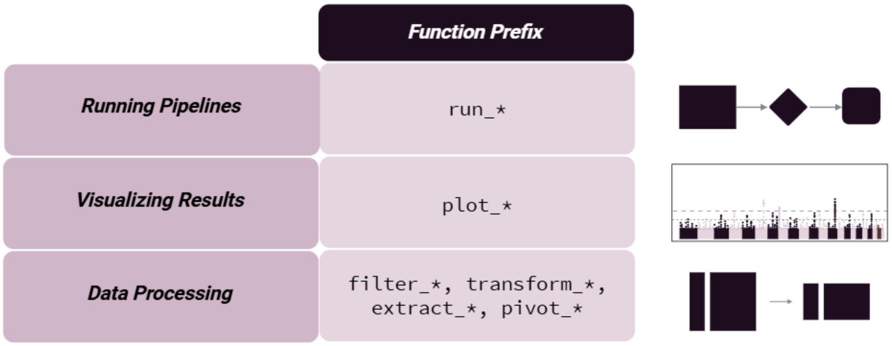

Figure 2: Command naming conventions used throughout `tidyexposomics`. Pipelines begin with `run_`, `plot_`, and `filter_` prefixes.

We provide functionality to either add results to the existing object storing the omics/exposure data or to return results directly using `action = "get"`. We suggest adding results, given that pipeline steps are tracked and can be output to the R console, plotted as a workflow diagram, or exported to an Excel worksheet.

3 Exposure Metadata and Ontology Annotation

3.1 Codebook Setup

Before starting an exposomics data analysis we recommend having a codebook, with information on your exposure variables. Some suggestions:

- **Variable Name**: The name of the variable in the data set.
- **Variable Description**: A concise description of what the variable measures, including units (e.g., "urinary bisphenol A (ng/mL)").
- **Variable Type**: The type of variable, such as continuous, categorical, or binary.
- **Variable Period**: The period of time over which the variable was measured, such as "lifetime", "year", "month", or "day".
- **Variable Location**: The location where the variable was measured, such as "home", "work", "school", or "geospatial code".
- **Variable Ontology**: The ontology term associated with the variable.

3.2 Ontology Choices

Variables captured in the codebook should be annotated with ontology terms to provide a standardized vocabulary for the variables. We recommend using the following ontologies for exposure and outcome variables:

- Environment Exposure Ontology to annotate your exposure variables.

- Human Phenotype Ontology to annotate your outcome variables and phenotypic data.
- Chemical Entities of Biological Interest to annotate your chemical exposure variables.

Why annotate with ontologies?

- **Interpretability**: Ontology labels clarify ambiguous or inconsistently named variables.
- **Harmonization**: You can compare and combine variables across datasets when they map to the same term.
- **Grouping**: Ontologies allow you to collapse fine-grained exposures into broader categories.
- **Integration**: Many public tools, knowledge graphs, and repositories are ontology-aware. This can make your results more interoperable and reusable.

3.3 Ontology Annotation App

To help annotate exposure variables, we provide a lightweight shiny app:

```
# Launch the shiny app to annotate exposure variables
shiny::runApp(build_ont_annot_app())
```

To use the app:

- Click **Browse** to select your exposure metadata file.
- Then you can click the variable you'd like to link to an annotation term and search in the **Choose Ontology Term** dropdown.
- After you select a term, you will see a short description of the term.
- After you are done, click **Apply Annotate** to save the annotation.
- Now you can group exposures into larger categories by selecting each line and then choose your ontology and root depth level (where a lower number means a more general term).
- Then you can click **Apply Categorization** to apply the selected categorization to the selected rows.
- If the ontology has nothing to do with your variable, you may manually enter a category in the **Category** column. This will change the **Category Source** to manual and will not be linked to the ontology.
- Once you have annotated all your variables click **Download Annotated CSV** to save the annotated metadata file.

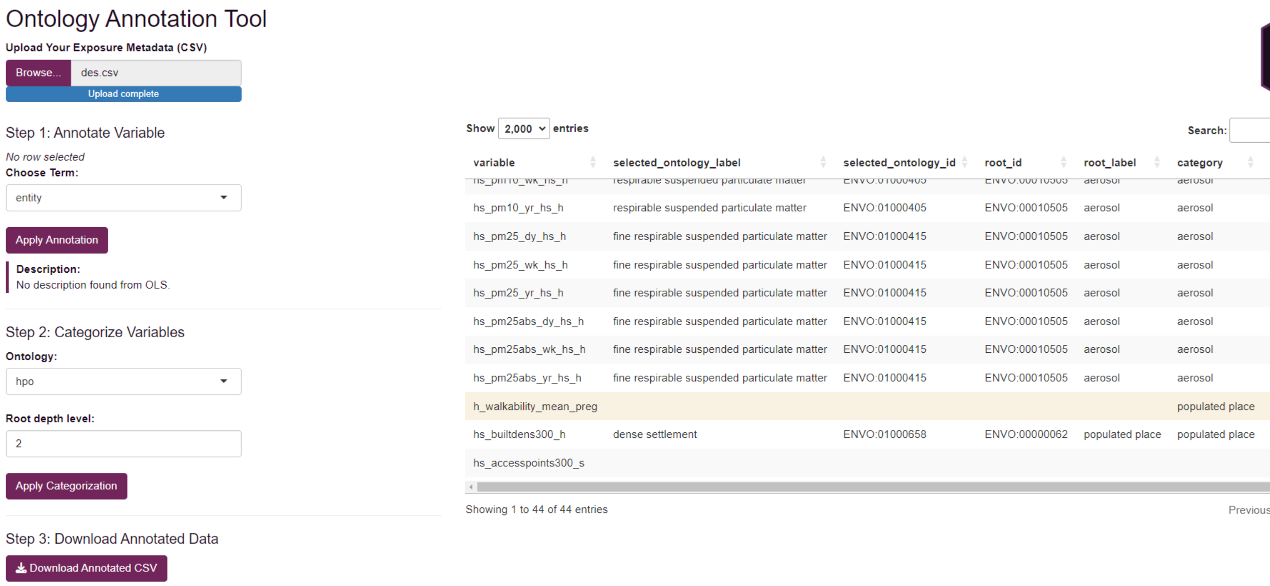

Figure 3: Screenshot of the ontology annotation app. The sidebar has an upload button to load your exposure metadata file. The main panel displays the uploaded exposure metadata where users can select variables to annotate and categorize. The sidebar also contains buttons to apply annotations/categorizations and download the annotated file.

4 Loading Data

To get started we need to load the data. The `create_exposomicset` function is used to create a `MultiAssayExperiment` object that contains exposure and omics data. As a quick introduction, a `MultiAssayExperiment` object is a container for storing multiple assays (e.g., omics data) and their associated metadata:

We use the MultiAssayExperiment object to store the exposure and omics data. The create_expomicset function has several arguments:

- `codebook`: is a data frame that contains information about the variables in the exposure metadata. The column names must contain variable where the values are the column names of the exposure data frame, and category which contains general categories for the variable names. This is the data frame you created with the ontology annotation app!
- `exposure`: is a data frame that contains the exposure and other metadata.
- `omics`: is a list of data frames that contain the omics data.
- `row_data`: argument is a list of data frames that contain information about the rows of each omics data frame.

We are going to start by loading in example data pulled from the ISGlobal Exposome Data Challenge 2021 (Maitre et al., 2022). Specifically, we will examine how exposures and omics features relate to asthma status in asthma patients with a lower socioeconomic status (SES).

```
# Load Libraries
library(tidyverse)
library(tidyexposomics)
```

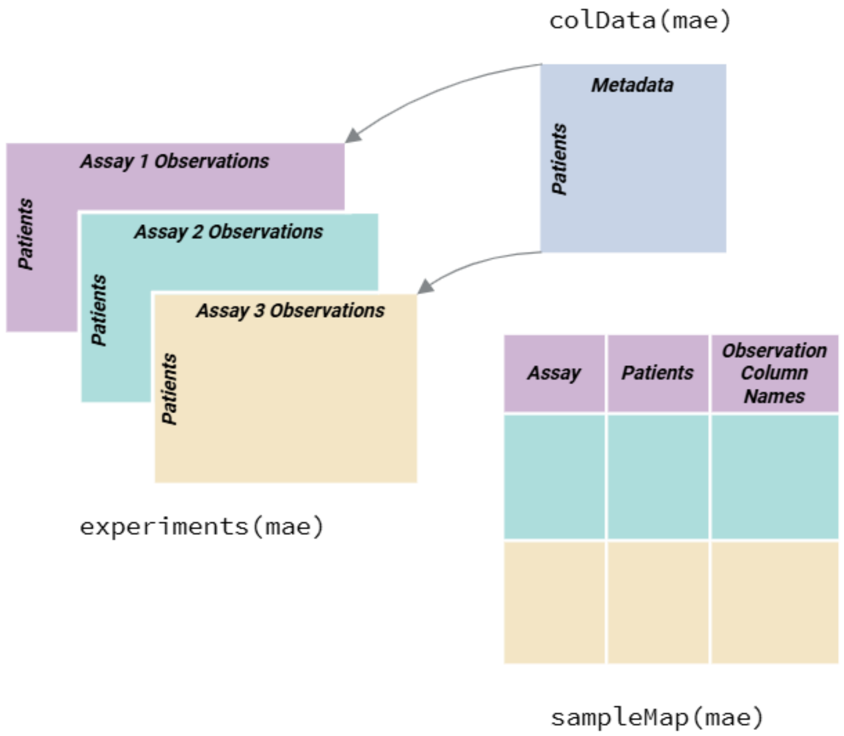

Figure 4: Overview of the MultiAssayExperiment structure linking samples, assays, and metadata.

```
# Load example data
omics_list <- download_dataset("omics_list")
fdata <- download_dataset("fdata")
meta <- download_dataset("meta")
annotated_cb <- download_dataset("annotated_cb")

# Create exposomic set object
expom <- create_exposomicset(
    codebook = annotated_cb,
    exposure = meta[meta$FAS_cat_None=="Low",],
    omics = list(
        "Gene Expression" = omics_list$`Gene Expression`,
        "Methylation" = omics_list$Methylation
    ),
    row_data = list(
        "Gene Expression" = fdata$`Gene Expression`,
        "Methylation" = fdata$Methylation
    )
)

# Keeping only samples from low socio-economic status and where the
# asthma status is not NA
expom <- expom[, !is.na(expom$hs_asthma)]
```

```r
expom <- expom[, expom$FAS_cat_None == "Low"]
```

We are interested in how the exposome affects health outcomes, so let's define which metadata variables represent exposure variables.

```r
# Grab exposure variables
exp_vars <- annotated_cb |>
  filter(category %in% c(
    "exposure to oxygen molecular entity",
    "aerosol",
    "environmental zone",
    "main group molecular entity",
    "transition element molecular entity",
    "exposure to environmental process",
    "polyatomic entity"
  )) |>
  pull(variable) |>
  as.character()
```

5 Quality Control

5.1 Missingness

Oftentimes when collecting data, there are missing values. Let's use the `plot_missing` function to determine where our missing values are:

```r
plot_missing(
    exposomicset = expom,
    plot_type = "summary",
    threshold = 0
)
```

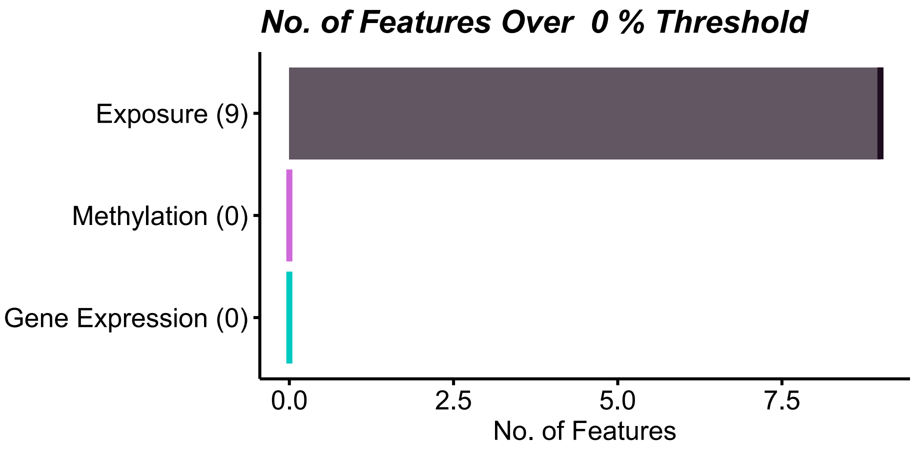

Figure 5: Count of features with missing data above a 0% missingness threshold by data layer. Exposure data have variables with missingness.

Here we see that there are 4 variables in the exposure data that are missing data. Let's take a look at them:

```
plot_missing(
    exposomicset = expom,
    plot_type = "lollipop",
    threshold = 0,
    layers = "Exposure"
)
```

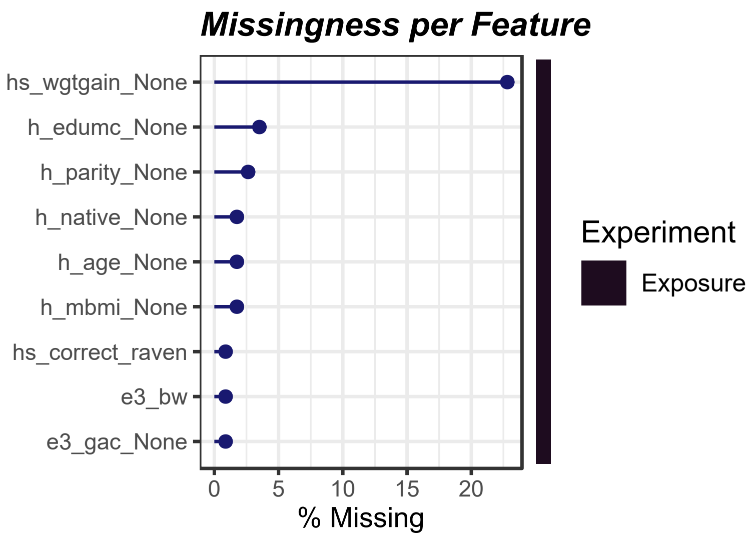

Figure 6: Percent missingness per exposure variable. Maternal weight gain during pregnancy, `hs_wgtgain_None`, shows the highest missingness.

Here we see that one variable, `hs_wgtgain_None`, has about 25% missing values. We can apply a missingness filter using the `filter_missing` function. The `na_thresh` argument is used to set the threshold for missing values. For example, if `na_thresh = 20`, then any variable with more than 20% missing values will be removed. Here we set the threshold to 5% to filter out variables with more than 5% missing values.

```
# Filter out variables with too many missing values
expom <- filter_missing(
  exposomicset = expom,
  na_thresh = 5)
```

5.2 Imputation

The `run_impute_missing` function is used to impute missing values. Here we can specify the imputation method for exposure and omics data separately.

The `exposure_impute_method` argument is used to set the imputation method for exposure data, and the `omics_impute_method` argument is used to set the imputation method for omics data. The `omics_to_impute` argument is used to specify which omics data to impute. Here we will impute the exposure data given using the `missforest` method, but other options for imputation methods include:

- `median`: Imputes missing values with the median of the variable.

- `mean`: Imputes missing values with the mean of the variable.
- `knn`: Uses k-nearest neighbors to impute missing values.
- `mice`: Uses the Multivariate Imputation by Chained Equations (MICE) method to impute missing values.
- `missforest`: Uses the MissForest method to impute missing values.
- `lod_sqrt2`: Imputes missing values using the square root of the lower limit of detection (LOD) for each variable. This is useful for variables that have a lower limit of detection, such as chemical exposures.

```
# Impute missing values
expom <- run_impute_missing(
    exposomicset = expom,
    exposure_impute_method = "missforest",
    exposure_cols = exp_vars
)
```

5.3 Filtering Omics Features

We can filter omics features based on variance or expression levels. The `filter_omics` function is used to filter omics features. The method argument is used to set the method for filtering. Here we can use either:

- **Variance**: Filters features based on variance. We recommend this for omics based on continuous measurements, such as log-transformed counts, M-values, protein intensities, or metabolite concentrations.
- **Expression**: Filters features based on expression levels. We recommend this for omics where many values may be near-zero or zero, such as RNA-seq data.

The `assays` argument is used to specify which omics data to filter. The `assay_name` argument is used to specify which assay to filter. The `min_var`, `min_value`, and `min_prop` arguments are used to set the minimum variance, minimum expression value, and minimum proportion of samples exceeding the minimum value, respectively.

```
# filter omics layers by variance and expression
# Methylation filtering
expom <- filter_omics(
    exposomicset = expom,
    method = "variance",
    assays = "Methylation",
    assay_name = 1,
    min_var = 0.05
)

# Gene expression filtering
expom <- filter_omics(
    exposomicset = expom,
    method = "expression",
    assays = "Gene Expression",
    assay_name = 1,
    min_value = 1,
```

```
    min_prop = 0.3
)
```

5.4 Normality Check

When determining variable associations, it is important to check the normality of the data. The `run_normality_check` function is used to check the normality of the data.

The `transform_exposure` function is used to transform the data to make it more normal. Here the transform_method is set to `boxcox_best` as it will automatically select the best transformation method based on the data. The `transform_method` can be manually set to `log2`, `sqrt`, or `x_1_3` as well. We specify the exposure_cols argument to set the columns to transform.

```
# Check variable normality
expom <- run_normality_check(
    exposomicset = expom,
    action = "add"
)

# Transform variables
expom <- transform_exposure(
    exposomicset = expom,
    transform_method = "boxcox_best",
    exposure_cols = exp_vars
)
```

To check the normality of the exposure data, we can use the `plot_normality_summary` function. This function plots the normality of the data before and after transformation. The `transformed` argument is set to `TRUE` to plot the normality status of the transformed data.

```
plot_normality_summary(
    exposomicset = expom,
    transformed = TRUE
)
```

5.5 Principal Component Analysis

To identify the variability of the data, we can perform a principal component analysis (PCA). The `run_pca` performs a joint PCA across all numeric exposures and omic assays after standardization, identifying shared axes of variation across layers. The resulting PCs in `colData()` reflect integrated sample-level variance across all data types, and outliers are defined in that joint multi-omics PC space.

Here we specify that we would like to log-transform the exposure and omics data before performing PCA using the `log_trans_exp` and the `log_trans_omics` arguments, respectively. We automatically identify sample outliers based on the Mahalanobis distance, a measure of the distance between a point and a distribution.

```
# Perform principal component analysis
expom <- run_pca(
```

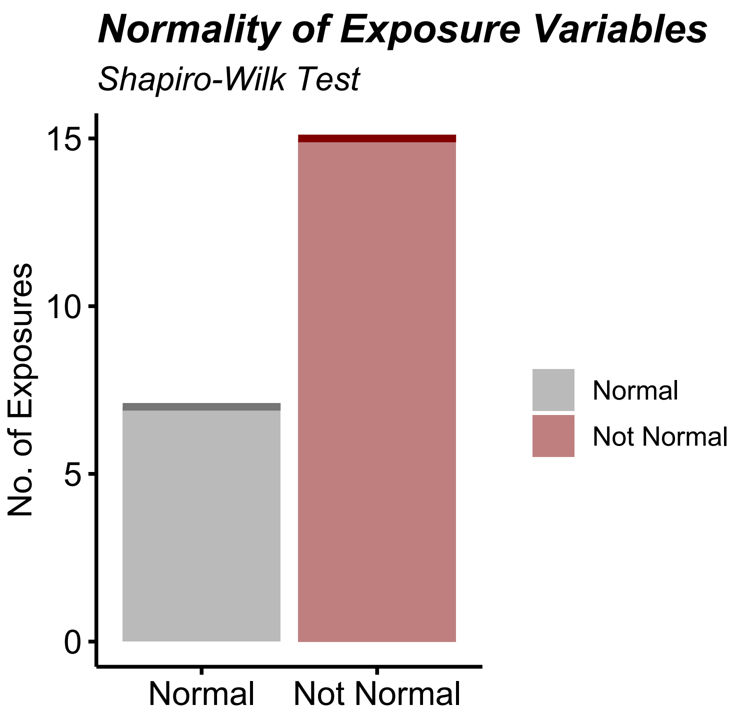

Figure 7: Normality status of numeric exposure variables after Box-Cox transformation.

```
    exposomicset = expom,
    log_trans_exp = TRUE,
    log_trans_omics = TRUE,
    action = "add"
)
```

```
plot_pca(exposomicset = expom)
```

Here we see six sample outliers, and that most variation is captured in the first two principal components for both features and samples. We can filter out the outliers using the `filter_sample_outliers` function.

```
# Filter out sample outliers
expom <- filter_sample_outliers(
    exposomicset = expom,
    outliers = c("s73", "s1183", "s376", "s899", "s828", "s993")
)
```

To understand the relationship between the principal components and exposures we can correlate them using the `run_correlation` function. Here we specify that the `feature_type` is `pcs` for principal components, specify a set of exposure variables, `exp_vars`, and the number of principal components, `n_pcs`. We set `correlation_cutoff` to `0` and `pval_cutoff` to `1` to initially include all correlations.

```
expom <- run_correlation(
    exposomicset = expom,
```

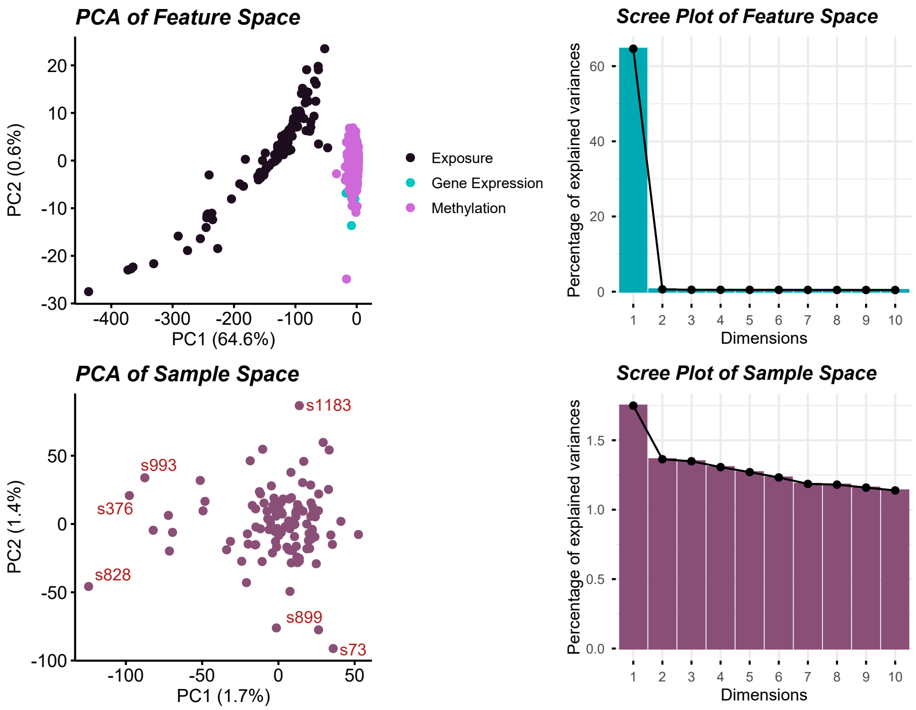

Figure 8: PCA of sample and feature space with sample outlier detection.

```
    feature_type = "pcs",
    exposure_cols = exp_vars,
    n_pcs = 20,
    action = "add",
    correlation_cutoff = 0,
    pval_cutoff = 1
)
```

We can visualize these correlations with the `plot_correlation_tile` function. We specify we are plotting the `feature_type` of `pcs` to grab the principal component correlation results. We then set the significance threshold to 0.05 with the `pval_cutoff` argument.

```
plot_correlation_tile(
    exposomicset = expom,
    feature_type = "pcs",
    pval_cutoff = 0.05
)
```

5.6 Exposure Summary

We can summarize the exposure data using the `run_summarize_exposures` function. This function calculates summary statistics for each exposure variable, including the number of values, number of missing values, minimum, maximum, range, sum, median, mean, standard error, and confidence intervals. The `exposure_cols` argument determines which variables to include in the summary.

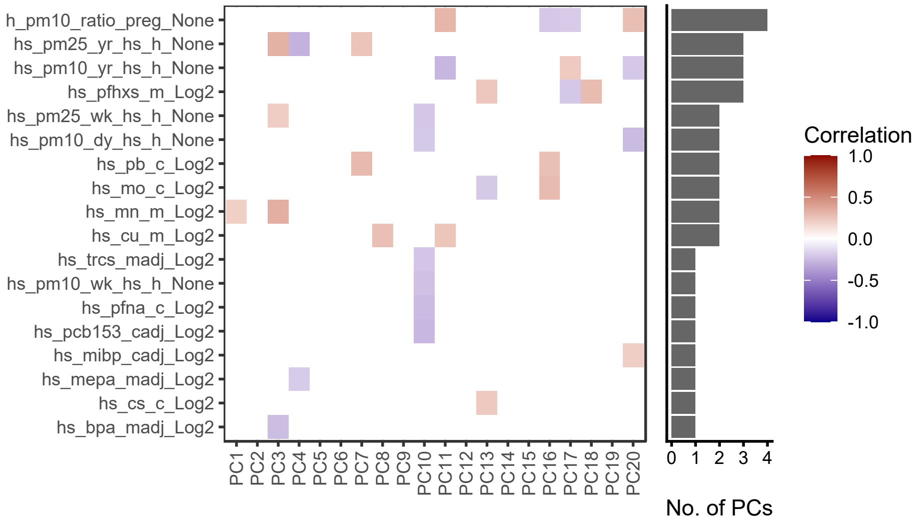

Figure 9: Correlation heatmap of exposures versus principal components. Particulate matter levels, `h_pm10_ratio_preg_None` and `hs_pm25_yr_hs_h_None`, are associated with the most principal components.

```
# Summarize exposure data
run_summarize_exposures(
    exposomicset = expom,
    action = "get",
    exposure_cols = exp_vars
) |>
    head()
## # A tibble: 6 x 27
##   variable   n_values  n_na   min   max range    sum median  mean    se ci_lower
##   <chr>         <dbl> <dbl> <dbl> <dbl> <dbl>  <dbl>  <dbl> <dbl> <dbl>    <dbl>
## 1 h_pm10_ra~      108     0  0.14  0.29  0.14   21.7   0.2   0.2   0         0.2
## 2 h_pm25_ra~      108     0  3.06  4.66  1.6   421.    3.87  3.9   0.03      3.85
## 3 hs_bpa_ma~      108     0  0    12.8  12.8   973.    9.1   9.01  0.14      8.73
## 4 hs_cs_c_L~      108     0  0     2.35  2.35  123.    1.07  1.14  0.05      1.04
## 5 hs_cu_m_L~      108     0  9.89 11.1   1.17 1127.   10.4  10.4   0.03     10.4
## 6 hs_mepa_m~      108     0  0.86 14.1  13.2   729.    7.29  6.75  0.24      6.27
## # i 16 more variables: ci_upper <dbl>, variance <dbl>, sd <dbl>,
## #   coef_var <dbl>, period <chr>, location <chr>, description <chr>,
## #   var_type <chr>, transformation <chr>, selected_ontology_label <chr>,
## #   selected_ontology_id <chr>, root_id <chr>, root_label <chr>,
## #   category <chr>, category_source <chr>, transformation_applied <chr>
```

5.7 Exposure Visualization

To visualize our exposure data, we can use the `plot_exposures` function. This function allows us to plot the exposure data in a variety of ways. Here we will plot the exposure data using a boxplot. The `exposure_cat` argument is used to set the exposure category to plot. Additionally, we could specify `exposure_cols` to only plot certain exposures. The `plot_type` argument is used to set the type of plot to create. Here we use a boxplot, but we could also use a ridge plot.

```
plot_exposures(
    exposomicset = expom,
    group_by = "e3_sex_None",
    exposure_cat = "aerosol",
    plot_type = "boxplot",
    ylab = "Values",
    title = "Aerosol Exposure by Sex"
)
```

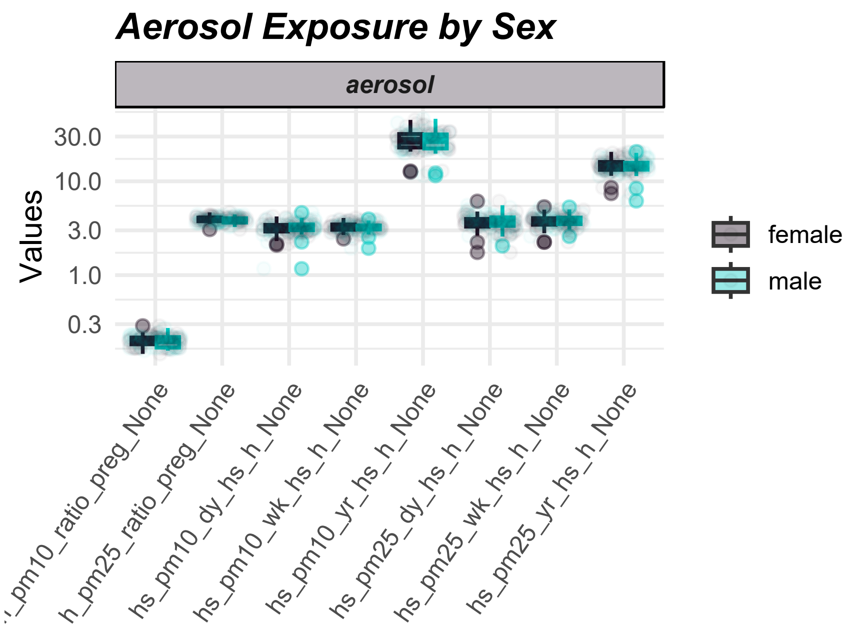

Figure 10: Distribution of aerosol exposures by sex.

Here we do not see any significant differences in aerosol exposure between males and females.

6 Sample-Exposure Association

6.1 Sample Clustering

The `run_cluster_samples` function is used to cluster samples based on the exposure data, clustering approaches are available by setting the `clustering_approach` argument. Here we use the `dynamic` approach, which uses a dynamic tree cut method to identify clusters. Other options are:

- `gap`: **Gap statistic method (default)**; estimates optimal `k` by comparing within-cluster dispersion to that of reference data.
- `diana`: **Divisive hierarchical clustering (DIANA)**; chooses `k` based on the largest drop in dendrogram height.
- `elbow`: **Elbow method**; detects the point of maximum curvature in within-cluster sum of squares (WSS) to determine `k`.

- `dynamic`: **Dynamic tree cut**; adaptively detects clusters from a dendrogram structure without needing to predefine `k`.
- `density`: **Density-based clustering** (via `densityClust`); identifies clusters based on local density peaks in distance space.

```
# Sample clustering
expom <- run_cluster_samples(
    exposomicset = expom,
    exposure_cols = exp_vars,
    clustering_approach = "dynamic",
    action = "add"
)
##  ..cutHeight not given, setting it to 1650  ===>  99% of the (truncated) height range in dendro.
##  ..done.
```

We plot the sample clusters using the `plot_sample_clusters` function. This function plots z-scored values of the exposure data for each sample, colored by the cluster assignment. The `exposure_cols` argument is used to set the columns to plot.

```
plot_sample_clusters(
    exposomicset = expom,
    exposure_cols = exp_vars
)
```

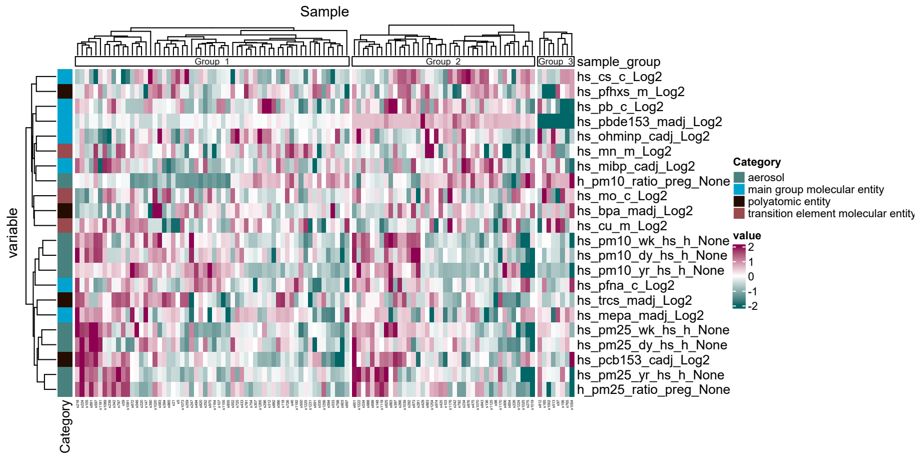

Figure 11: Sample clustering heatmap using exposure profiles (z-scored). Clusters appear mostly driven by aerosol exposure.

Here we see three clusters, largely driven by particulate matter/aerosol exposure.

6.2 Exposure Correlations

The `run_correlation` function identifies correlations between exposure variables. We set `feature_type` to `exposures` to focus on exposure variables and use a correlation cutoff of `0.3` to filter for meaningful associations. This cutoff can be adjusted based on your data and analysis needs.

```
expom <- run_correlation(
    exposomicset = expom,
    feature_type = "exposures",
    action = "add",
    exposure_cols = exp_vars,
    correlation_cutoff = 0.3
)
```

To visualize the exposure correlations, we can use the `plot_circos_correlation` function. Here we will plot the circos plot. This function creates a circular plot of the exposure correlations. The `correlation_cutoff` argument is used to set the minimum correlation score for the association. Here we use a cutoff of `0.3`.

```
plot_circos_correlation(
    exposomicset = expom,
    feature_type = "exposures",
    corr_threshold = 0.3,
    exposure_cols = exp_vars
)
```

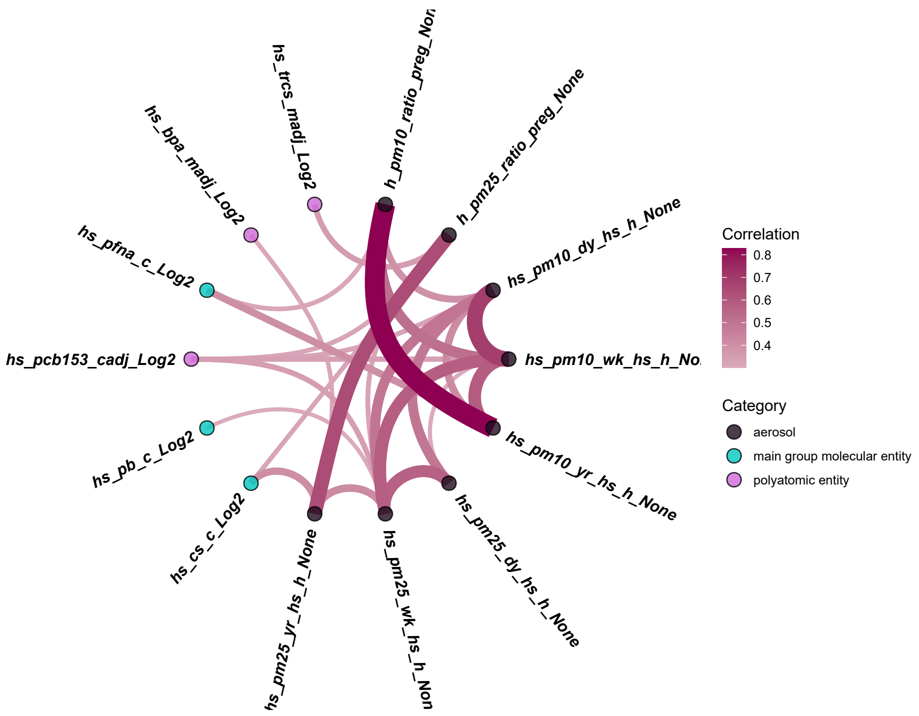

Figure 12: Circos view of exposure–exposure correlations (threshold 0.3).

6.3 Exposure-wide association (ExWAS)

The `run_association` function performs an ExWAS analysis to identify associations between exposures and outcomes. We specify the data source, outcome variable, feature set, and covariates for the analysis. Since we have a binary outcome, we set the model family to binomial.

```
# Perform ExWAS Analysis
expom <- run_association(
  exposomicset = expom,
  source = "exposures",
  outcome = "hs_asthma",
  feature_set = exp_vars,
  action = "add",
  family = "binomial"
)
```

To visualize the results of the ExWAS analysis, we can use the `plot_association` function, which will plot results for the the specified features. The `terms` argument is used to set the features to plot. The `filter_thresh` argument is used to set the threshold for filtering the results. The `filter_col` argument is used to set the column to filter on. Here we use `p.value` to filter on the p-value of the association. We can also include the R^2 or adjusted R^2 (if covariates are included) using the `r2_col` argument.

```
plot_association(
    exposomicset = expom,
    source = "exposures",
    terms = exp_vars,
    filter_thresh = 0.05,
    filter_col = "p.value",
    r2_col = "r2"
)
```

Here we see that no exposure variables are significantly associated wit asthma status. Although we do see that confidence interval for child Mono-iso-butyl phthalate (MiBP) levels (`hs_mibp_cadj_Log2`) does not cross 0, indicating a positive, albeit not significant ($P < 0.05$) association.

We can also associate our omics features with an outcome of interest using the `run_association` function. Here we specify an additional argument, `top_n`, which is used to set the top number of high variance omics features to include per omics layer.

```
# Perform ExWAS Analysis
expom <- run_association(
    exposomicset = expom,
    outcome = "hs_asthma",
    source = "omics",
    top_n = 500,
    action = "add",
    family = "binomial"
)
```

```
plot_manhattan(
    exposomicset = expom,
    min_per_cat = 0,
    feature_col = "feature_clean",
    vars_to_label = c(
        "TC01000261.hg.1",
        "TC09001056.hg.1",
```

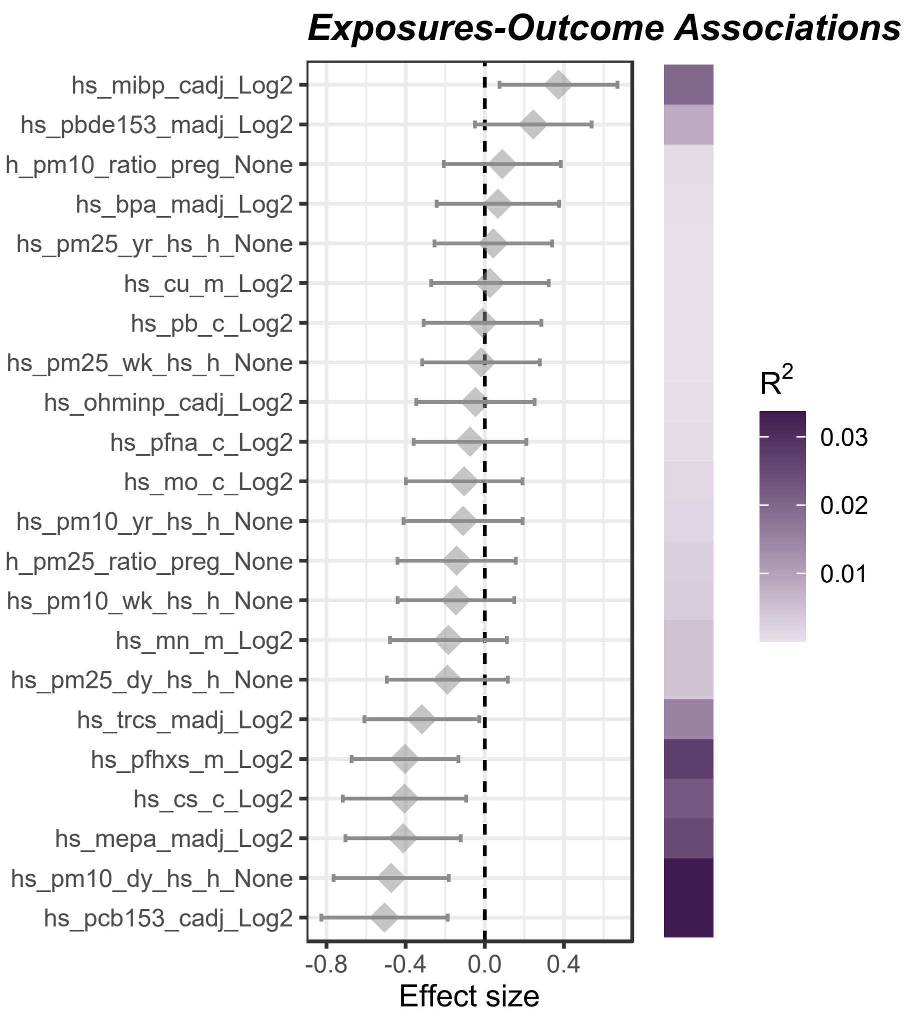

Figure 13: ExWAS associations of exposures with asthma status. No exposures are significantly associated ($P < 0.05$) with asthma status.

```
            "cg25575065",
            "hs_pm10_dy_hs_h_None"
        ),
        panel_sizes = c(1, 3, 1, 3, 1, 1, 1),
        facet_angle = 0
    )
```

6.4 Exposome Scores

We can also calculate exposome scores, which are a summary measure of exposure. The `run_exposome_score` function is used to calculate the exposome score. The `exposure_cols` argument is used to set the columns to use for the exposome score. The `score_type` argument is used to set the type of score to calculate. Here we could use:

- `median`: Calculates the median of the exposure variables.

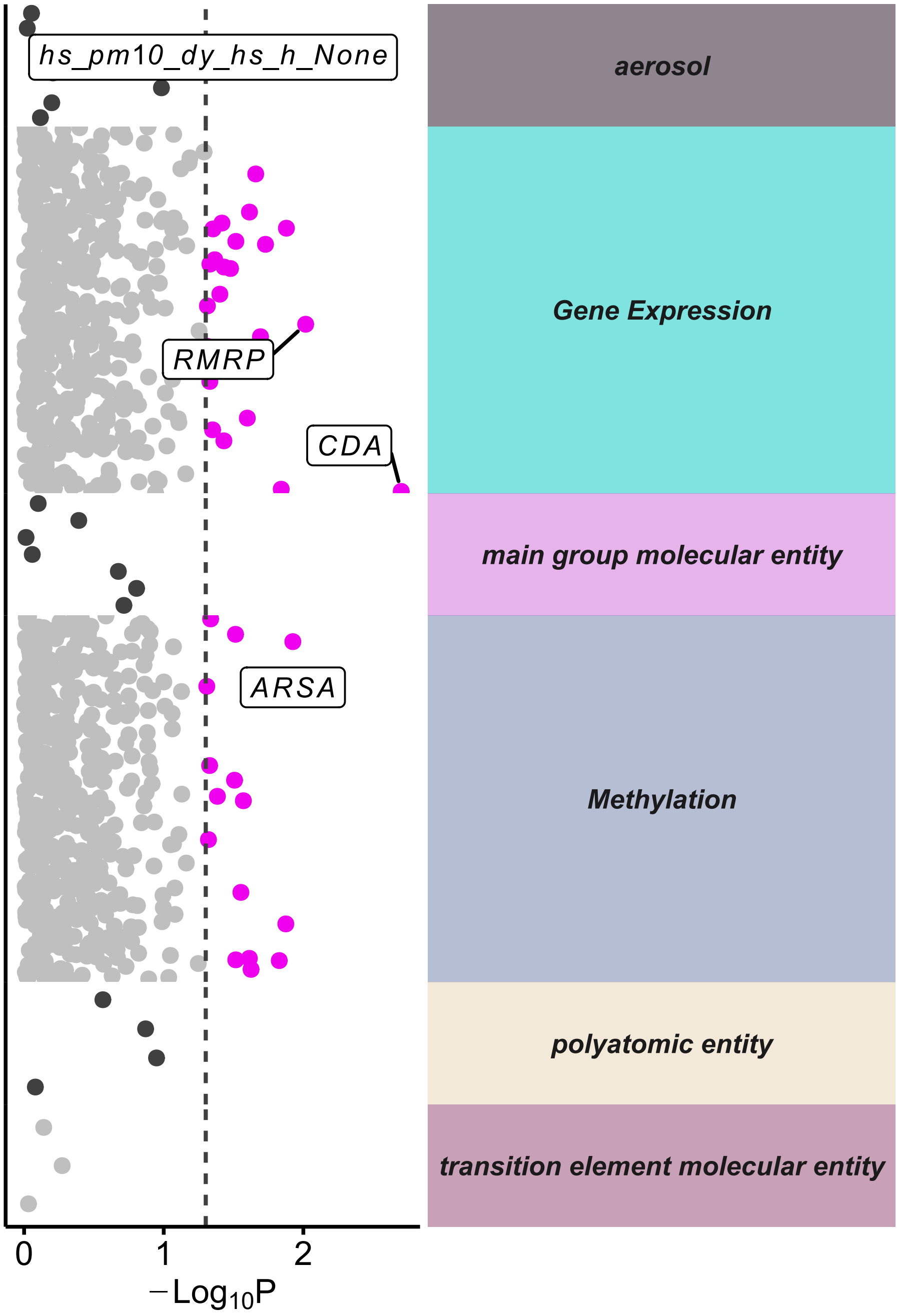

Figure 14: Manhattan plot of omics-wide associations with asthma status.

- `mean`: Calculates the mean of the exposure variables.
- `sum`: Calculates the sum of the exposure variables.
- `pca`: Calculates the first principal component of the exposure variables.
- `irt`: Uses Item Response Theory to calculate the exposome score.
- `quantile`: Calculates the quantile of the exposure variables.

- `var`: Calculates the variance of the exposure variables.

The `score_column_name` argument is used to set the name of the column to store the exposome score in. Here we will define a score for metals using a variety of different methods and demonstrate their use in association with asthma status.

```
# determine which metals to use
metals <- c("hs_cs_c_Log2", "hs_pb_c_Log2", "hs_mo_c_Log2")

expom <- expom |>
    run_exposome_score(
        exposure_cols = metals,
        score_type = "median",
        score_column_name = "exposome_median_score"
    ) |>
    run_exposome_score(
        exposure_cols = metals,
        score_type = "pca",
        score_column_name = "exposome_pca_score"
    ) |>
    run_exposome_score(
        exposure_cols = metals,
        score_type = "quantile",
        score_column_name = "exposome_quantile_score"
    ) |>
    run_exposome_score(
        exposure_cols = metals,
        score_type = "var",
        score_column_name = "exposome_var_score"
    )
```

We can then associate these exposome scores with asthma status using the `run_association` function, just like we did before. However, this time we specify our `feature_set` to be the exposome scores we just calculated.

```
# Associate exposome scores with outcome
expom <- run_association(
    exposomicset = expom,
    outcome = "hs_asthma",
    source = "exposures",
    feature_set = c(
        "exposome_median_score",
        "exposome_pca_score",
        "exposome_quantile_score",
        "exposome_var_score"
    ),
    action = "add",
    family = "binomial"
)
```

```
plot_association(
    exposomicset = expom,
    source = "exposures",
```

```
        terms = c(
            "exposome_median_score",
            "exposome_pca_score",
            "exposome_quantile_score",
            "exposome_var_score"
        ),
        filter_col = "p.value",
        filter_thresh = 0.05,
        r2_col = "r2"
    )
```

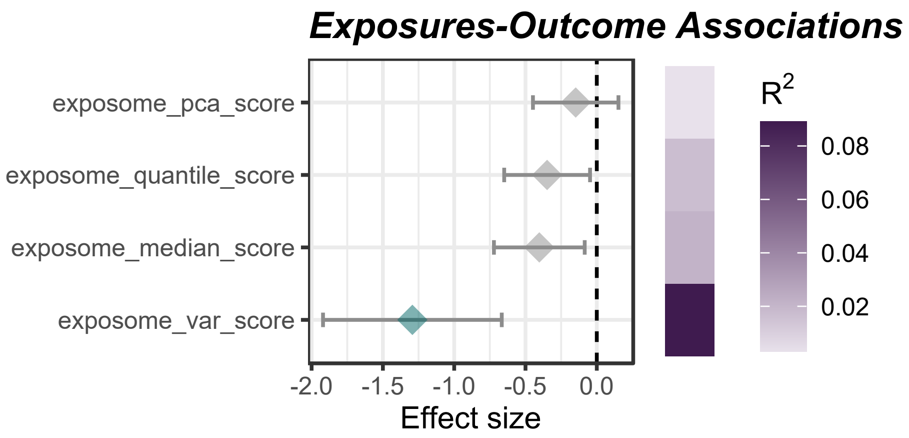

Figure 15: Associations of aerosol exposome scores with asthma status. The variance-based score has the strongest association with asthma status.

7 Differential Abundance

7.1 Differential Abundance

We provide functionality to test for differentially abundant features associated with an outcome across multiple omics layers. This is done using the `run_differential_abundance` function, which fits a model defined by the user (using the `formula` argument) and supports several methods. Here we apply the `limma_trend` method, a widely used approach for analyzing omics data. Users can also specify how features are scaled (e.g. none, quantile, TMM) before fitting.

```
# Run differential abundance analysis
expom <- run_differential_abundance(
    exposomicset = expom,
    formula = ~hs_asthma,
    method = "limma_trend",
    scaling_method = "none",
    action = "add"
)
```

We can summarize the results of the differential abundance analysis with a volcano plot, which highlights features with a high log fold change and that are statistically significant. The `plot_volcano` function generates this visualization, with options to set thresholds for p-values and log fold changes, and to label a subset of top-ranked features. In this example, we use the `feature_clean` column to display interpretable feature names.

Note: we set the `pval_col` to `P.Value` for the purposes of this example, but we recommend keeping the default of `adj.P.Val` to use the adjusted p-values.

```
plot_volcano(
    exposomicset = expom,
    top_n_label = 3,
    feature_col = "feature_clean",
    logFC_thresh = log2(1),
    pval_thresh = 0.05,
    pval_col = "P.Value",
    logFC_col = "logFC",
    nrow = 1
)
```

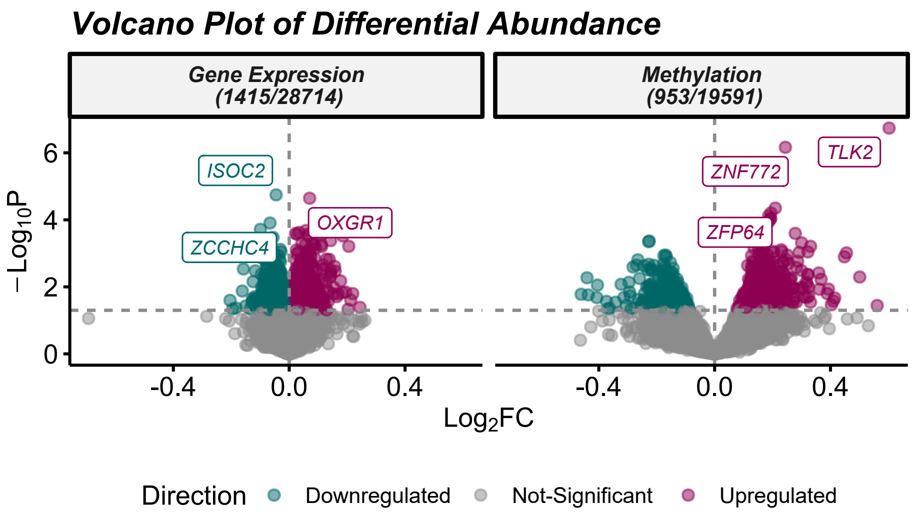

Figure 16: Volcano plot of differentially abundant features across omics layers.

7.2 Sensitivity Analysis

Depending on pre-processing steps, the results of the differential abundance analysis may vary. The `sensitivity_analysis` function is used to perform a sensitivity analysis to determine the robustness of the results. Here we determine if a feature is still differentially abundant if different minimum values, proportions, scaling methods are used, the inclusion of covariates, and after bootstrapping. We then define a stability score based on the number of times a feature is found to be differentially abundant under different conditions as well as the consistency of the effect size:

Where:

$$Stability\ Score = \frac{\sum_i (p_i < \alpha)}{N} * \frac{1}{1 + \frac{\sigma_\beta}{\mu_{|\beta|}}}$$

Where:

- p_i is the p-value for the ith test
- α is the significance threshold
- β is the log fold change
- N is the number of tests
- σ_β is the standard deviation of the effect size estimates
- $\mu_{|\beta|}$ is the mean of the absolute value of the effect size estimates.

The first term captures the proportion of tests that are significant, while the second term captures the consistency of the effect size estimates. The stability score ranges from 0 to 1.

A stability score of 1 indicates that the feature is always found to be differentially abundant, while a stability score of 0 indicates that the feature is never found to be differentially abundant. Besides these, we provide other score metrics as well:

- `presence_rate`: Proportion of runs in which the feature's p-value is below the specified threshold (selection frequency).
- `effect_consistency`: Inverse of the coefficient of variation of log fold-changes; measures effect size stability across runs.
- `stability_score`: Hybrid score combining presence_rate and effect_consistency, capturing reproducibility and signal strength.
- `mean_log_p`: Average of negative log-transformed p-values; represents overall statistical signal strength.
- `logp_weighted_score`: Product of `mean_log_p` and effect_consistency; highlights consistently strong features.
- `sd_logFC`: Standard deviation of log fold-change estimates; quantifies variability of effect sizes.
- `iqr_logFC`: Interquartile range of log fold-changes; provides a robust measure of effect size spread.
- `cv_logFC`: Coefficient of variation of log fold-changes; reflects relative variability of effect size.
- `sign_flip_freq`: Proportion of runs where the sign of the effect size differs from the overall average direction.
- `sd_log_p`: Standard deviation of log-transformed p-values; indicates variability in statistical signal.

```
# Perform Sensitivity Analysis
expom <- run_sensitivity_analysis(
    exposomicset = expom,
    base_formula = ~hs_asthma,
    methods = c("limma_trend"),
    scaling_methods = c("none"),
```

```
    pval_col = "P.Value",
    logfc_col = "logFC",
    logFC_threshold = log2(1),
    pval_threshold = 0.05,
    stability_metric = "stability_score",
    bootstrap_n = 10,
    action = "add"
)
```

```
plot_sensitivity_summary(
    exposomicset = expom,
    stability_score_thresh = 0.8,
    stability_metric = "stability_score"
)
```

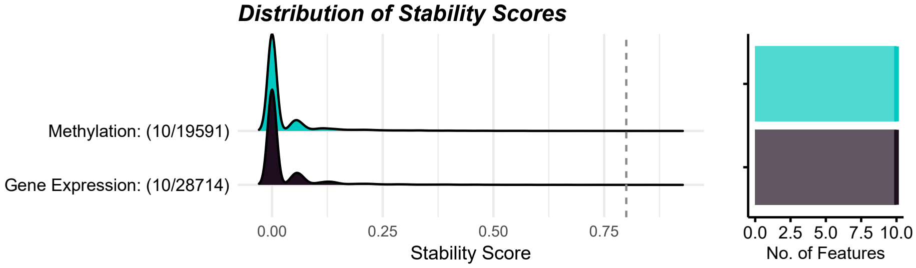

Figure 17: Sensitivity summary highlighting robust features by stability score.

Here we see that very few omics-feature-phenotype associations are stable across bootstrapping runs. Given that this example dataset has a lower sample size, this is not suprising.

8 Multi-Omics Integration

While differential abundance analysis per omics layer can deliver insights into how each omic is associated with a particular outcome, we may want to leverage methods which integrate multiple omics layers. The `run_multiomics_integration` function is used to integrate multiple omics layers. Here we use either `MCIA`, `RGCCA`, `MOFA`, or the `DIABLO` method to integrate omics layers:

- `MCIA`: Multiple Co-inertia Analysis, a method that uses canonical correlation analysis to integrate multiple omics layers. We use the nipalsMCIA algorithm to compute the co-inertia scores from the `nipalsMCIA` package (nipalsMCIA).
- `RGCCA` : Generalized canonical correlation for flexible multi-block integration implemented using the `RGCCA` package (Regularized and Sparse Generalized Ca. . .).
- `MOFA`: Multi-Omics Factor Analysis, a method that uses factor analysis to integrate multiple omics layers. MOFA is implemented using the `MOFA2` package (MOFA2).
- `DIABLO`: Supervised multi-block PLS for outcome-aligned latent factors implemented using the `mixOmics` package (mixOmics).

Here we are interested in integrating our omics layers with the end goal of identifying multi-omics features that are associated with asthma status. So, we will use the `MCIA` method to first identify latent factors representing coordinated biological signals across omics layers. Then, we will identify which omics features greatly contribute to these factors.

```
# Perform multi-omics integration
expom <- run_multiomics_integration(
    exposomicset = expom,
    method = "MCIA",
    n_factors = 5,
    action = "add"
)
```

We can then use `plot_factor_summary` to visualize which omics contribute most to which factors.

```
plot_factor_summary(
    exposomicset = expom,
    midpoint = .6
)
```

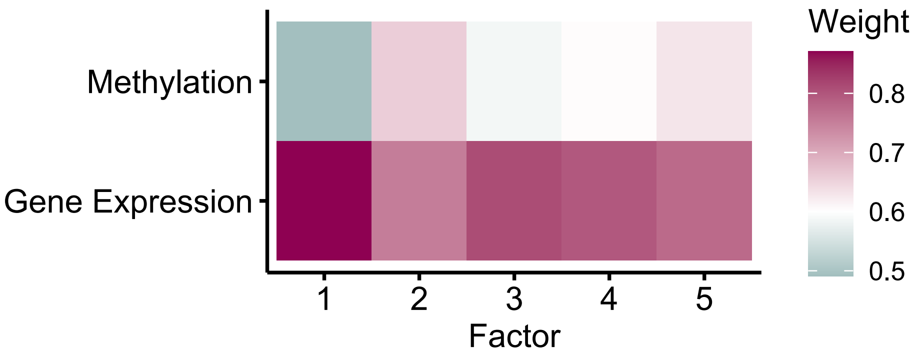

Figure 18: Contribution of each omics layer to DIABLO latent factors.

Here we see that these factors are largely driven by features in the gene expression data.

8.1 Factor Analysis

These methods are designed to identify factors that we can then associate with an outcome variable. Here we will use the `run_association` function to identify factors that are associated with asthma status.

```
# Identify factors that are associated with the outcome
expom <- run_association(
    exposomicset = expom,
    source = "factors",
    outcome = "hs_asthma",
    feature_set = exp_vars,
    action = "add",
    family = "binomial"
)
```

Now let's see if any of our factors are associated with asthma status.

```
plot_association(
    exposomicset = expom,
    source = "factors",
    filter_col = "p.value",
    filter_thresh = 0.05,
    r2_col = "r2"
)
```

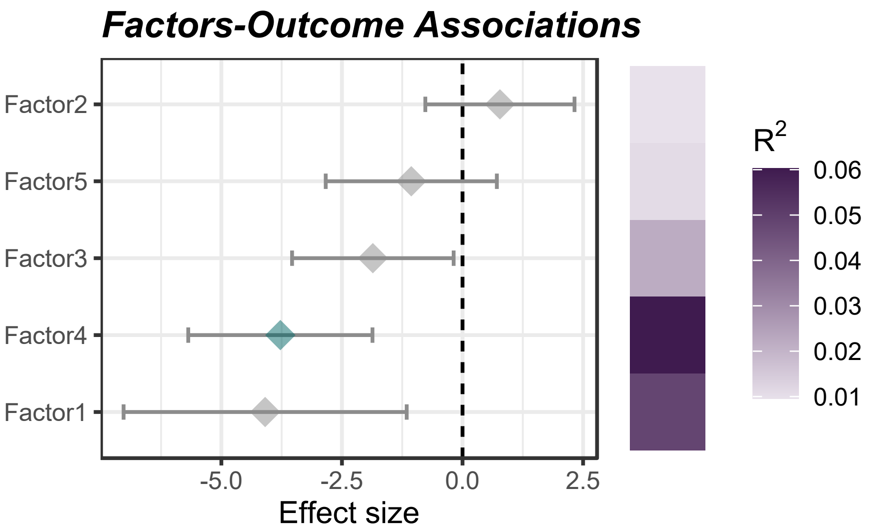

Figure 19: Associations of latent factors with asthma status.

We see that only one factor is associated with our outcome. Factors have loading scores which indicate the strength of the association between the factor and the features. Here we can extract the top features, those in the 95th percentile, per omic, associated with our factors of interest. We set the `pval_col` and `pval_thresh` to filter our association results to grab the factors that pass our thresholds. However, we could specify specific factors with the `factors` argument too.

```
# Extract top features that contribute to a factor
expom <- extract_top_factor_features(
    exposomicset = expom,
    method = "percentile",
    pval_col = "p.value",
    pval_thresh = 0.05,
    percentile = 0.95,
    action = "add"
)
```

We can visualize the top features associated with each factor using the `plot_top_factor_features` function. The `top_n` argument is used to set the number of top features to plot. The factors argument is used to set the factors to plot.

```
plot_top_factor_features(
    exposomicset = expom,
```

```
        top_n = 5,
        feature_col = "feature_clean"
    )
```

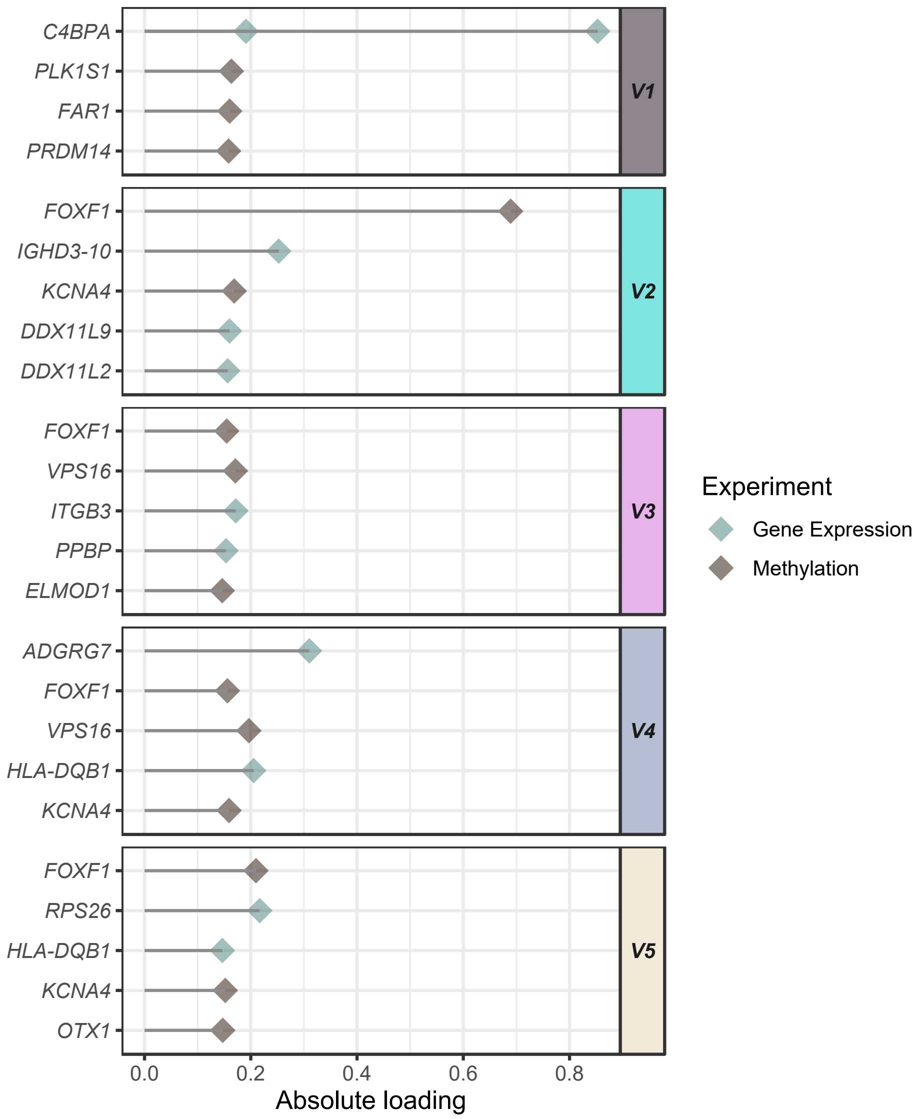

Figure 20: Top-loading features per factor and omics layer.

To identify the common features across factors, we can use the `run_factor_overlap` function. This function will identify the features that are shared across the top factor features. We can then use the `extract_results` function to take a look at how many common features were identified. However, since only one factor was significantly associated with our outcome this will only pull the top factor features from that factor.

```
# Determine which features drive multiple factors
expom <- run_factor_overlap(
  exposomicset = expom)
```

```
extract_results(
  exposomicset = expom,
  result = "multiomics_integration") |>
  pluck("common_top_factor_features") |>
  group_by(exp_name) |>
  reframe(n=n())
## # A tibble: 2 x 2
##   exp_name             n
##   <chr>            <int>
## 1 Gene Expression   1208
## 2 Methylation       1208
```

Here we see that there are about 1000 features in each experiment that are shared across the factors.

Note: The `extract_results` function may also be used to extract the following results:

- `codebook`: exposure metadata and ontology annotations.
- `quality_control`: sample- and variable-level QC summaries.
- `correlation`: pairwise exposure-feature or feature-feature correlations.
- `association`: results from exposure or omics association testing.
- `differential_analysis`: results from differential expression or abundance analyses.
- `multiomics_integration`: latent factors and feature loadings from integration models.
- `network`: exposure–omics or feature-feature network structures.
- `enrichment`: functional enrichment or pathway analysis results.

9 Exposure-Omics Association

9.1 Exposure-Omics Association

Now we have the option to correlate either the top factor features, differentially abundant features, or user-specified omics features (by using a variable map, a data frame with two columns, `exp_name` for the name of the omics assay, and `variable` for the name of the molecular feature) with exposures.

Here we will correlate features driving multiple latent factors with exposures. To grab these features, we will grab them from the metadata in our `MultiAssayExperiment` object. The `correlation_cutoff` is used to set the minimum correlation score, while the `pval_cutoff` is used to set the maximum p-value for the association.

```
# Grab top common factor features and ensure
# feature is renamed to variable for the variable_map
top_factor_features <- expom |>
    extract_results(result = "multiomics_integration") |>
    pluck("common_top_factor_features") |>
    dplyr::select(variable = feature, exp_name)
```

```
# Correlate top factor features with exposures
# Perform correlation analysis between factor features and exposures
expom <- run_correlation(
    exposomicset = expom,
    feature_type = "omics",
    variable_map = top_factor_features,
    exposure_cols = exp_vars,
    action = "add",
    correlation_cutoff = 0.2,
    pval_cutoff = 0.05,
    cor_pval_column = "p.value"
)

# Perform correlation analysis between factor features
expom <- run_correlation(
    exposomicset = expom,
    feature_type = "omics",
    variable_map = top_factor_features,
    exposure_cols = exp_vars,
    feature_cors = TRUE,
    action = "add",
    correlation_cutoff = 0.2,
    pval_cutoff = 0.05,
    cor_pval_column = "p.value"
)
```

We can plot the results of the exposure-omics association analysis using the `plot_correlation_summary` function. Here we set the mode to `summary`, which will plot the number of associations per exposure and feature type. The `feature_type` argument is used to set the type of features to plot. Here we use `omics`, which are the top factor features.

```
plot_correlation_summary(
    exposomicset = expom,
    mode = "summary",
    feature_type = "omics"
)
```

Here we note that gene expression features have a greater number of associations with exposures. Among exposures, we see that aerosols have the greatest number of associations with features, which may reflect the larger number of aerosol variables.

It may also be useful to identify which exposures are correlated with similar molecular features. We can do this with the `plot_circos_correlation` function. This function will plot a circos plot of the exposures and their shared features. The `feature_type` argument is used to set the correlation results to use for the analysis. Here we use the `omics` feature set, which plots the feature-exposure correlation information. The cutoff argument is used to set the minimum number of shared features to plot. Here we set the `shared_cutoff` to 1, which means that only exposures that share at least one feature will be plotted.

```
plot_circos_correlation(
    exposomicset = expom,
    feature_type = "omics",
```

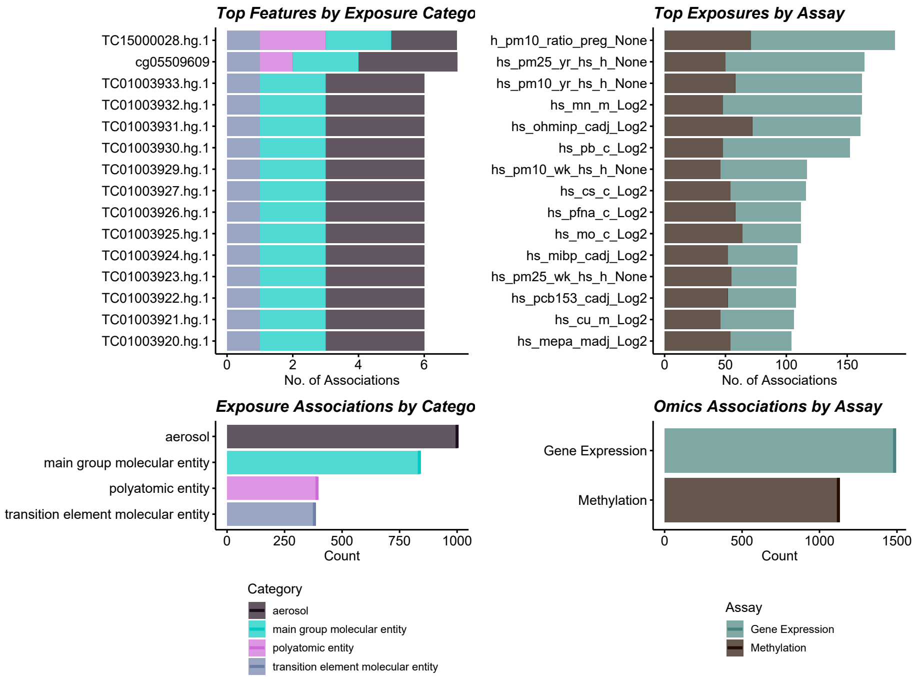

Figure 21: Summary of exposure–omics associations, where aerosols have the greatest absolute number of exposure–omics associations. Exposures are associated with more gene expression features compared to methylation features.

```
    shared_cutoff = 1,
    midpoint = 3
)
```

Here we see that particulate matter exposures are correlated with many of the same features.

9.2 Network Analysis

The `run_create_network` function is used to create a network of exposures and omics features. The correlation results are used to create the network. The `feature_type` argument is used to set the type of features to use for the network. We could choose any of the following:

- `degs`: Differentially abundant features correlated with exposures.
- `factors`: Factor features correlated with exposures.
- `omics`: User specified omics features correlated with exposures.

Here we create networks based on two correlation tables generated above:

- `omics`: Correlations between omics and exposure variables.
- `omics_feature_cor`: Correlation just between the omics features.

This allows us to quantify a bipartite graph between exposures and omics features and a graph just between the omics features.

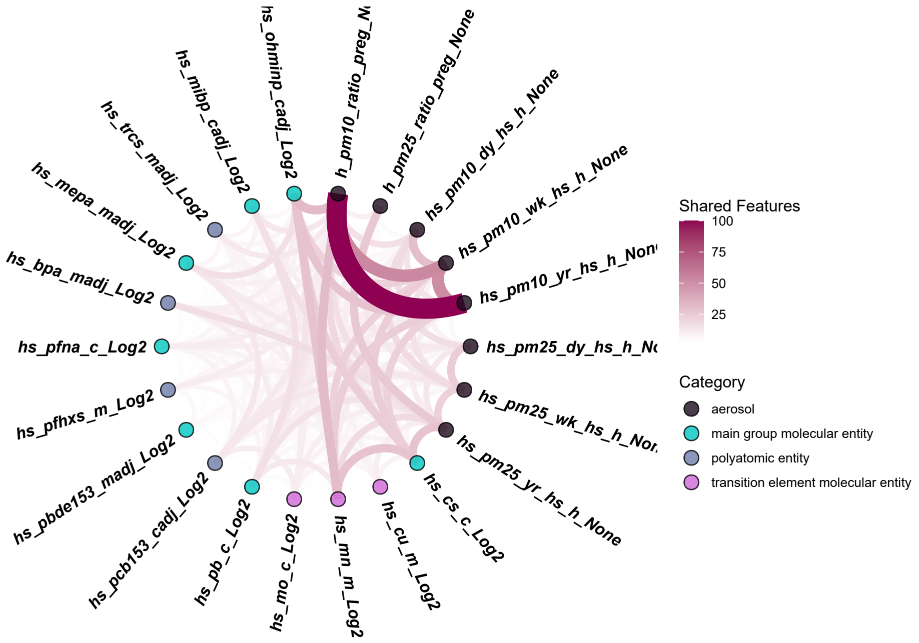

Figure 22: Circos plot of exposures sharing correlated molecular features.

```
# Create omics within feature correlation network
expom <- run_create_network(
    exposomicset = expom,
    feature_type = "omics_feature_cor",
    action = "add"
)

# Create omics-exposure correlation network
expom <- run_create_network(
    exposomicset = expom,
    feature_type = "omics",
    action = "add"
)
```

To plot the network we can use the `plot_network` function. The network argument is used to set the type of network to plot. The `top_n_nodes` argument is used to set the number of nodes to plot. The `node_color_var` argument is used to set the variable to color the nodes by. The `label` argument is used to set whether or not to label the nodes. We can label the top n nodes, where the default is 5 nodes based on centrality. Additionally, we can choose to label certain nodes using the `nodes_to_label` argument. We can also include the network statistics using the `include_stats` argument.

```
plot_network(
    exposomicset = expom,
    network = "omics",
    top_n_nodes = 50,
    include_stats = TRUE,
    cor_thresh = 0.2,
```

```
    node_color_var = "group",
    label = TRUE,
    label_top_n = 5
)
```

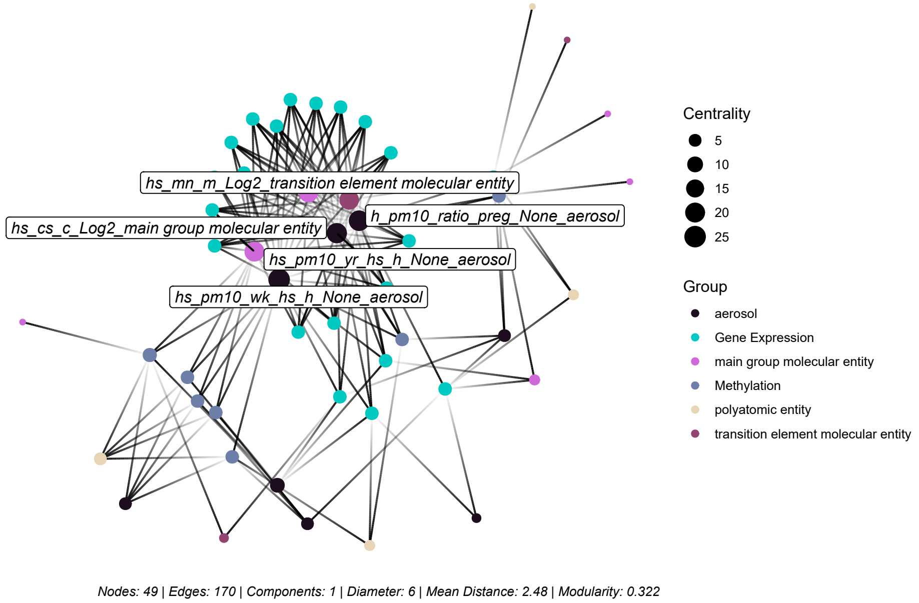

Figure 23: Bipartite network of exposures and correlated omics features where node labels reflect most central nodes.

Here we note that the network is highly connected, with particulate matter exposures having the most connections to omics features. To briefly explain the metrics mentioned:

- **Nodes**: Number of nodes included in the graph.
- **Edges**: Number of connections between nodes in the graph.
- **Components**: Number of disconnected subgraphs.
- **Diameter**: The longest shortest path between any two nodes.
- **Mean Distance**: The average shortest path between any two nodes.
- **Modularity**: A measure of the division in the graph, where higher modularity indicates that nodes within the same community are more densely connected compared to nodes in different communities.

9.3 Exposure-Omics Impact Network Analysis

The bipartite graph above describes how exposures are associated with omics features. However, this does not describe if exposures are associated with features that are more central in the feature-only graph (i.e. are exposures associated with features that are more central or peripheral to the graph?). To answer this question, we can use the `run_exposure_impact` function to calculate centrality metrics for the omics features associated with a given expo-

sure. Centrality is computed on the feature-only graph. Centrality metrics are used to identify features that are more central to the network and may be more important in the context of the exposure-omics relationships. The centrality metrics included are:

- **Mean Betweenness Centrality**: Measures how often a node lies on the shortest path between other nodes where high values indicate potential intermediates.
- **Mean Closeness Centrality**: Measures how close a node is to all others in the network where high values indicate faster access to all nodes.
- **Mean Degree**: The average number of direct connections or edges a node has.
- **Mean Eigenvector Centrality**: Measures how well-connected a node is and how well-connected its neighbors are, where high values suggest influence in a well-connected cluster.

```
# Run exposure-omics impact analysis
expom <- run_exposure_impact(
    exposomicset = expom,
    feature_type = "omics"
)
```

To plot the results of the exposure-omics impact analysis, we can use the `plot_exposure_impact` function. We set `feature_type` to `omics`, which means that we are plotting the network metrics of the omics features correlated with exposures. The `min_per_group` argument is used to set the minimum number of features per group to plot. This is useful for filtering out exposures that do not have enough associated features.

```
plot_exposure_impact(
    exposomicset = expom,
    feature_type = "omics",
    min_per_group = 10
)
```

Here we see that prenatal BPA and childhood Monoisobutyl phthalate exposure is associated with omics features that are central to our network, despite not having the greatest number of features associated with it. This suggests that these exposures may have a significant impact on the omics features, even if it is not associated with as many features as some of the other exposures.

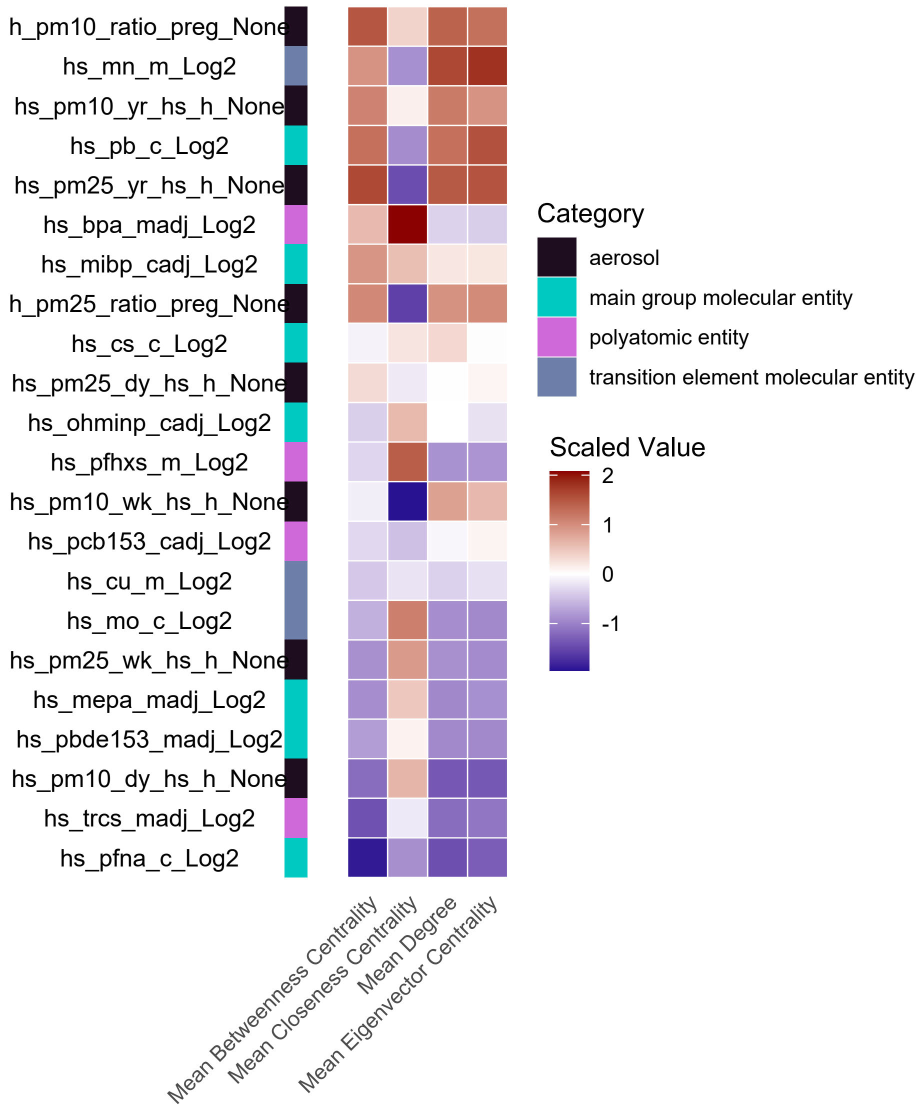

Figure 24: Network centrality metrics of omics features associated with each exposure.

10 Enrichment Analysis

Enrichment analysis tests whether a set of molecular features (e.g. differentially abundant genes, metabolites, etc.) is over-represented in a predefined biological process. The benefit of grouping our exposures into categories is that we can now determine how broad categories of exposures are tied to biological processes. The `run_enrichment` function can perform enrichment analysis on the following feature types:

- `degs`: Differentially abundant features.
- `degs_robust`: Robust differentially abundant features from the sensitivity analysis.
- `omics`: User chosen features.
- `factor_features`: Multi-omics factor features either from `factor_type = “common_top_factor_features”` or `“top_factor_features”`.

- `degs_cor`: Differentially abundant features correlated with a set of exposures.
- `omics_cor`: User chosen features correlated with a set of exposures.
- `factor_features_cor`: Multi-omics factor features correlated with a set of exposures.

Here we will run enrichment analysis on factor features correlated with exposures. We specify `feature_col` to represent the column in our feature metadata with IDs that can be mapped (i.e. gene names). We will be performing Gene ontology enrichment powered by the `fenr` package (Fenr, 2025). Note that we specify a `clustering_approach.` This will cluster our enrichment terms by the molecular feature overlap.

```
# Run enrichment analysis on factor features correlated with exposures
expom <- run_enrichment(
    exposomicset = expom,
    feature_type = c("omics_cor"),
    feature_col = "feature_clean",
    db = c("GO"),
    species = "goa_human",
    fenr_col = "gene_symbol",
    padj_method = "none",
    pval_thresh = 0.05,
    min_set = 1,
    max_set = 800,
    clustering_approach = "diana",
    action = "add"
)
```

10.1 Enrichment Visualizations

To visualize our enrichment results we provide several options:

- dot'plot: A dot plot showing the top enriched terms. The size of the dots represents the number of features associated with the term, while the color represents the significance of the term.
- `cnet`: A network plot showing the relationship between features and enriched terms.
- `network`: A network plot showing the relationship between enriched terms.
- `heatmap`: A heatmap showing the relationship between features and enriched terms.
- `summary`: A summary figure of the enrichment results.

10.1.1 Enrichment Summary

To summarize the enrichment results, we can use the `plot_enrichment` function with the `plot_type` argument set to `summary.` This will plot a summary of the enrichment results, showing:

- The number of exposure categories per enrichment term group.
- The number of features driving the enrichment term group.
- A p-value distribution of the enrichment term group.
- The number of terms in the enrichment term group.

- The total number of terms per experiment name.
- The overlap in enrichment terms between experiments (i.e. between gene expression and methylation).

```
plot_enrichment(
    exposomicset = expom,
    feature_type = "omics_cor",
    plot_type = "summary"
)
```

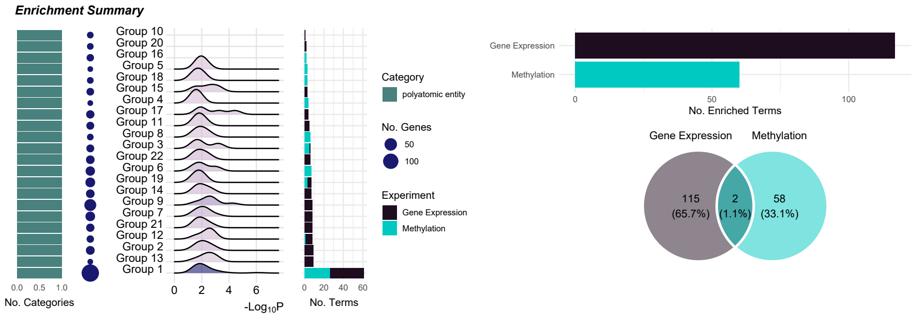

Figure 25: Summary of enriched GO terms grouped by overlap and exposure category.

Here we see that it is just the features associated with "polyatomic entity" exposures that seem to be enriched. Additionally, there appears to be a few overlapping terms between methylation and gene expression results.

10.1.2 DotPlot

By setting the `plot_type` to dotplot we can create a dotplot to show which omics and which exposure categories are associated with which terms. By specifying the `top_n_genes` we can add the most frequent features in that particular enrichment term group.

```
plot_enrichment(
    exposomicset = expom,
    feature_type = "omics_cor",
    plot_type = "dotplot",
    top_n = 10,
    add_top_genes = TRUE,
    top_n_genes = 5
)
```

10.1.3 Term Network Plot

We can set the `plot_type` to `network` to understand how our enrichment terms are individually connected.

```
plot_enrichment(
    exposomicset = expom,
    feature_type = "omics_cor",
    plot_type = "network",
```

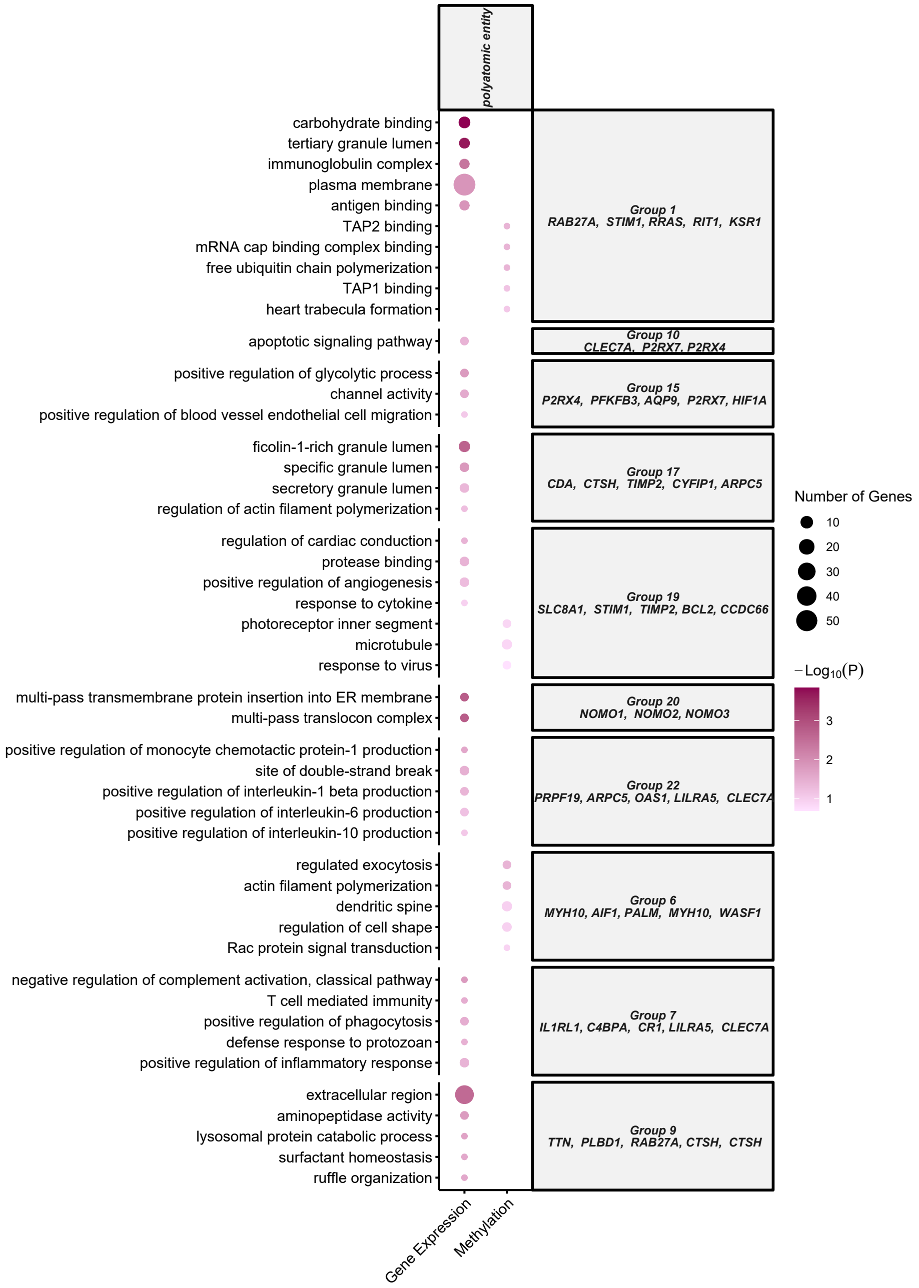

Figure 26: Dotplot of top enriched GO terms by omics layer and exposure category.

```
    label_top_n = 2
)
```

At the individual term level, we see that they differ by omics layer, with the gene expression driving terms related to vesicle dynamics and the methylation data driving terms related to development.

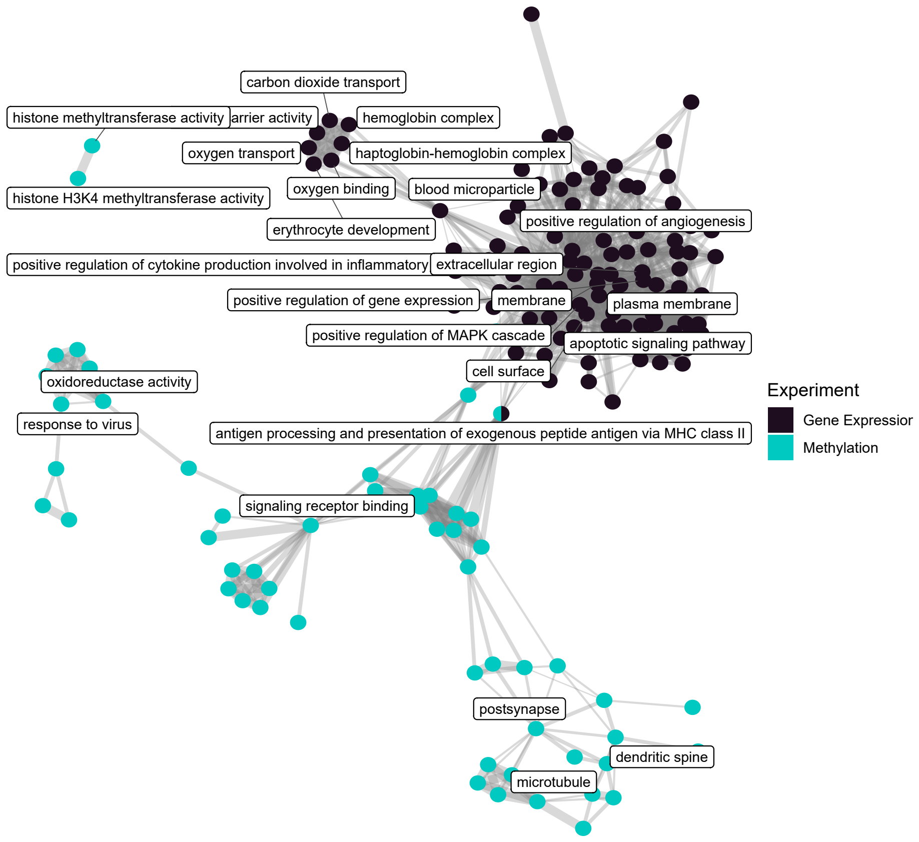

Figure 27: Network of enriched GO terms connected by shared genes.

10.1.4 Heatmap

Setting the `plot_type` to `heatmap` can help us understand which genes are driving the enrichment terms. We have the additional benefit of being able to color our tiles by the Log_2_Fold Change from our differential abundance testing. Here we will examine group 15 as an example.

```
plot_enrichment(
    exposomicset = expom,
    feature_type = "omics_cor",
    go_groups = "Group 15",
    plot_type = "heatmap",
    heatmap_fill = TRUE,
    feature_col = "feature_clean"
)
```

Here we see that methylation features have the strongest fold change with respect to asthma status. Interestingly, we see that gene expression of *HIF1A*, a low-oxygen response transcription factor, is downregulated in asthma and correlated with exposures in the polyatomic entity category.

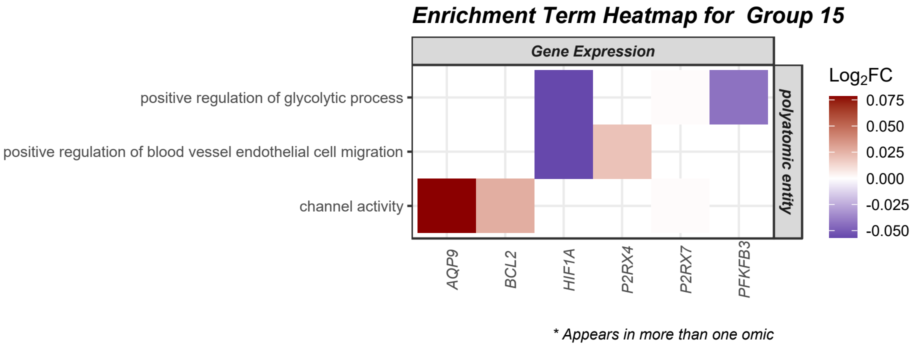

Figure 28: Heatmap of genes driving enriched GO terms (Group 15) with log2 fold-change overlay.

10.1.5 Cnet Plot

Another way to visualize this information is with the `cnet` plot, where the enrichment terms are connected to the genes driving them.

```
plot_enrichment(
    exposomicset = expom,
    feature_type = "omics_cor",
    go_groups = "Group 15",
    plot_type = "cnet",
    feature_col = "feature_clean"
)
```

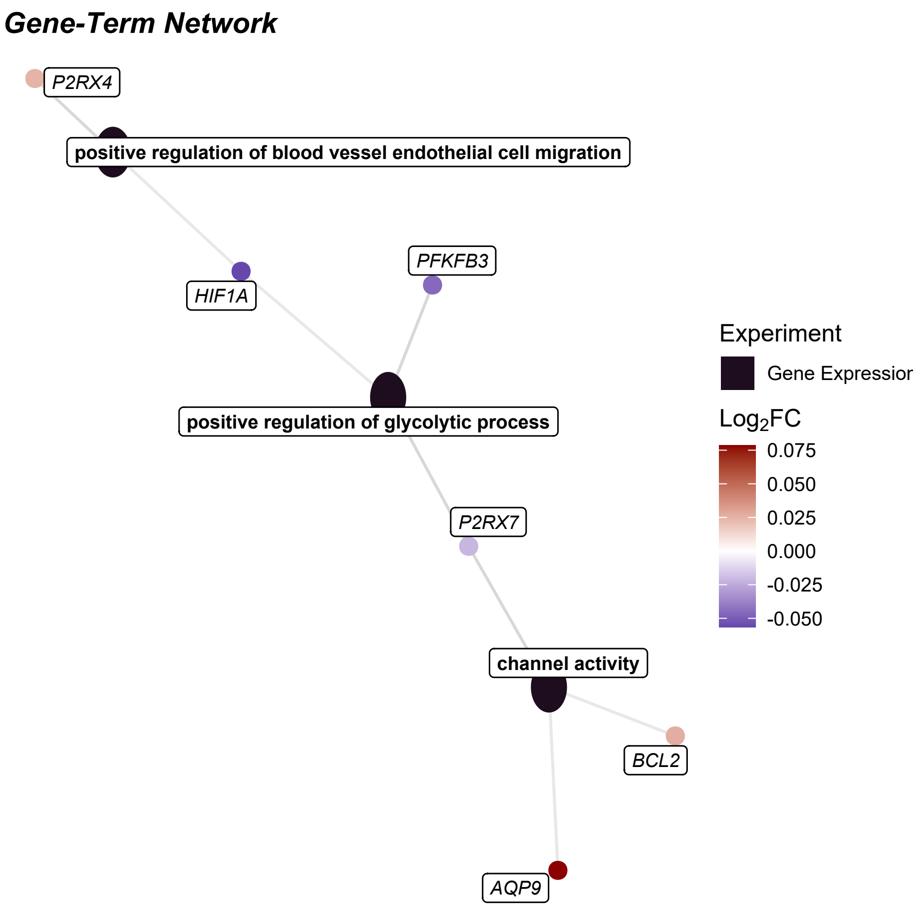

Figure 29: Cnet plot linking enriched terms to contributing genes (Group 15).

11 Custom Analysis

We provide functionality to access the underlying data in the MultiAssayExperiment object and to construct tibbles for your own analysis:

- `pivot_sample`: Pivot the sample data to a tibble with samples as rows and exposures as columns.
- `pivot_feature`: Pivot the feature metadata to a tibble with features as rows and feature metadata as columns.
- `pivot_exp`: Pivot the sample and experiment assay data to a tibble with samples as rows and sample metadata as columns. Additionally, there will be a column for values for specified features in specified assays.

11.1 Pivot Sample

Let's check out the `pivot_sample` function. This function pivots the sample data to a tibble with samples as rows and exposures as columns.

```
# Pivot sample data to a tibble
expom |>
    pivot_sample() |>
    head()
## # A tibble: 6 x 358
##   .sample h_abs_ratio_preg_Log h_no2_ratio_preg_Log hs_no2_dy_hs_h_Log
##   <chr>                  <dbl>                <dbl>              <dbl>
## 1 s224                 1.01                    3.21               3.32
## 2 s873                -0.00513                 2.48               3.32
## 3 s1019               -0.0663                  2.89               3.08
## 4 s725                 0.354                   3.18               2.69
## 5 s1025                0.0545                  2.76               3.56
## 6 s242                 0.338                   2.92               3.80
## # i 354 more variables: hs_no2_wk_hs_h_Log <dbl>, hs_no2_yr_hs_h_Log <dbl>,
## #   hs_pm25abs_dy_hs_h_Log <dbl>, hs_pm25abs_wk_hs_h_Log <dbl>,
## #   hs_pm25abs_yr_hs_h_Log <dbl>, h_accesslines300_preg_dic0 <dbl>,
## #   h_accesspoints300_preg_Log <dbl>, h_builtdens300_preg_Sqrt <dbl>,
## #   h_connind300_preg_Sqrt <dbl>, h_fdensity300_preg_Log <dbl>,
## #   h_frichness300_preg_None <dbl>, h_landuseshan300_preg_None <dbl>,
## #   h_popdens_preg_Sqrt <dbl>, h_walkability_mean_preg_None <dbl>, ...
```

We could use this functionality to count the number of asthmatics per sex:

```
expom |>
    pivot_sample() |>
    group_by(hs_asthma, e3_sex_None) |>
    summarise(n = n())
## # A tibble: 4 x 3
## # Groups:   hs_asthma [2]
##   hs_asthma e3_sex_None     n
##       <dbl> <fct>       <int>
## 1         0 female         51
## 2         0 male           44
## 3         1 female          7
## 4         1 male            6
```

11.2 Pivot Feature

The `pivot_feature` function pivots the feature metadata to a tibble with features as rows and feature metadata as columns. This can be useful for exploring the feature metadata in a more flexible way.

```
# Pivot feature data to a tibble
expom |>
    pivot_feature() |>
    head()
## # A tibble: 6 x 42
##   .exp_name     .feature transcript_cluster_id probeset_id seqname strand  start
##   <chr>         <chr>    <chr>                 <chr>       <chr>   <chr>   <int>
## 1 Gene Express~ TC01000~ TC01000001.hg.1       TC01000001~ chr1    +       11869
## 2 Gene Express~ TC01000~ TC01000002.hg.1       TC01000002~ chr1    +       29554
```

```
## 3 Gene Express~ TC01000~ TC01000003.hg.1       TC01000003~ chr1    +       69091
## 4 Gene Express~ TC01000~ TC01000005.hg.1       TC01000005~ chr1    +      317811
## 5 Gene Express~ TC01000~ TC01000007.hg.1       TC01000007~ chr1    +      321146
## 6 Gene Express~ TC01000~ TC01000009.hg.1       TC01000009~ chr1    +      367640
## # i 35 more variables: stop <int>, total_probes <int>, gene_assignment <chr>,
## #   mrna_assignment <chr>, notes <chr>, phase <chr>, TC_size <dbl>,
## #   TSS_Affy <int>, EntrezeGeneID_Affy <chr>, GeneSymbol_Affy <chr>,
## #   GeneSymbolDB <chr>, GeneSymbolDB2 <chr>, mrna_ID <chr>, mrna_DB <chr>,
## #   mrna_N <int>, notesYN <chr>, geneYN <chr>, genes_N <dbl>, CallRate <dbl>,
## #   fill <chr>, feature_clean <chr>, Forward_Sequence <chr>, SourceSeq <chr>,
## #   Random_Loci <chr>, Methyl27_Loci <chr>, UCSC_RefGene_Name <chr>, ...
```

We could use this functionality to count the number of features per omics layer, or to filter features based on their metadata. For example, we can count the number of features per omics layer:

```
# Count the number of features per omic layer
expom |>
    pivot_feature() |>
    group_by(.exp_name) |>
    summarise(n = n())
## # A tibble: 2 x 2
##   .exp_name           n
##   <chr>           <int>
## 1 Gene Expression 28714
## 2 Methylation     19591
```

11.3 Pivot Experiment

Now if we want to grab assay data from a particular experiment, we can do that with `pivot_exp`. Let's try grabbing assay values for the `cg25330361` probe (probe for *CYP2E1*) in the `Methylation` experiment:

```
# Pivot experiment data to a tibble
expom |>
    pivot_exp(
        exp_name = "Methylation",
        features = "cg25330361"
    ) |>
    head()
## # A tibble: 6 x 376
##   exp_name    .feature   .sample counts Forward_Sequence   SourceSeq Random_Loci
##   <chr>       <chr>      <chr>    <dbl> <chr>              <chr>     <chr>
## 1 Methylation cg25330361 s224     0.826 TCGGCGACTGTGCGGGA~ CGGGAGAG~ ""
## 2 Methylation cg25330361 s873     1.16  TCGGCGACTGTGCGGGA~ CGGGAGAG~ ""
## 3 Methylation cg25330361 s1019    0.974 TCGGCGACTGTGCGGGA~ CGGGAGAG~ ""
## 4 Methylation cg25330361 s725     1.06  TCGGCGACTGTGCGGGA~ CGGGAGAG~ ""
## 5 Methylation cg25330361 s1025    0.656 TCGGCGACTGTGCGGGA~ CGGGAGAG~ ""
## 6 Methylation cg25330361 s242     0.968 TCGGCGACTGTGCGGGA~ CGGGAGAG~ ""
## # i 369 more variables: Methyl27_Loci <chr>, UCSC_RefGene_Name <chr>,
## #   UCSC_RefGene_Accession <chr>, UCSC_RefGene_Group <chr>, Phantom <chr>,
```

```
## #   DMR <chr>, Enhancer <chr>, HMM_Island <chr>, Regulatory_Feature_Name <chr>,
## #   Regulatory_Feature_Group <chr>, DHS <chr>, feature_clean <chr>,
## #   h_abs_ratio_preg_Log <dbl>, h_no2_ratio_preg_Log <dbl>,
## #   hs_no2_dy_hs_h_Log <dbl>, hs_no2_wk_hs_h_Log <dbl>,
## #   hs_no2_yr_hs_h_Log <dbl>, hs_pm25abs_dy_hs_h_Log <dbl>, ...
```

We can use this functionality to create custom plots or analyses based on the exposure and feature data. For example, we can plot the methylation levels of *CYP2E1* by asthma status:

```
expom |>
    pivot_exp(
        exp_name = "Methylation",
        features = "cg25330361"
    ) |>
    ggplot(aes(
        x = as.character(hs_asthma),
        y = log2(counts + 1),
        color = as.character(hs_asthma),
        fill = as.character(hs_asthma)
    )) +
    geom_boxplot(alpha = 0.5) +
    geom_jitter(alpha = 0.1) +
    ggpubr::geom_pwc(label = "{p.adj.format}{p.adj.signif}") +
    theme_minimal() +
    ggpubr::rotate_x_text(angle = 45) +
    ggsci::scale_color_cosmic() +
    ggsci::scale_fill_cosmic() +
    labs(
        x = "",
        y = expression(Log[2] * "Abd."),
        fill = "Asthma Status",
        color = "Asthma Status"
    )
```

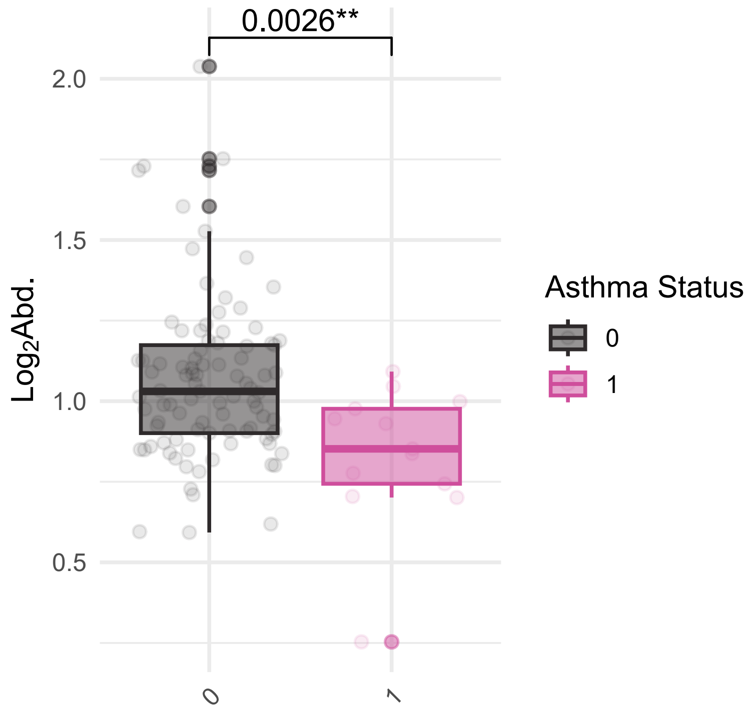

Figure 30: CYP2E1 methylation levels (probe cg25330361) by asthma status.

12 Pipeline Summary

To summarize the steps we have taken in this analysis, we can use the `run_pipeline_summary` function. This function will provide a summary of the steps taken in the analysis. We can set `console_print` to `TRUE` to print the summary to the console. Setting `include_notes` to `TRUE` will include notes on the steps taken in the analysis.

```
# Run the pipeline summary
expom |>
    run_pipeline_summary(console_print = TRUE, include_notes = TRUE)
```

12.1 Saving Results

We can export the results in our `MultiAssayExperiment` to an Excel spreadsheet using the `extract_results_excel` function. Here we add all of our results to the Excel file, but we can choose certain results by changing the `result_types` argument.

```
extract_results_excel(
    exposomicset = expom,
    file = "./supplementary_results.xlsx",
    result_types = "all"
)
```

12.2 Session Info

```
sessionInfo()
## R version 4.5.1 (2025-06-13 ucrt)
## Platform: x86_64-w64-mingw32/x64
## Running under: Windows 11 x64 (build 26200)
##
## Matrix products: default
##   LAPACK version 3.12.1
##
## locale:
## [1] LC_COLLATE=English_United States.utf8
## [2] LC_CTYPE=English_United States.utf8
## [3] LC_MONETARY=English_United States.utf8
## [4] LC_NUMERIC=C
## [5] LC_TIME=English_United States.utf8
##
## time zone: America/New_York
## tzcode source: internal
##
## attached base packages:
## [1] stats4    stats     graphics  grDevices utils     datasets  methods
## [8] base
##
## other attached packages:
##  [1] tidyexposomics_1.1.2        MultiAssayExperiment_1.36.2
##  [3] SummarizedExperiment_1.40.0 Biobase_2.70.0
##  [5] GenomicRanges_1.62.1        Seqinfo_1.0.0
##  [7] IRanges_2.44.0              S4Vectors_0.48.0
##  [9] BiocGenerics_0.56.0         generics_0.1.4
## [11] MatrixGenerics_1.22.0       matrixStats_1.5.0
## [13] lubridate_1.9.5             forcats_1.0.1
## [15] stringr_1.6.0               dplyr_1.2.1
## [17] purrr_1.2.2                 readr_2.2.0
## [19] tidyr_1.3.2                 tibble_3.3.0
## [21] ggplot2_4.0.3               tidyverse_2.0.0
## [23] BiocStyle_2.38.0
##
## loaded via a namespace (and not attached):
##   [1] naniar_1.1.0          httr_1.4.8            RColorBrewer_1.1-3
##   [4] doParallel_1.0.17     ggsci_5.1.0           dynamicTreeCut_1.63-1
##   [7] tools_4.5.1           doRNG_1.8.6.3         backports_1.5.1
##  [10] utf8_1.2.6            R6_2.6.1              DT_0.34.0
##  [13] GetoptLong_1.1.1      withr_3.0.3           gridExtra_2.3.1
##  [16] cli_3.6.5             factoextra_2.2.0      Cairo_1.7-0
##  [19] RGCCA_3.0.3           labeling_0.4.3        S7_0.2.1
##  [22] randomForest_4.7-1.2  ggridges_0.5.7        foreign_0.8-90
##  [25] parallelly_1.48.0     itertools_0.1-3       limma_3.66.0
##  [28] rstudioapi_0.19.0     RSQLite_3.53.3        FNN_1.1.4.1
##  [31] shape_1.4.6.1         vroom_1.7.1           car_3.1-5
##  [34] dendextend_1.19.1     Matrix_1.7-4          abind_1.4-8
```

```
##  [37] lifecycle_1.0.5       yaml_2.3.12           edgeR_4.8.2
##  [40] snakecase_0.11.1      carData_3.0-6         recipes_1.3.3
##  [43] SparseArray_1.10.8    BiocFileCache_3.0.0   Rtsne_0.17
##  [46] grid_4.5.1            blob_1.3.0            promises_1.5.0
##  [49] crayon_1.5.3          lattice_0.22-7        magick_2.9.1
##  [52] pillar_1.11.1         knitr_1.51            ComplexHeatmap_2.26.1
##  [55] rjson_0.2.23          corpcor_1.6.10        future.apply_1.20.2
##  [58] mixOmics_6.34.0       codetools_0.2-20      glue_1.8.0
##  [61] ggvenn_0.1.19         data.table_1.18.2.1   vctrs_0.7.3
##  [64] png_0.1-9             Rdpack_2.6.6          gtable_0.3.6
##  [67] assertthat_0.2.1      cachem_1.1.0          gower_1.0.2
##  [70] xfun_0.60             rbibutils_2.4.1       S4Arrays_1.10.1
##  [73] mime_0.13             prodlim_2026.03.11    tidygraph_1.3.1
##  [76] pracma_2.4.6          survival_3.8-3        timeDate_4052.112
##  [79] nipalsMCIA_1.8.1      iterators_1.0.14      tinytex_0.60
##  [82] hardhat_1.4.3         lava_1.9.2            statmod_1.5.2
##  [85] ipred_0.9-15          nlme_3.1-168          fenr_1.8.2
##  [88] bit64_4.8.2           filelock_1.0.3        Deriv_4.3.0
##  [91] otel_0.2.0            rpart_4.1.24          colorspace_2.1-3
##  [94] DBI_1.3.0             Hmisc_5.2-6           nnet_7.3-20
##  [97] tidyselect_1.2.1      bit_4.6.0             compiler_4.5.1
## [100] curl_7.0.0            tidyHeatmap_1.13.1    httr2_1.3.0
## [103] htmlTable_2.5.0       DelayedArray_0.36.0   bookdown_0.47
## [106] checkmate_2.3.4       scales_1.4.0          digest_0.6.39
## [109] rmarkdown_2.31        XVector_0.50.0        htmltools_0.5.9
## [112] pkgconfig_2.0.3       base64enc_0.1-6       dbplyr_2.6.0
## [115] fastmap_1.2.0         rlang_1.3.0           GlobalOptions_0.1.4
## [118] htmlwidgets_1.6.4     shiny_1.14.0          ggh4x_0.3.1
## [121] farver_2.1.2          jsonlite_2.0.0        BiocParallel_1.44.0
## [124] ModelMetrics_1.2.2.2  magrittr_2.0.4        Formula_1.2-5
## [127] patchwork_1.3.2       Rcpp_1.1.1            ggnewscale_0.5.2
## [130] viridis_0.6.5         visdat_0.6.0          stringi_1.8.7
## [133] pROC_1.19.0.1         ggraph_2.2.2          MASS_7.3-65
## [136] plyr_1.8.9            parallel_4.5.1        listenv_1.0.0
## [139] ggrepel_0.9.8         graphlayouts_1.2.5    splines_4.5.1
## [142] hms_1.1.4             circlize_0.4.18       locfit_1.5-9.12
## [145] igraph_2.3.3          ggpubr_1.0.0          ranger_0.18.0
## [148] ggsignif_0.6.4        rngtools_1.5.2        reshape2_1.4.5
## [151] tidybulk_2.0.1        evaluate_1.0.5        BiocManager_1.30.27
## [154] tzdb_0.5.0            foreach_1.5.2         missForest_1.6.1
## [157] tweenr_2.0.3          httpuv_1.6.17         polyclip_1.10-7
## [160] future_1.75.0         clue_0.3-68           BiocBaseUtils_1.12.0
## [163] ggforce_0.5.0         janitor_2.2.1         broom_1.0.13
## [166] xtable_1.8-8          RSpectra_0.16-2       rstatix_1.1.0
## [169] later_1.4.8           viridisLite_0.4.3     class_7.3-23
## [172] rARPACK_0.11-0        memoise_2.0.1         ellipse_0.5.0
## [175] densityClust_0.3.3    cluster_2.1.8.2       timechange_0.4.0
## [178] globals_0.19.1        caret_7.0-1
```

12.3 References

Chung, M. K., House, J. S., Akhtari, F. S., Makris, K. C., Langston, M. A., Islam, K. T., Holmes, P., Chadeau-Hyam, M., Smirnov, A. I., Du, X., Thessen, A. E., Cui, Y., Zhang, K., Manrai, A. K., Motsinger-Reif, A., Patel, C. J., & Members of the Exposomics Consortium. (2024). Decoding the exposome: data science methodologies and implications in exposome-wide association studies (ExWASs). *Exposome, 4*(1), osae001. https://doi.org/10.1093/exposome/osae001

Gierlinski M. fenr: Fast functional enrichment for interactive applications. Bioconductor 2025, DOI: 10.18129/B9.bioc.fenr.

Maitre, L., Guimbaud, J.-B., Warembourg, C., Güil-Oumrait, N., Petrone, P. M., Chadeau-Hyam, M., Vrijheid, M., Basagaña, X., Gonzalez, J. R., & Exposome Data Challenge Participant Consortium. (2022). State-of-the-art methods for exposure-health studies: Results from the exposome data challenge event. *Environment International, 168*(107422), 107422. https://doi.org/10.1016/j.envint.2022.107422

Miller, G. W., & Banbury Exposomics Consortium. (2025). Integrating exposomics into biomedicine. *Science, 388*(6745), 356–358. https://doi.org/10.1126/science.adr0544

Rohart F, Gautier B, Singh A, Lê Cao KA. mixOmics: An R package for 'omics feature selection and multiple data integration. PLoS Comput Biol. 2017 Nov 3;13(11):e1005752. doi: 10.1371/journal.pcbi.1005752. PMID: 29099853; PMCID: PMC5687754.

Argelaguet R, Velten B, Arnol D, Dietrich S, Zenz T, Marioni JC, Buettner F, Huber W, Stegle O (2018). "Multi-Omics Factor Analysis—a framework for unsupervised integration of multi-omics data sets." Molecular Systems Biology, 14. doi:10.15252/msb.20178124.

Ramos M, Schiffer L, Re A, Azhar R, Basunia A, Rodriguez Cabrera C, Chan T, Chapman P, Davis S, Gomez-Cabrero D, Culhane A, Haibe-Kains B, Hansen K, Kodali H, Louis M, Mer A, Reister M, Morgan M, Carey V, Waldron L (2017). "Software For The Integration Of Multi-Omics Experiments In Bioconductor." Cancer Research, 77(21), e39-42. doi:10.1158/0008-5472.CAN-17-0344, https://cancerres.aacrjournals.org/content/77/21/e39.

Mattessich M, Reyna J, Aron E, Ay F, Kilmer M, Kleinstein S, Konstorum A (2025). "nipalsMCIA: flexible multi-block dimensionality reduction in R via nonlinear iterative partial least squares." Bioinformatics. doi:10.1093/bioinformatics/btaf015.

Girka F, Camenen E, Peltier C, Gloaguen A, Guillemot V, Le Brusquet L, Tenenhaus A (2025). RGCCA: Regularized and Sparse Generalized Canonical Correlation Analysis for Multiblock Data. R package version 3.0.3, https://CRAN.R-project.org/package=RGCCA.

Wild, C. P. (2005). Complementing the genome with an "exposome": the outstanding challenge of environmental exposure measurement in molecular epidemiology. *Cancer Epidemiology, Biomarkers & Prevention, 14*(8), 1847–1850. https://doi.org/10.1158/1055-9965.EPI-05-0456
